# New York State Climate Impacts Assessment Chapter 05: Ecosystems

**DOI:** 10.1111/nyas.15203

**Published:** 2024-12-09

**Authors:** Sheila S. Hess, Douglas A. Burns, F. Garrett Boudinot, Carrie Brown‐Lima, Jason Corwin, John D. Foppert, George R. Robinson, Kevin C. Rose, Matthew D. Schlesinger, Rebecca L. Shuford, Drake Bradshaw, Amanda Stevens

**Affiliations:** ^1^ CC Environment and Planning Batavia New York USA; ^2^ New York Water Science Center United States Geological Survey Troy New York USA; ^3^ Department of Ecology and Evolutionary Biology Cornell University Ithaca New York USA; ^4^ Department of Natural Resources and the Environment Cornell University Ithaca New York USA; ^5^ Department of Indigenous Studies University at Buffalo Buffalo New York USA; ^6^ Department of Forestry Paul Smith's College Paul Smiths New York USA; ^7^ Department of Biological Sciences State University of New York at Albany Albany New York USA; ^8^ Department of Biological Sciences Rensselaer Polytechnic Institute Troy New York USA; ^9^ New York Natural Heritage Program State University of New York College of Environmental Science and Forestry Albany New York USA; ^10^ New York Sea Grant Stony Brook New York USA; ^11^ New York State Energy Research and Development Authority Albany New York USA

**Keywords:** climate change, ecosystems, impacts, New York State, resilience, species, vulnerability

## Abstract

The people of New York have long benefited from the state's diversity of ecosystems, which range from coastal shorelines and wetlands to extensive forests and mountaintop alpine habitat, and from lakes and rivers to greenspaces in heavily populated urban areas. These ecosystems provide key services such as food, water, forest products, flood prevention, carbon storage, climate moderation, recreational opportunities, and other cultural services. This chapter examines how changes in climatic conditions across the state are affecting different types of ecosystems and the services they provide, and considers likely future impacts of projected climate change. The chapter emphasizes how climate change is increasing the vulnerability of ecosystems to existing stressors, such as habitat fragmentation and invasive species, and highlights opportunities for New Yorkers to adapt and build resilience.

## 1 TECHNICAL WORKGROUP KEY FINDINGS

The people of New York have long benefited from the state's diversity of ecosystems, which range from coastal shorelines and wetlands to extensive forests and mountaintop alpine habitat, and from lakes and rivers to greenspaces in heavily populated urban areas. These ecosystems provide key services such as food, water, forest products, flood prevention, carbon storage, climate moderation, recreational opportunities, and other cultural services. This chapter examines how changes in climatic conditions across the state are affecting different types of ecosystems and the services they provide, and considers likely future impacts of projected climate change. The chapter emphasizes how climate change is increasing the vulnerability of ecosystems to existing stressors, such as habitat fragmentation and invasive species, and highlights opportunities for New Yorkers to adapt and build resilience.

**Key Finding 1: Extreme climate events can have large impacts on New York State's ecosystems, and many types of extreme events are increasing in frequency and intensity as the climate changes**. Events such as intense storms, droughts, and heat waves disturb ecosystems as they harm soil, vegetation, and wildlife populations. Ecosystem management strategies focused on the impacts of extreme events will be helpful in preserving ecosystem services where achievable and can minimize the loss of future ecosystem functions.

**Key Finding 2: Rising water temperatures will have cascading effects on the composition, range, and distribution of species in New York State's waters**. Species adapted to cold water will seek more favorable habitat, species adapted to warmer water will move into previously colder habitat, and the physiological stress of warming will increase vulnerability to other stressors such as disease and invasive species. These changes are already occurring in lakes, rivers, wetlands, and marine and coastal waters. Adaptation strategies focused on identifying and maintaining coldwater habitats will benefit thermally stressed species in the coming decades.

**Key Finding 3: Human activities that degrade the environment continue to be more impactful to New York State's ecosystems than projected climate change impacts alone**. These nonclimate stressors include habitat loss and fragmentation, erosion, sedimentation, and pollution. The interaction of climate change and ongoing stressors associated with land‐use practices and land‐use change accounts for the most substantial projected ecosystem impacts. Avoiding, reducing, and mitigating nonclimate stressors is often more readily attainable than directly managing the impacts of climate change, indicating the benefit of jointly addressing climate change and nonclimate stressors in adaptation planning.

**Key Finding 4: Sea level rise will substantially alter New York State's coastal and tidal ecosystems**. Coastal ecosystems will increasingly flood, and intrusion of salt water into areas previously occupied by fresh water will cause deleterious impacts to low‐lying coastal ecosystems. The extent of coastal wetlands will decline in many areas. However, the extent of some coastal wetlands may be maintained, and other coastal ecosystems may expand, if inland habitat is available for expansion—a resilience challenge that intersects with the built environment.

**Key Finding 5: Climate change is projected to accelerate the introduction, spread, and negative impacts of invasive species in New York State's ecosystems**. New York is home to hundreds of exotic invasive plants, animals, and pathogens and has more detrimental forest pest species than any other state. Recent climate trends and rising atmospheric carbon dioxide concentrations have been identified as drivers of new and expanding infestations. Ecosystem management will require creative and coordinated measures that account for cascading feedbacks as native ecosystems become increasingly vulnerable to climate change and invasive species and lose their capacity to adapt to both threats.

BOX 1 **Developments since the 2011 ClimAID assessment**

In 2011, the ClimAID assessment evaluated risks, vulnerabilities, and potential adaptation strategies in response to ongoing and projected future climate change across New York State.^1^ The report provided an assessment of climate change impacts on eight sectors: water resources, coastal zones, ecosystems, agriculture, energy, transportation, telecommunications, and public health. The ecosystems chapter of the report focused on risks to ecosystem services and included economic valuation while highlighting vulnerabilities and potential adaptation strategies to reduce impacts from the principal climate hazards identified.^2^ The current assessment of ecosystem impacts from climate change provides an update to the previous chapter by considering information that has become available since the ClimAID assessment was published. The following are some key ways in which the current chapter differs from or advances the work provided in the 2011 ClimAID assessment:

- The 2011 ClimAID assessment applied an ecoregions approach. Ecoregions are geographic areas in which the combined effects of climate, geology, and other factors result in distinctly identifiable biological communities.^3^ The current assessment is organized differently, using broad ecosystem categories similar to those defined by the New York Natural Heritage Program.^4^
- ClimAID included a separate chapter for coastal zones that focused on both ecosystems and infrastructure.^5^ The scope of this ecosystem impacts assessment has expanded to include consideration of coastal and marine ecosystems. Coastal zone infrastructure is covered in the relevant portions of the Buildings, Energy, Transportation, and Water Resources chapters.
- The ecosystems chapter of the ClimAID assessment addressed issues of equity and environmental justice, but a notable addition in the current assessment is the consideration of Indigenous lands and Peoples.
- The current chapter highlights two issues of increased focus by the climate change science community: (1) the impacts of extreme events, particularly compound events,^6^, ^7^, ^8^ and (2) the central role of discipline integration across environmental and social sciences in managing and adapting to climate change.^9^ Both areas have seen significant advances in understanding since the ClimAID ecosystems chapter was written.

## 2 INTRODUCTION AND BACKGROUND

This chapter provides an overview of observed and projected impacts of climate change on major ecosystem types in New York State, including forests, open land, alpine ecosystems, lakes and ponds, wetlands, riverine ecosystems, and marine and coastal ecosystems. Also included are discussions of cross‐cutting ecosystem topics, such as urban ecosystems, Indigenous lands, and invasive species. The chapter builds upon the 2011 ClimAID assessment and the 2014 ClimAID supplement through analysis of recent scientific literature and incorporation of community and traditional ecological knowledge. Special emphasis is given to topics such as the Great Lakes, municipal concerns, and equity, with a focus on ecosystem services of particular importance to historically underserved or overburdened urban neighborhoods, rural townships, and Indigenous communities.

The background material in this section defines the scope of this chapter and provides context for understanding the state's ecosystems. Subsequent sections assess the state of knowledge on climate impacts and adaptation as follows:

- **Section** **3** provides an overview of observed climate impacts and an assessment of projected future climate impacts on the state's major ecosystem types.
- **Section** **4** summarizes key risks and vulnerabilities for species, ecosystems, and people, along with associated adaptation and climate resilience actions.
- **Section** **5** looks at opportunities for positive change that can grow out of climate adaptation efforts and identifies emerging topics and research needs in the ecosystems sector. This section also summarizes the major findings and recommendations presented in the chapter.
- The **Traceable Accounts** appendix examines each key finding in depth. It provides citations that support each assertion, and it presents the authors’ assessment of confidence in each finding.
- **Case studies** highlight examples of climate change impacts on a variety of ecosystems in New York State, along with adaptation and resilience strategies that could serve as models for others. These case studies are not included in the chapter proper but are available through links provided in the chapter.

### 2.1 Sector scope and context

An ecosystem is defined as “a dynamic complex of plant, animal [including human], and microorganism communities interacting with each other and the nonliving environment as a functional unit.”^10^ Ecosystem services are the many benefits people obtain from an ecosystem. These services are typically divided into provisioning (food, water); regulating (flood abatement, disease mitigation); supporting (nutrient cycling); and cultural (recreational, spiritual). The impacts of climate change on ecosystem services affects the quality of life for all people in New York State. Intact, functional ecosystems provide the foundation upon which the state's communities and economies depend.

The terms “cascade” and “cascading” are used frequently in this chapter. These terms refer to temporal sequences of unforeseen outcomes that result from actions or changes that affect a system.^11^ In this chapter, “cascade” or “cascading” primarily refers to ecosystems and climate change acting in concert with other factors, such as land‐use change, as the principal drivers. Cascading impacts result from interdependencies in systems that are coupled through positive and negative feedbacks, and these impacts can flow to other systems such as socioeconomic networks.^12^ The term cascade was first introduced to ecology through the study of trophic cascades, describing how a change at one point in a food web can have unexpected consequences to other organisms within the same food web.^13^ Cascading impacts have assumed great importance in studying climate change because of a desire to understand the full array of outcomes of a changing climate, including those that are indirectly or not obviously linked to the climate. Sometimes, a cascading impact has not been previously understood to be linked to an action or change until its occurrence motivates study that later discovers a linkage. Investigators focused on climate change are illuminating many of these cascading ecosystem impacts, which helps advance the predictive ability of ecological science.^14^ The term is applied elsewhere in this assessment to describe linkages between sector‐level impacts that can flow among socioeconomic networks,^12^ such as the effects of floods or heat waves on public health. In this chapter, the Technical Workgroup restricts its application to the ecosystem categories addressed, while recognizing that climate impacts on ecosystems typically resonate across other sectors.

#### 2.1.1 Land cover types in New York State

The state has a rich diversity of terrestrial and aquatic ecosystems.^15^ For the purposes of this chapter, the Technical Workgroup used the 2019 National Land Cover Database (NLCD) ^16^ to describe the relative amount of land within broad categories of ecosystems. Figure 5‐1 shows the distribution of the major land cover types.

**FIGURE 5‐1:**
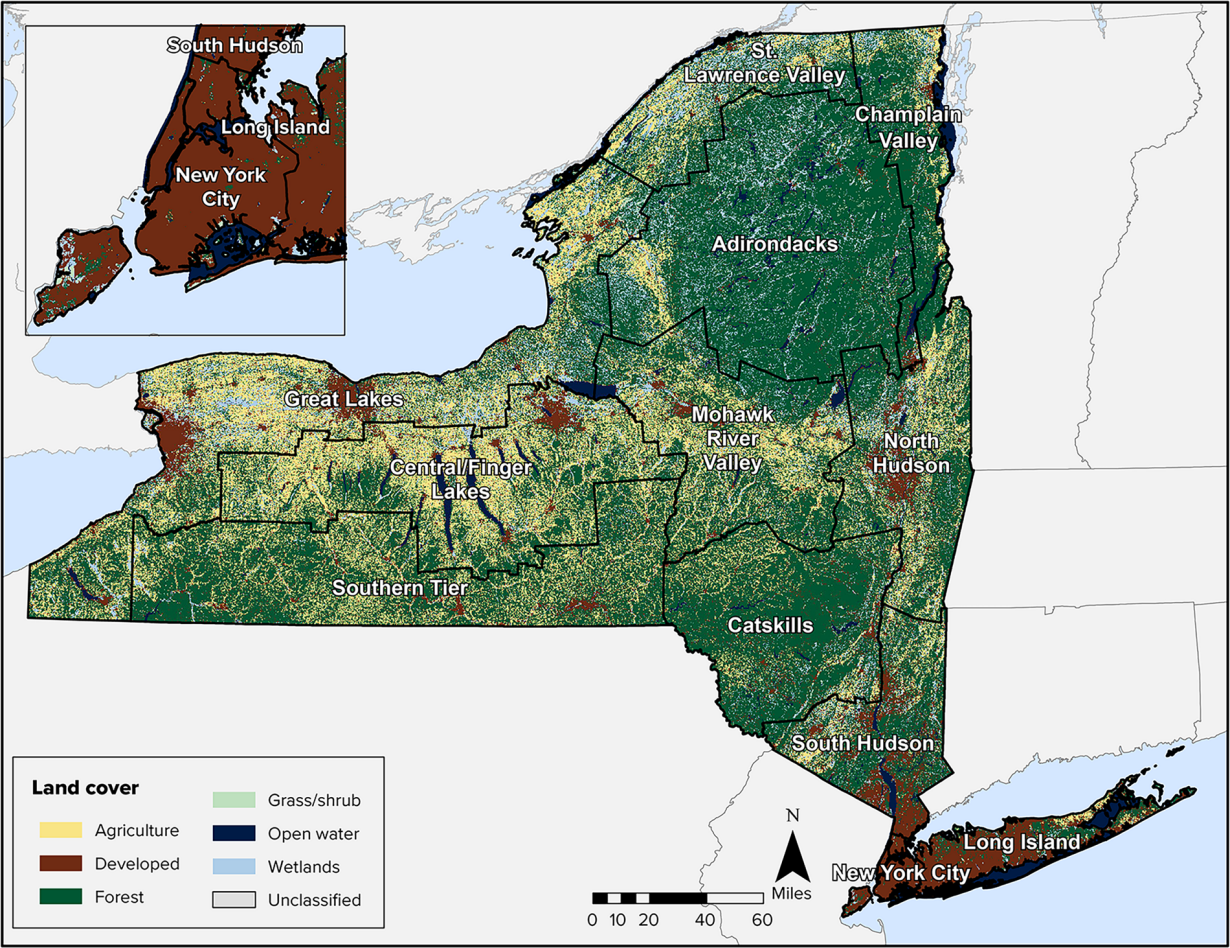
Major land cover types in New York State. Data from Dewitz and U.S. Geological Survey (2021).^16^

By land area, New York State is primarily rural, consisting of 75% forest and agricultural lands and 11% developed land (Figure 5‐2 and Table 5‐1). Considering patterns by assessment region (Assessment Introduction), developed land is dominant in New York City and Long Island but does not exceed 25% of land cover in any other region. The central part of the state includes the largest relative amount of agricultural land.

**FIGURE 5‐2:**
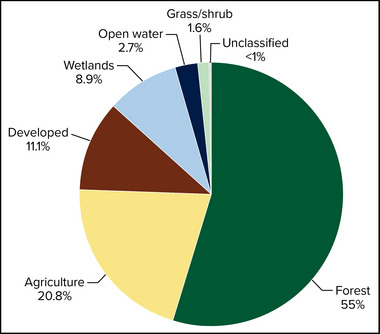
Percent land cover in New York State. Data from Dewitz and U.S. Geological Survey (2021).^16^

**TABLE 5‐1:** Percent land cover for New York State, its 12 climate assessment regions, and Indigenous lands.

Land cover type	New York State	Indigenous lands^a^	Adirondacks	Catskills	Central/Finger Lakes	Champlain Valley	Great Lakes	Long Island	Mohawk River Valley	New York City	North Hudson	South Hudson	Southern Tier	St. Lawrence Valley
Open water	2.7	7.4	3.8	2.1	4.0	3.6	2.0	1.4	2.6	0.8	2.1	4.8	0.9	2.0
Developed	11.1	8.2	2.2	7.1	10.1	6.5	14.0	64.0	7.7	89.2	12.9	25.1	7.7	5.5
Grass/shrub	1.6	1.3	1.9	1.1	1.4	2.4	1.8	3.1	1.6	2.2	1.8	1.4	1.0	2.2
Forest	55.0	53.8	77.9	76.6	36.9	67.6	34.1	21.3	53.9	4.0	55.2	49.7	64.2	46.6
Agriculture	20.8	10.6	2.8	9.1	41.1	10.6	34.4	4.7	25.9	0.2	19.3	10.9	23.8	22.5
Wetland	8.9	18.7	11.4	4.1	6.4	9.3	13.6	5.5	8.4	3.6	8.8	8.1	2.5	21.2

*Note*: This table provides a land cover analysis for each of the 12 climate assessment regions. Data in the table are adapted from the 2019 NLCD. For the purposes of this broad overview, some land cover categories in the database were combined as follows: open water (NLCD category 11, which includes rivers as well), developed (categories 21 + 22 + 23 + 24), grass/shrub (categories 31 + 52 + 71), forest (categories 41 + 42 + 43), agriculture (categories 81 + 82), and wetland (categories 90 + 95). The data do not include the surface area of the Great Lakes and the marine and coastal district waters that lie within New York State. Table adapted from Dewitz and U.S Geological Survey (2021).^16^

^a^ Land cover data were compiled for 10 distinct areas of Indigenous land, including lands under the jurisdiction of eight federally recognized Indigenous Nations in New York State, as well as the Poospatuck Reservation of the Unkechaug Band, which is recognized by the state.

Forest is the dominant land cover (55%) across New York State (Figure 5‐2). The Adirondack and Catskill regions each have more than 75% forested land, and the Champlain Valley and Southern Tier each have about 65%. Other than New York City and Long Island, all regions have more than 30% (Table 5‐1) forest land cover, even the heavily agricultural Central/Finger Lakes region.

A total of 13.6% of New York State is covered by water, including inland waters, the Great Lakes, and coastal waters.^17^ New York is the only state with both Great Lakes shorelines (on Lake Erie and Lake Ontario) and marine shorelines. Inland surface waters include the 11 Finger Lakes; Oneida Lake; Lake Champlain; and several river basins, including the Genesee, Hudson, St. Lawrence, Delaware, and Susquehanna. These water bodies provide widespread diversity and abundance of aquatic habitats. The St. Lawrence Valley and the Great Lakes regions have the greatest relative wetland area, with 21.2% and 13.6%, respectively. Wetlands and open waters are especially prevalent on Indigenous lands located in New York (Table 5‐1).

Agricultural land is present throughout the state but is highly variable among the regions. For example, the Central/Finger Lakes region includes 41.1% agricultural land, and the Mohawk River Valley, Southern Tier, Great Lakes, and St. Lawrence Valley each have more than 20% agricultural land. In contrast, New York City, the Adirondacks, and Long Island each have less than 5% agricultural land.

It is important to note that more than 85% of New Yorkers live in the New York City metropolitan area or in the metropolitan areas of Albany, Syracuse, Rochester, and Buffalo.^18^ The impacts of climate change on urban ecosystems are a topic of great interest in New York City and throughout the state and are considered explicitly in this assessment.^19^

### 2.2 Key climate hazards

Climate is a fundamental driver of the distribution and function of ecosystems and the seasonality and habitat range of individual species. A changing climate is measurably altering New York State's ecosystems. Several climate hazards, including sea level rise, temperature change, changes in precipitation amount and intensity, and extreme events, are relevant to understanding ecosystem effects. Other climate‐related metrics—such as water temperature, growing season length, winter climate, evapotranspiration, and climate velocity (the rate at which a species would need to migrate to remain in a stable environment as climate conditions change)—are considered important for understanding patterns of temporal change. Table 5‐2 provides an overview of the direction and magnitude of change and the types of impacts for these stressors. This table largely describes long‐term, multidecadal temporal patterns of change. Additionally, many changes in climate extremes have been described at global, regional, and local scales that are relevant to New York State, but are not represented in this table, such as increases in the duration and intensity of heat waves,^20^, ^21^, ^22^ decreases in the intensity and duration of cold waves,^22^ and increases in the frequency and intensity of large precipitation events.^23^ Many of these extreme climate phenomena such as droughts and storms have inherent natural variation that can depend on interannual and interdecadal climate oscillations that may be unrelated to long‐term climate change.^24^, ^25^ One or more extreme climate events that occur over a short time interval are termed compound events, and the ecosystem impacts of these sequential events are an area of emerging research interest across the globe.^6^, ^26^ Chapter 2, New York State's Changing Climate, provides additional data on observed and projected changes in these climate variables in general, while Section 3.1 provides additional detail on climate changes that are particularly relevant to New York's ecosystems.

**TABLE 5‐2:** Trends in several climate metrics described in the scientific literature and examples of potential impacts on ecosystems in New York State.

Climate metric	Direction and magnitude of observed change	Examples of potential impacts
Water temperature: Rivers	+0.4°F to +0.8°F per decade^27^, ^28^, ^29^	Decreased population size of coldwater species^30^ Loss of brook trout habitat^31^
Water temperature: Lakes	+0.4°F to +2°F per decade^32^, ^33^, ^34^	Lengthening aquatic growing season^33^ Increases in duration and intensification of stratification^35^
Water temperature: Marine	+0.3°F to +0.6°F per decade^29^, ^36^	Stress on coolwater species^36^ Lengthening growing season^37^
Growing season length	+0.7 to +6 days per decade^38^, ^39^, ^40^	Increased gross productivity ^38^ Increased evapotranspiration^38^, ^41^ Increased warm season drought risk^38^, ^41^ Pollinator asynchrony^42^
Start of growing season	−1 to −4 days per decade^38^, ^39^	Earlier budbreak^38^ Increased risk of false spring^43^ Increased freezing risk^44^
End of growing season	+1 to +5 days per decade^38^, ^39^	Delayed senescence^38^, ^41^, ^45^
Winter conditions	−1.8 frost days per decade, −2.1 snow‐covered days per decade^46^	Promotion of invasive and native insect pests^46^ Increased risk of forest canopy damage^46^, ^47^
Evapotranspiration	No change to +0.11 inches per decade^48^, ^49^	Cooling benefit in urban ecosystems Drying of soils after floods Intensification of drought^50^
Climate velocity	Generally north, 0.26 miles per year^51^	Range moves north^51^ Species migration^51^

*Note*: The sources used for this table reflect data or studies that encompass New York State or adjacent areas and thus are expected to reflect patterns in the state.

### 2.3 Nonclimate factors

Climate change interacts (in some cases synergistically) with nonclimate factors and stressors such as land use, land and water management (e.g., Great Lakes water levels), and air and water pollutants. These interactions influence plant and animal species, ecosystem structure and function, and, thus, ecosystem services. It may be easier to manage nonclimate stressors than to directly manage climate impacts. While it may be challenging to fully differentiate climate impacts from those caused by nonclimate drivers, it is critical to consider the ecological dynamic of nonclimate stressors and climate‐related impacts when assessing projected climate impacts in New York State, along with key vulnerabilities, opportunities, and adaptation strategies.

#### 2.3.1 Land use

Nonclimate land‐use stressors such as land development, energy and utility infrastructure, and agricultural practices can result in a variety of impacts, including deforestation; habitat loss, degradation, and fragmentation; air and water pollution; and the spread of invasive and nuisance species, pathogens, and pests. Such land uses continue to impose substantial pressure on natural systems.

Land use, more so than climate change, currently dictates the function and services provided by New York State's ecosystems. The relationship between land‐use stressors and climate change is a crucial consideration in developing climate resilience. In many instances, management of land use is more effective and more attainable than managing direct climate change impacts. Reducing the impact of land‐use stressors could make it possible for plants and animals in an ecosystem to better withstand the effects of a changing climate.

Land‐use patterns have produced an extensively fragmented and disrupted landscape due to piecemeal deforestation, agriculture, wetland loss, and dense road networks.^19^, ^52^, ^53^, ^54^ Fragmentation and loss of habitat account for the vast majority of impacts to wildlife and biodiversity globally.^55^ More than 20% of the state's land base is used for agriculture. Many agricultural practices have decreased the connectivity and quality of wetland, forested, and grassland ecosystems through filling, draining, deforestation, tilling, and pesticide and fertilizer applications.^56^ These changes have downstream impacts on water quality and coastal habitats as well.^57^, ^58^ Past and current land use involving natural resource harvesting and management in semi‐natural ecosystems (e.g., working forests) may also present challenges in observing and predicting climate impacts. For example, past logging of managed forests can homogenize tree communities and mask ongoing ecosystem responses to a changing climate, causing delays in measurable ecosystem trends.^59^, ^60^

Climate change and land use interact in complex ways and the most recent Intergovernmental Panel on Climate Change assessment ^61^ dedicated an entire chapter to this topic. For example, natural land cover has a mitigating effect on climate change by absorbing atmospheric carbon dioxide.^62^ Disturbance and habitat loss diminishes this effect. Some land uses, most notably agriculture and development, are a major net source of greenhouse gas emissions. Climate change can act to amplify the deleterious effects of fragmentation and habitat loss, limiting the capacity for ecosystems and natural populations to migrate in response to habitat loss from temperature change.^63^ For example, current infrastructure encroachment on floodplains limits the ability of shoreline ecosystems to migrate inland in response to flooding and sea level rise. Climate change and land‐use change also act synergistically to increase the potential for invasive species colonization. For example, urban development promotes the spread of invasive species into floodplain forests.^64^ Increased disturbance in these same areas due to intense storm events further promotes the spread of invasive species.

Potential drivers of future land‐use change in New York State include the construction of large‐ and small‐scale energy generation facilities and expansion of the electrical grid. These types of infrastructure projects can encroach into sensitive natural lands, such as ridgelines, forests, and meadows, resulting in greater habitat fragmentation.^65^ Agricultural practices continue to shift cultivation in ways that can disrupt native ecosystems, including continued removal of hedgerows and drainage of ephemeral wetlands and increased row cropping to support concentrated animal feeding operations (CAFOs). Climate migration (human) and expansion of residential and industrial footprints to higher elevations and latitudes may become increasingly important sources of ecosystem impacts in the future, although motivating factors for human migration are complex and are currently influenced more by socioeconomic drivers than climate drivers.^66^, ^67^

#### 2.3.2 Invasive species: Nuisance species, pathogens, and pests

Threats from invasive species (e.g., water chestnut, hemlock woolly adelgid) and nuisance species (e.g., white‐tailed deer, resident Canada geese) are substantial and growing. Careful, informed prevention and management is key to achieving cost‐effective threat reduction. Compounding effects of climate change add further urgency to these challenges.

Other nonclimate stressors that affect New York State's ecosystems and ecosystem services include invasive species, a term that includes nuisance species, pathogens (disease‐causing microorganisms), and pests (mainly insects). The state has been particularly susceptible to biotic exchange—the movement of exotic invasive species into the state—carried by global commerce and many waves of international travelers passing through its numerous ports of entry.^68^ Although New York State only defines a fraction of these species as “invasive” (causing economic harm, environmental damage, or harm to human health), the sheer number of species involved is large, and some pose serious challenges. Section 3.9.4 explores this topic in depth.

### 2.4 Equity and climate justice

The impacts of climate change on ecosystems and their beneficial services raise several environmental justice and equity issues, as described below and throughout this chapter. People in New York State will experience climate change differently, depending on myriad factors that make some regions, communities, groups, and individuals more sensitive to harm from climate change and less able to cope and respond. Examples of these differential impacts include the following:

- Coastal communities will experience impacts from sea level rise, including flooding, storm surge, and saltwater intrusion, as well as land loss due to erosion.^69^
- Rural communities will face impacts that affect their natural resource‐dependent economies.
- As described in the Human Health and Safety chapter, urban communities will experience intense impacts from extreme heat due to the urban heat island effect.^70^ Some communities will benefit from the regulating service of tree cover, which can provide cooling in areas with parks and greenspace.^71^ However, parks and greenspace are unevenly distributed in cities.

Diverse communities of all ethnicities, rural and urban, use nature for foraging, fishing, and hunting to provide food security.^72^, ^73^ Some communities also harvest materials from nature for cultural activities (such as basket making) and health care needs (such as herbal medicines). This direct relationship with ecosystems increases vulnerability to ecosystem impacts. For example, immigrant communities are disproportionally impacted by the consumption of contaminated fish due to cultural traditions, economics, and language barriers.^74^ Any impacts to species consumed for food will be felt by communities that engage in subsistence activities, many of which are already experiencing environmental and socioeconomic injustices.

Rural communities depend on agriculture, silviculture, ecotourism, and other natural resource‐based economies that can be disproportionally affected by climate impacts. Soil erosion is a major threat to rural residents, farmers, and townships primarily due to loss of farmland soils, damage to infrastructure, and impacts to water quality. For drinking water, rural communities often rely on residential wells and/or bodies of surface water, which are vulnerable to precipitation changes and extreme storm events. In addition, most of New York's land base lies within small, rural municipalities, which often have limited resources to manage land for climate resilience. Only 12% of the state's Climate Smart Communities are rural,^75^ suggesting that many rural communities may lack the awareness, capacity, and/or consensus required to take adaptive action. The capacity limitations and institutional structural barriers facing rural municipalities make it difficult for them to effectively address the cross‐cutting issue of climate change.

### 2.5 Indigenous communities

There are 10 territories held by federally or state recognized Tribal Nations located within New York State, totaling approximately 137 square miles (87,650 acres).^76^ Indigenous communities within these lands and elsewhere rely on diverse ecosystems and the natural resources and ecosystem services they provide, including many plant and animal species valued as traditional foods, as medicine, and for spiritual significance.^77^, ^78^ Indigenous communities have long dealt with major environmental justice issues. Degradation and loss of forest, freshwater, and riparian ecosystems continue to disproportionately impact the ability of Indigenous Peoples to benefit from ecosystem services through activities such as hunting, fishing, and plant harvesting. Climate‐induced changes in precipitation and temperature will result in direct and indirect impacts on ecological processes in areas already experiencing environmental justice concerns. Section 3.8.2 discusses climate impacts and issues relevant to Indigenous communities, including the exacerbating impacts of climate change on existing environmental justice issues, as well as impacts on culturally significant flora and fauna. Other sections also assess the impacts of climate change on ecosystems found within Indigenous lands.

### 2.6 Opportunities for positive change

A changing climate is resulting in notable challenges for most ecosystems in New York. However, climate change may also yield some impacts, outcomes, and opportunities that could be considered positive from some perspectives. Impacts of climate change may result in an increase in area, function, or restoration opportunities for some ecosystem types and species. For example, warming water temperatures are causing a northward expansion of the range of the blue crab and increasing the survivorship of this species in New York State. (For more information, refer to the Shifts in Lobster and Crab Populations case study.) The work of adapting to climate change also creates opportunities for positive change, especially when resource managers use best management practices to build ecosystem resilience. Such practices can lead to measurable improvements in ecosystem health and enhanced ecosystem services. In some settings, adaptation may present opportunities for contributing to equitable transitions. For example, investing in new green infrastructure and nature‐based solutions in urban ecosystems can help to address historic inequities in the distribution of parks and street trees, which are disproportionately found in more affluent neighborhoods. Section 5.1 provides a more detailed summary of opportunities for positive change associated with building more resilient ecosystems.

## 3 ECOSYSTEMS AND IMPACTS

BOX 2 **Longer growing seasons**

Ongoing and projected changes in the growing season could profoundly affect ecosystem processes, including:

- Forest productivity
- Migration patterns
- Invasive species introductions
- Carbon sequestration

Climate change has resulted in measurable impacts to New York State's ecosystems. Observed impacts are most often the result of climate hazards (e.g., increasing temperature, changing precipitation patterns, increasing frequency of extreme storm events) acting in concert with existing nonclimate stressors such as agriculture, development, and other land use patterns.^7^, ^79^ These impacts can alter ecosystem processes and the services that ecosystems provide to people. Increases in the seasonality, frequency, and intensity of extremes can have a more severe impact than changes in average conditions and incur greater societal impacts and costs. While the effects of specific climate hazards on individual ecosystem properties can be substantial (e.g., extreme storm event impacts on headwater riparian wetlands), focusing on a single effect, species, ecosystem, or ecosystem service can result in overlooking some of the most important climate impacts and vulnerabilities. Interactions of climate and nonclimate stressors and the cascading or cumulative changes that affect ecosystem processes pose the greatest risk in terms of magnitude of impact.^80^ These interactive and cumulative effects are also the most difficult to predict.

Section 3.1 describes trends in four major climate variables that affect ecosystems in the state. The subsequent sections provide an overview of observed climate impacts and assess projected future climate impacts for major ecosystem types in New York State, including forests, open land, alpine ecosystems, lakes and ponds, wetlands, riverine ecosystems, and marine and coastal ecosystems. Also included are discussions of cross‐cutting ecosystem topics and compounding factors.

### 3.1 Changes in climate variables relevant to many ecosystem types

#### 3.1.1 Water temperature

The temperatures of lakes, rivers, estuaries, and oceans are broadly increasing across the globe, particularly during recent decades,^27^, ^81^, ^82^ and warming is expected to advance further through the 21st century. Warming of surface waters has numerous implications for aquatic ecosystems, including changes in the duration of ice cover, dissolved oxygen concentrations, aquatic metabolism, and availability of optimal thermal habitat for many species. New York State has experienced a clear pattern of climate‐related warming of rivers, lakes (including the Great Lakes), and estuarine waters, despite complicating factors related to human activities such as land‐use change and damming of rivers. Warming rates are in the range of slightly less than 0.5°F per decade up to 2°F per decade in some locations (Table 5‐2). Warming air temperatures are generally the principal driver of warming waters. Recent investigations have highlighted an increase in the intensity and duration of aquatic heat waves, periods with much higher than normal temperatures in lakes^83^ and the oceans.^84^, ^85^ These extreme events are expected to further intensify with continued climate change in the 21st century^83^ and can have a broad range of effects on ecosystems.

#### 3.1.2 Growing season and winter climate

The growing season is the period when biological activity is greatest in ecosystems, typically from spring through fall. For terrestrial ecosystems, the growing season can be defined as the period from the last frost of spring to the first frost of the fall.^86^ The length of the growing season has generally increased in recent decades at all scales—globally, throughout the northern hemisphere, across the United States, in the Northeast, and in New York State.^38^, ^87^, ^88^ For example, one study of watersheds in the Catskills region found (with one exception) that the growing season length increased by 2.6−7.5 days per decade from 1960 to 2000, a trend driven almost equally by an earlier occurrence of last spring frost (2.6−4.3 days per decade) and a later occurrence of first fall frost (2.7−3.2 days per decade).^38^ The same study projected even greater changes in the Catskills during the 21st century, with the spring growing season moving earlier at an estimated rate of 4−11 days per decade by mid‐21st century and at an estimated rate of 4.5−15 days/decade by late 21st century across a range of climate models and emissions scenarios. These changes, when combined with an expected delay of the first fall frost, result in a projected expansion of the growing season by an estimated 10−25 days per decade by the mid‐21st century and by an estimated 13−40 days per decade by the late 21st century.^38^

Growing season timing and duration have a strong impact on phenology, which is the seasonal timing of biological activities as they occur in ecosystems. Ongoing and projected changes in the growing season may profoundly affect the timing of events such as the flowering of plant species, the arrival of migrating bird and fish species to a given location, and leaf fall. A phenomenon of growing research interest and concern is the increasing risk of false spring, wherein an early period of warmth initiates budbreak but is then followed by a period of colder temperatures that may have long‐term implications for ecosystem health.^89^ Growing season changes can also affect a variety of ecosystem processes and functions, such as forest productivity (the rate at which biomass is generated),^90^ the movement of invasive species,^91^ and the capacity of ecosystems to sequester carbon.^7^ Winter climate also affects the phenology of many species^92^ and can lead to changes in canopy structure, nutrient cycling, and fine root health.^46^

#### 3.1.3 Evapotranspiration

Evapotranspiration, dominated by the flow of water through roots into leaves and to the atmosphere, is a fundamental component of regional and global water and energy budgets and is closely linked to many aspects of ecosystem typology, function, and process. Climatic variables such as air temperature, precipitation, wind speed, and incident solar radiation are important drivers of evapotranspiration, primarily through their impacts on terrestrial vegetation in temperate regions. For example, in New York State, transpiration (largely by trees) exceeds vaporization of intercepted rainfall from vegetation and bare soil by more than two‐fold.^93^ Examination of broad temporal patterns of annual evapotranspiration across the United States indicates that rates are generally increasing.^94^ Increasing atmospheric carbon dioxide concentrations and decreasing concentrations of air pollutants such as ozone result in increased water use efficiency by vegetation and act as a negative feedback, which limits the rate at which evapotranspiration increases.^95^, ^96^ Increased evapotranspiration can affect ecosystems in a variety of ways, including reducing groundwater recharge and surface water availability and exacerbating drought effects on vegetation, such as reduced plant growth and regeneration and increased chance of wildfire.^97^, ^98^

#### 3.1.4 Climate velocity

Climate velocity (Figure 5‐3) is a metric that quantifies the rate at which a species would need to migrate to remain within a suitable habitat as climate conditions change.^51^, ^99^, ^100^, ^101^ This metric is expressed as a speed and a direction (e.g., 0.26 miles per year, north). Climate velocity typically refers to the rate at which a species must move to stay within a suitable *thermal* environment, but the metric can also be calculated for variables other than temperature, such as precipitation, wind, and humidity, or for several variables simultaneously in a multivariate approach, which can result in complicated spatial patterns.^102^ To maintain preferred temperatures in the face of warming, many species are moving to higher elevations,^103^, ^104^, ^105^ farther toward the poles,^106^, ^107^, ^108^, ^109^ or deeper in the water column.^100^, ^107^

**FIGURE 5‐3:**
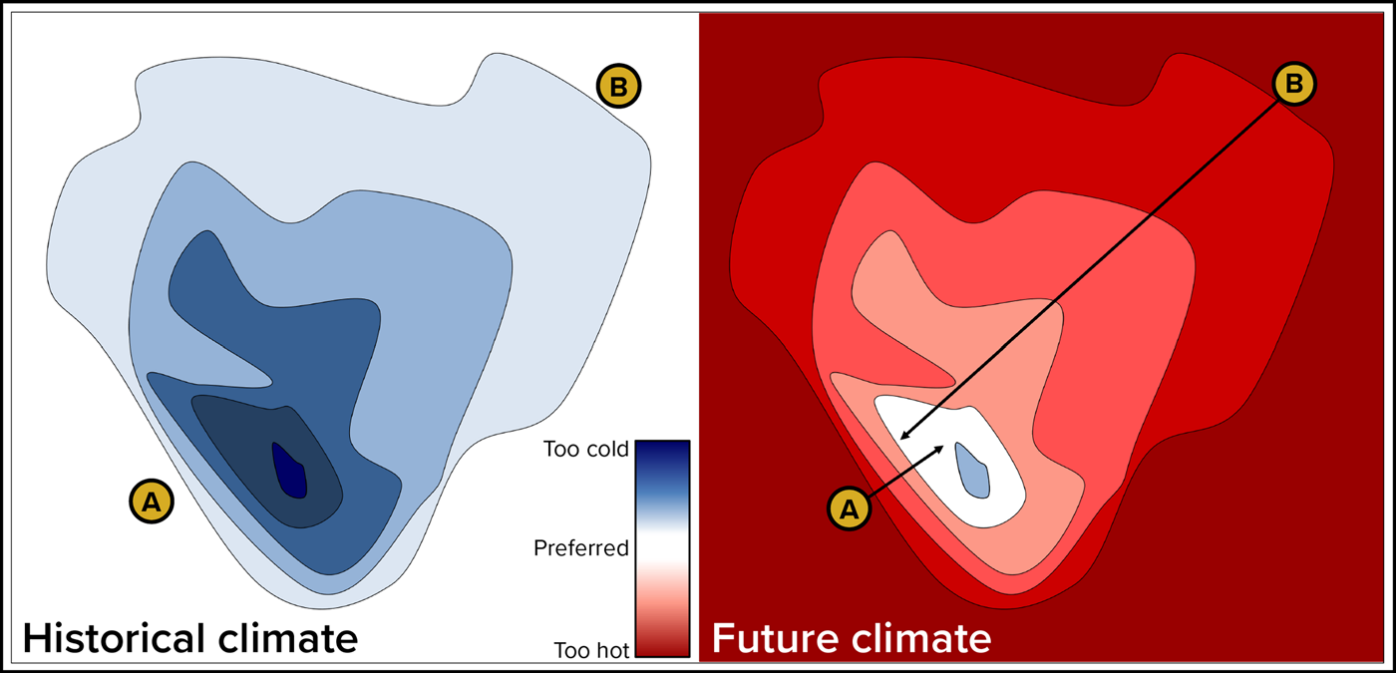
Climate velocity. Climate velocity accounts for both warming rate and spatial gradient in temperature. The plots here show a hypothetical topographic map of a mountain with two species (A and B). In the historic climate, both species have preferred temperatures at the base of the mountain. In the hypothetical future climate, both species must move up in elevation to maintain their preferred temperature, with species B having to move a greater distance due to a shallower spatial gradient in temperature. Because of this, the climate velocity for species B is greater than the climate velocity for species A, and velocities are also faster for both species under faster warming rates.^110^ Figure adapted from an illustration by Kevin C. Rose.

Climate velocity is a useful concept for climate‐related ecosystem management and adaptation planning because of its applicability to shifts in habitat range and potential migration patterns for a wide variety of terrestrial and aquatic species.^107^, ^111^ In the face of ongoing climate changes, comparing estimated climate velocities with dispersal rates can elucidate challenges to species^101^, ^112^, ^113^ and predict extirpations or locations of new refugia, which are “safe havens” where species can remain relatively buffered from climate‐related stressors.^101^ In fact, climate velocities have already proven effective in explaining and predicting large‐scale range shifts in marine, terrestrial, and river species.^100^, ^101^, ^113^, ^114^, ^115^, ^116^, ^117^ For example, climate velocity gradients match scientists’ expectations of massive displacement of hundreds of marine taxa.^100^ Additionally, heterogeneity in historical climate velocities created refugia that match today's biodiversity hotspots.^114^ Section 3.9 applies the concept of climate velocity to discuss projected impacts of climate change on specific species in New York State, while Section 4.7 explains how this concept can inform adaptation measures.

### 3.2 Forests

BOX 3 **Takeaways**

- In the absence of increased disturbance from wildfire, intensification of storms, and pest/pathogen outbreaks, mature trees in most New York State forests are likely to show little change by the end of the 21st century. However, growth rates of trees near the southern or lower elevation limit of their range will likely slow in the coming decades, a change that may already be occurring. Furthermore, seedlings may already be showing northward or elevational migration, especially at the deciduous−boreal boundary (ecotone), but this progression likely lags climate change and is confounded by disturbance factors and high spatial variation.
- Climate‐related stressors could diminish the potential for enhanced forest productivity that may result from warmer temperatures and longer growing seasons. Examples of such stressors include increases in invasive pest species and changes that impede regeneration, such as seedling loss to summer drying, competition from more abundant invasive plants, and increased deer browse.
- Many regionally important forests will be subject to more direct climate hazards originating from sea level rise; changing inland hydrological regimes; and warmer, drier summers.
- In the near term, forests will benefit from adaptive management practices such as promoting overall resilience, facilitating migration, and transitioning stands to new species compositions. Adaptive management could help address regeneration challenges, climatic stress, and slowing growth rates of resident species as these issues reach an increasing state of disequilibria with the changed climate. Such measures will require a coordinated effort made challenging by the dominance of privately held forest land in the state.

#### 3.2.1 Description and importance

New York State's forests cover nearly 19 million acres,^118^ accounting for 55% (Figure 5‐1) of the total land area in the state. These forests are valued for the wide array of ecosystem services they provide, including recreation, wildlife habitat, timber production, carbon storage, clean water and air, and aesthetic beauty.^119^ Since the mid‐19th century, forests in the state have been recovering from two large‐scale disturbances: (1) clearing of land and establishment of agriculture and (2) widespread logging.^120^ Following almost a century of forest area expansion, the rate of expansion slowed in the 1990s, and the total extent of forest cover peaked in the early 21st century before declining slightly from 19.0 to 18.7 million acres between 2012 and 2017.^118^ Conversion of forest to agriculture was the dominant driver of the decline in forest cover during this period; conversion to developed land was the second leading cause of the decline in forest cover.^118^ Forested area that is categorized as lying within the wildland−urban interface increased by about 1.7 million acres from 1990 to 2010.^118^ Forested area lying within this interface accounts for 33% of the total forested land in the state. While forests at this interface provide important ecological services to communities, including support for air and water quality and scenic and recreational resources, there are also increased risks to human life from wildfire, as well as risks of invasive species and pests, fragmentation and loss of connectivity among native species, and wildlife mortality.^118^

Forests are found throughout New York State (refer to Figure 5‐4), and the diversity of geology, soils, climatic conditions, and land‐use histories across the state's regions has resulted in a diversity of forest ecosystem types. The state has 68 distinct forested ecological communities (28 upland, 19 subalpine woodland/barren, 10 wetland, 7 peatland, and 4 cultural)^4^ and more than 100 native tree species.^121^ The wide variation in forest communities results in wide variation in the risks from climate change, as discussed in Section 3.2.3. Although there are numerous forest types, many forests are heavily dominated by maple‐beech‐birch (55% of the state's forested acreage) (Figure 5‐5) and oak‐hickory (17%). About 62% of private forests and about 59% of public forests were between 41 and 80 years old in 2017, highlighting a fairly uniform forest age structure (successional stage) for much of the state.^118^

**FIGURE 5‐4:**
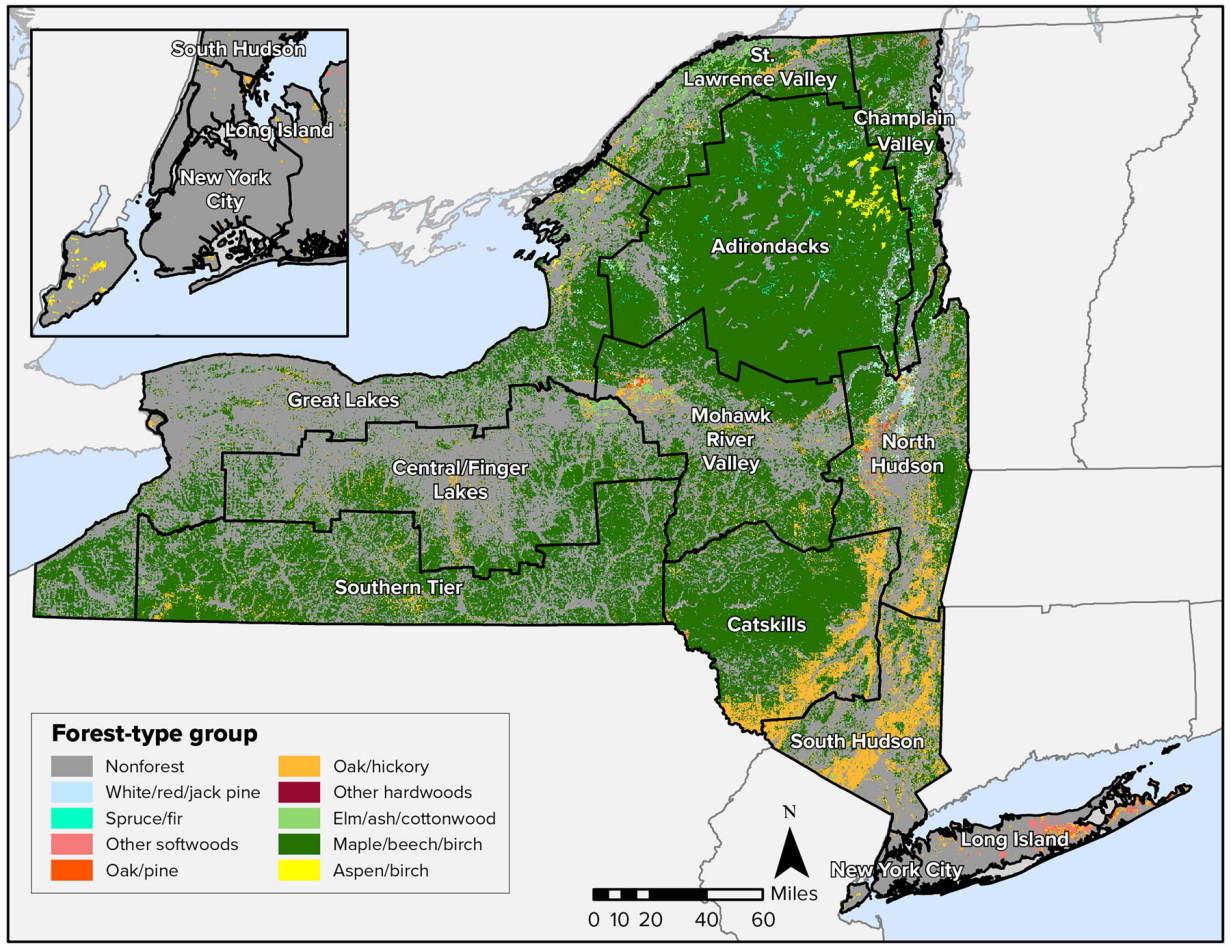
Major forest‐type groups of New York State. Data from Ruefenacht et al.^122^

**FIGURE 5‐5:**
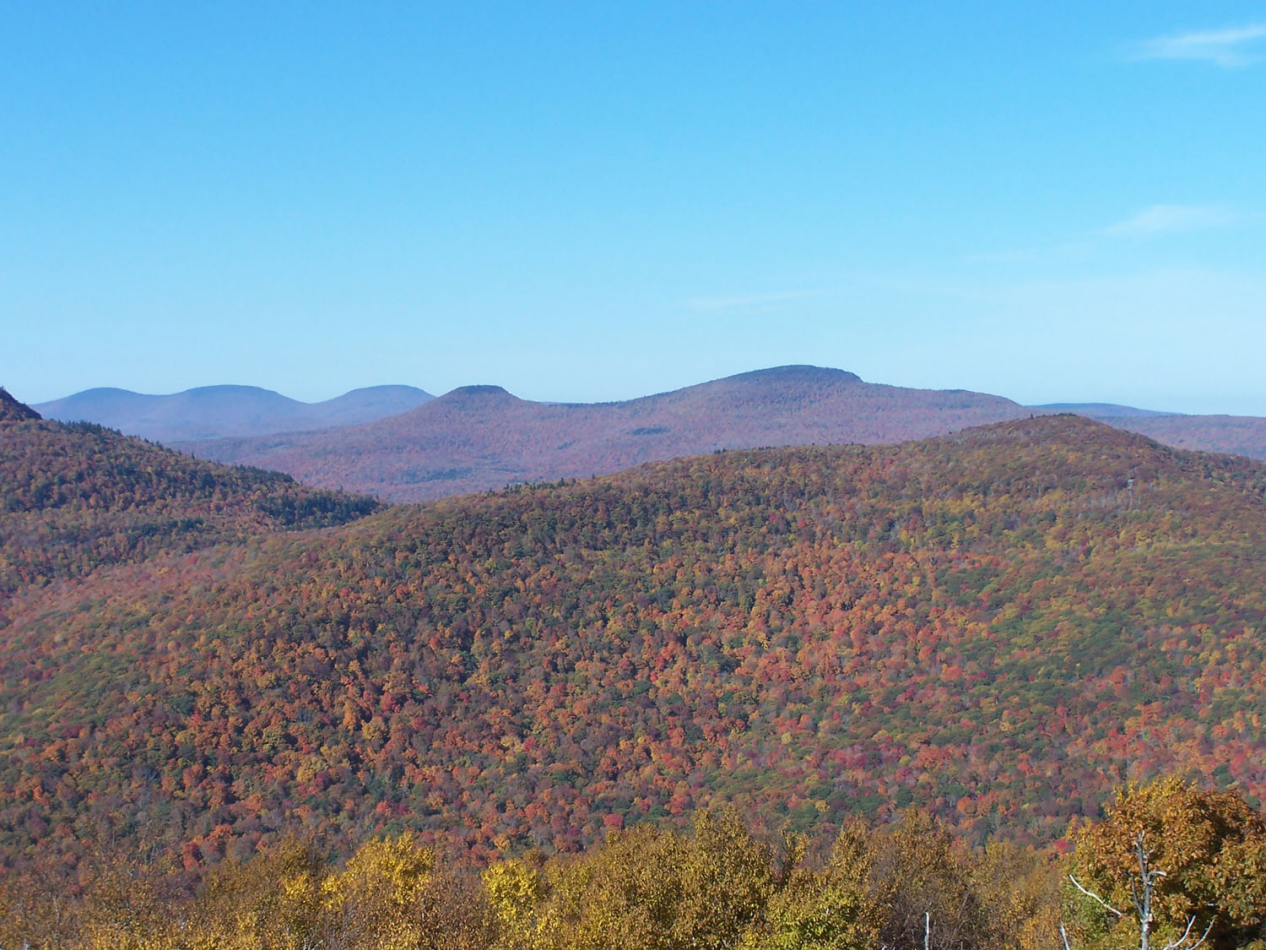
A maple‐beech‐birch forest in the northern Catskill Mountains. This forest type is the most dominant in New York State. Photo by Doug Burns.

Across regions and ownership types, the trees in New York's forests are the oldest and largest they have been since modern, systematic forest inventories began in the 1950s.^118^ The growth of forest trees, as reflected by measures of wood volume and aboveground biomass, continues to outpace losses from mortality or removal.^118^ The volume of wood that can be cut to make lumber continues to increase statewide. In 2019, the most recent year for which data are available, the growth of forest trees exceeded the rate of harvest by a ratio of more than 4:1.^123^ Despite continued growth, clear risks have been identified, including increasing forested area at the wildland−urban interface and associated human−environment conflicts, decreases in some measures of biodiversity, and diminished young forest habitat reflecting challenges to regeneration from browsing.^118^

Forest ecosystems in New York provide critical support for biodiversity, including habitat for many state‐listed and federally listed species. Forests store carbon, cycle nutrients, protect soils from erosion, and purify air and water. Protection of extensive forested area in the Catskill Mountains to maintain the quality of New York City's water supply was recently highlighted in a report by the World Bank as an example of the economic value of the ecosystem services forests provide.^124^ The economic and cultural importance of New York's forests is hard to overstate. More than 10 million acres of the state's forests (56%) are classified as family forests, owned by individuals, families, or small organizations like summer camps and hunting clubs.^125^ Working in the woods—either professionally or just on weekends to put up firewood, make maple syrup, or bring in meat—is part of the heritage of many New York families. Hundreds of thousands of people are drawn each year to the forests for recreation and retreat. Many Indigenous traditions center on the sustainable use of forest flora and fauna through cultivation and by expressing gratitude for the natural world. Forests are especially important to rural economies, and the sustainability of the state's forest products industry depends on the resilience and productivity of New York's rural forest ecosystems.

#### 3.2.2 Impacts and risks

New York State's forests are subject to the changes in climate patterns and physical processes discussed in New York State's Changing Climate and in Sections 2.2 and 3.1 of this chapter. Particularly relevant are the expected increases in temperature, the amount and variability of precipitation, and the frequency and intensity of extreme weather events.^2^, ^126^, ^127^ The subsections that follow discuss the potential impacts of these changes, with a particular focus on trees and their projected responses. Climate impacts on other components of forest ecosystems, such as wildlife and rare or endangered plants (Section 3.9.3), are addressed in more detail in other sections of this chapter. Impacts on coastal forests from inundation, erosion, and saltwater intrusion^128^ are addressed in more detail in Section 3.8.2.

##### 3.2.2.1 Shifts in plant species composition

The 2011 ClimAID assessment reported that, within this century, New York State's forests were expected to “disassemble and reassemble into new forest types that have combinations of species different than those today.”^2^ These expectations were informed by studies that related the observed geographic distribution of tree species to current climatic variables and then projected future species distributions as a direct function of modeled future climate projections.^129^ This approach led, for example, to the conclusion that most Adirondack boreal species would not survive a 5°F temperature rise.^130^, ^131^ However, in an important development since publication of the ClimAID report (2011), observations and ecological modeling simulations have suggested that little change in the mix of dominant tree species is so far evident,^132^, ^133^ and little shift in the presence of these dominant species where currently located is expected through the end of this century.^134^, ^135^, ^136^, ^137^ Despite the apparent stasis of mature stands in New York forests, there is evidence of northward migration of deciduous species into boreal habitat in nearby Quebec based on resampling of forest plots.^138^ Migration has thus far been limited (1.9−3.1 miles [3−4 kilometers] per decade for red maple and sugar maple and less for other deciduous species), highly variable, governed in part by disturbance, and mainly evident in saplings and not mature trees.^138^, ^139^ Many tree species have increased their tendency for migration in response to increasing precipitation in recent decades rather than rising temperature, as observed by a westward migration of northern hardwood species into previous central hardwood regions in Western New York.^140^ Evidence from plot warming experiments confirms that reductions in net photosynthesis and growth rate are evident in species growing near the northern limit of their range along the temperate−boreal ecotone.^141^

The lags in climatic response are the result of myriad factors, including seed dispersal limitations, soil properties, and interspecies competition for light.^142^, ^143^, ^144^ Although broad northward and upslope migration of the dominant tree species is highly likely to occur and may already be occurring to a limited extent in New York State, larger changes in species dominance among mature trees are expected to occur one to two centuries in the future. The timing will be dependent in part on future emissions scenarios and the magnitude of resulting climate change.^145^

By its nature, modeling relies on numerous assumptions, and forest composition modeling inherently provides little opportunity for direct validation. Thus, the safest interpretation may be that the composition of tree species in New York's forests is eventually anticipated to shift in response to climate change, but mature forests are largely resilient to temperature change alone. While recent models suggest forests are not expected to undergo climate‐driven changes in species composition in the near term, mature trees are likely to experience increased stress due to climate hazards and impacts. These include drought and extreme weather events, increases in invasive species, a potential increase in wildfire risk, and a possible increase in land‐use pressure due to projected climate migration. These stressors are in addition to the ongoing nonclimatic pressures from land‐use change, pests, and pathogens (the spread of pests and pathogens themselves is often linked with climate change). Maintaining robust rates of seedling regeneration due to factors such as white‐tailed deer browsing is an ongoing climate‐related challenge to the long‐term viability of New York's forests.^119^, ^146^ Forest tree species composition and the rate of change have varying impacts on ecosystem functions and services. For some functions and services, the composition of the forest matters less, and for other functions and services, the species composition matters a great deal. A tree species lost due to pests or pathogens may be functionally “replaced” (in part or in whole) by another co‐occurring species, resulting in little impact to ecosystem processes such as production, nutrient cycling, water regulation, and climate regulation. Meanwhile, these same compositional changes may have big impacts on other functions or services, such as the loss of commercially valuable timber species or impacts to symbiotic species.^147^

Understory vegetation provides an important contribution to the biodiversity of forested ecosystems globally.^148^ Understory vegetation affects tree regeneration, provides food for herbivores, and can be important to pollinators. Shading by overstory vegetation has limited the warming experienced by understory vegetation.^149^ Nonetheless, changes such as earlier flowering dates have been reported.^150^ Shifts in flowering dates have resulted in predictions of increasing phenological asynchrony in plant−pollinator mutualism, with risk to insect communities.^151^

Researchers have demonstrated the potential for direct impacts to understory vegetation, including changes in community composition and biodiversity, using experiments in which various combinations of climate variables (e.g., temperature, precipitation, nitrogen, carbon dioxide) were manipulated.^152^ Common outcomes observed in these experiments include a lagged response in which changes are not observed until after 10−20 years of experimental duration; changes in community composition but little change in biodiversity; and sharper changes when three or more climate variables are manipulated simultaneously, suggesting the synergistic impacts of climate hazards.^152^ Together, available studies demonstrate a risk of species shifts in understory vegetation that, while lagged from climate change, can occur over shorter time frames than those typically observed for trees. Spring ephemerals are a special class of herbaceous vegetation that rely on the time period before leaf emergence of the overlying trees hastens mortality. Examination of herbarium specimens indicates that spring ephemerals in North America are less sensitive to variation in climate than the timing of tree canopy leaf emergence.^153^ However, the light window before leaf emergence is expected to diminish considerably in the future, which will threaten the fitness of this class of herbaceous plants.^153^

##### 3.2.2.2 Changes in gross forest productivity, growth, and mortality

Gross forest productivity is the rate at which forest vegetation builds new biomass through photosynthesis. This rate may increase in response to longer growing seasons and carbon dioxide enrichment depending on species and location.^126^, ^127^ Warmer temperatures are already increasing the length of the growing season across the state^154^ and are expected to result in a longer period of photosynthetic activity each year. In addition, elevated levels of atmospheric carbon dioxide have a direct, positive impact on photosynthesis and can also improve water use efficiency.^155^ However, a longer growing season and elevated carbon dioxide may not equate directly to increased gross forest productivity.^156^ Productivity may decline when photosynthetic temperature optimums are exceeded, which is expected for species near the southern end of their range, as in spruce‐fir forests.^157^ Enhanced potential productivity may also be diminished by climate‐related stressors such as invasive pest species; more intense drought impacts; and changes that impede regeneration, such as seedling loss to summer drying, competition from more abundant invasive plants, and damage from increased deer browse. Broadly, the potential for increased forest productivity in a warming climate with increased carbon dioxide concentrations may be stymied by the availability of nitrogen, a nutrient that commonly limits tree growth.^158^ Concurrent with increased nitrogen demand, atmospheric nitrogen deposition, a dominant source to forests, has been decreasing in New York State since the 1980s,^159^, ^160^ and will likely further decline in the face of diminishing energy‐related carbon dioxide emissions.^159^ Although warmer temperatures and carbon dioxide enrichment are expected to influence gross productivity, in practice, ecological dynamics are more complicated than the simple relationships presented above. Low‐angled solar radiation at the margins of the growing season is less useful than midsummer sun. Warmer temperatures lead to increased evapotranspiration, so soil water availability does not increase proportionally with increased precipitation.^161^ Additionally, carbon dioxide fertilization interacts with soil nutrient availability and other factors in complex ways. The next subsection examines the potential effects on *net* productivity—after accounting for intensifying climate‐driven stressors and disturbance agents such as fire, insects, and drought.

Regional forest vulnerability assessments have identified several disturbance agents and other stressors that could intensify with climate change and affect tree growth and mortality: wildfire; drought; insects and pathogens; and extreme weather events such as floods, windstorms, and ice storms.^126^, ^127^, ^128^ For each of these potential disturbance agents, the subsections below review the existing research examining historical trends or projecting future changes.

###### Fire, drought, insects, pathogens, and extreme events

Climate change is expected to increase the frequency and intensity of wildfires in many states. Model simulations indicate an increasing but still relatively low risk of wildfires in New York State.^162^ Uncertainties remain, however, so it is presently unclear whether climate change will substantially change wildfire risk, burned area, and associated carbon emissions in New York's forests. In general, projections of increased short‐term drought risk suggest the potential for an increase in wildfire risk, and the replacement of northern hardwood forests by oak forests may increase wildfire risk as well.^127^ Nonetheless, the application of coarse spatial models at the local forest scale is uncertain, suggesting an opportunity for focused investigations that consider variation in factors that affect wildfire risk, such as soil moisture and wind. One study found that the initiation and peak of the fire season in the Northeast is likely to occur earlier in the year, and the high fire risk season is projected to lengthen.^163^ Additionally, as demonstrated by the 2023 wildfires in Canada, boreal forest is at high risk for increasing wildfire frequency in a changing climate.^127^ There is also an increasing risk of poor air quality and resulting risks to human health in New York State in future decades from wildfires that occur outside of the state.^164^

Since the drought that affected New York and the Northeast in the 1960s, a wetter period has ensued, sometimes referred to as an epic pluvial with increasing trends in precipitation and high streamflows.^50^, ^165^, ^166^, ^167^ However, drought risk may increase in the future despite projected increases in annual precipitation because future projections of summer precipitation are highly uncertain and divergent among climate models,^127^, ^168^ lower snowpacks may lead to drier soils in summer,^169^ and hot summers will increase evapotranspiration rates.^161^ Thus far, widespread drought‐related tree mortality has not been observed recently in forests in the Northeast, and analysis of existing studies indicates that a complex set of traits influence drought response and risk.^170^ New York State's Changing Climate discusses drought trends and future risk in further detail.

Ice storms and windstorms play a major role in the dynamics of forests in the Northeast.^171^, ^172^, ^173^, ^174^ There is limited evidence suggesting that ice storms will become more frequent or severe, but this has been identified as an increasing risk in nearby eastern Canada, suggesting the possibility of increased risk to the most northern forests in New York State during the coldest months of the year.^162^, ^175^ However, it is unlikely that climate change will alter these disturbance regimes to a degree that will fundamentally change ecological processes in forests. Nonetheless, such extreme events dramatically affect the forest stands where they occur and could interact with other climate‐related drivers to amplify local impacts to species composition and regeneration.^127^

The intensity of hurricanes and severe convective storms (i.e., thunderstorms) is generally expected to increase, albeit with some uncertainty.^162^ Intense wind events undoubtedly have considerable local impacts on the forests where they occur.^172^ Extreme precipitation events in the Northeast have increased in both frequency and magnitude.^176^ The frequency and intensity of floods has also increased across the Northeast in recent decades, a pattern expected to continue through the 21st century.^177^, ^178^ Large floods can alter forest vegetation on steep slopes and in riparian areas due to erosion. This can impact species composition and regeneration at local scales.^127^

###### Insects and pathogens

How insects and diseases respond to climate change will vary widely based on their life history traits and those of the trees they attack.^179^ Generally, pests and pathogens are expected to become more damaging in forest ecosystems as the climate warms and their ranges, rates of transmission and reproduction, and pace of migration increase.^126^, ^127^, ^180^ The impacts of non‐native pests and pathogens are often particularly acute (refer to Section 3.9.4 and the Hemlock Pest case study for more information on non‐native pests and climate change), but thermal and moisture stress can also increase the susceptibility of trees to native insects such as the forest tent caterpillar^181^ or the spruce budworm.^182^, ^183^ Warmer winters allow for the northward spread of insects that are killed by deep cold temperatures, such as the hemlock woolly adelgid and the southern pine beetle.^169^, ^180^

Few studies have attempted to project future insect damage, in part because the response will be influenced by changes in insect population dynamics, range expansion, or the arrival of non‐native pests—none of which allow for empirical model calibration from observed data. Process‐based models are constrained by the fact that scientific understanding of the mechanisms of forest pest infection, dispersal, and transmission is still in an early stage of development.^180^ Anderegg et al.^184^ derived estimates of insect‐related tree mortality from empirical models calibrated to remeasured U.S. Department of Agriculture Forest Inventory and Analysis plot data and observed climate variables. These researchers did not quantify potential changes in insect pressure related to newly arrived species or within‐population adaptations over the 100‐year time frame they evaluated. The risk of newly introduced insects and pests, while not easily quantified, remains high at present because pest introduction is correlated with global trade volume, which is increasing,^185^ and policies that might limit introduction are not in place.^186^

##### 3.2.2.3 Increasing regeneration challenges

Forest regeneration depends on the growth and survival of seedlings. Germinants and seedlings function differently from mature trees or even saplings.^187^ Climate change could exacerbate the serious regeneration challenges already being experienced across the state and surrounding areas.^188^ Many of the drivers of regeneration failure, such as landscape fragmentation, are independent of climate change, but some—especially overbrowsing by deer^189^ and competition from invasive plants (refer to Section 3.9.4)—could worsen under changing climate conditions. New stressors could also emerge, such as heat‐related seedling mortality^187^ or inadequate seed production from mismatched flower‐pollinator phenology. Seedling density declines with increasing annual air temperature for several tree species that are common in Adirondack forests.^189^

Large herbivores, particularly white‐tailed deer and moose, can have a major effect on forest dynamics.^189^ These herbivores are also sensitive to changes in climate conditions. Moose may be negatively impacted by heat stress and increased parasites associated with climate change, while white‐tailed deer could benefit from increased forage access and lower energy requirements during the winter.^127^ A recently published study indicates that milder winters predicted for the Adirondacks could result in northward expansion of white‐tailed deer populations, which could result in negative effects on regeneration as well as negative impacts on moose populations through parasitic spread.^146^ Changes to the range of moose and white‐tailed deer will also likely influence the composition of tree species.^127^

##### 3.2.2.4 Forest community interactions and compounding effects

The interactions among forest plants and the species that interact closely with plants (e.g., pollinators, seed dispersers, herbivores, fungi, pathogens) are complex and likely to change along with climate, climate‐related disturbances, and ongoing drivers of change in New York's forests. As described throughout this section on forests, disturbances themselves can also interact with one another (e.g., drought increases forests’ vulnerability to insects and forest fragmentation can decrease resilience), compounding disruptions to forests and forest communities.^190^ Longer growing seasons lead to community‐level impacts due to increased damage from freeze‐thaw cycles on plants, animals, and soils; desynchronization of phenological patterns of plants and animals, including wildlife emergence and migration; and decreased climate controls that currently limit pest and pathogen populations. Such community‐level disruptions will result in ongoing and new compounding effects of climate change that are hard to predict.^191^ Studies have documented and hypothesized increasing asynchrony between plants and their pollinators, herbivores, and food webs as a result of climate change.^44^ The function and stability of forest ecosystems rely on a synchronous network comprised of microbes, fungi, plants, insects, birds, and mammals, all of which respond differently to climatological cues. Changes in these cues may occur more rapidly than the adaptation necessary for networks to continue to function. Longer growing seasons can result in multiple generations of insect pests each year, which can increase herbivory and mortality and may provide a competitive advantage to invasive plants such as buckthorn and honeysuckle, which keep their leaves active longer than many native species. Another, more specific example of interactions and compounding effects is the existence of invasive earthworms. While the colonization of the state's forests by invasive earthworms is not caused by climate change, their presence can result in a more climate‐sensitive forest community due to their consumption of leaf litter and impacts on soil structure, increasing forest ecosystems’ vulnerability to air temperature and moisture.^192^

##### 3.2.2.5 Forest economy and equity

The state's logging and forest products industries may be extensively exposed to climate change impacts.^193^ In addition to potential impacts to valued timber species, warmer winters and wetter summers both make for less supportive ground conditions,^194^ leading to reduced access to timber and increased costs for harvesting^195^ and trucking.^196^ These impacts could cascade across the forest products supply chain,^197^ affecting many of the tens of thousands of workers employed in the industry and potentially threatening forests’ role at the center of a sustainable bioeconomy.^198^ Some communities—including Indigenous, rural, and urban communities—may experience greater negative effects of climate change impacts on forests. Indigenous Peoples may experience loss of forest resources that support nutrition, recreation, cultural traditions, and spiritual practices. Rural communities rely on the scenic nature of forests and ecotourism. Urban forests help alleviate extreme heat, particularly for residents of low‐income neighborhoods that do not have access to air‐conditioned buildings.

#### 3.2.3 Regional variation

Although the predominant tree species of New York State are found in all 12 of the assessment regions (refer to the map in the Assessment Introduction), many forest types are restricted in their distributions and subject to different climate impacts. Regional assessments report that forests vary in their vulnerability to climate change.^126^, ^127^, ^199^, ^200^ Highly vulnerable landscapes include coastal forests and tidal swamps, lowland coniferous forests, montane spruce‐fir forests, and northern hardwood forests. Coastal forests and tidal swamps in the South Hudson and Long Island regions are likely to face increasing inundation and saltwater intrusion. Lowland coniferous forests have limited tolerance to changes in hydrology. Montane forests of the Catskills and Adirondacks vary in composition according to elevation, with the more cold‐tolerant spruce and fir stands in higher zones.^201^ Projected long‐term compositional shifts toward warm‐climate species could unfold more quickly along elevational gradients than latitudinally, and recent evidence indicates that these elevational shifts in tree species may already be occurring across mountains in the northeastern United States.^202^ However, the decreasing acidity of precipitation in recent decades is driving enhanced growth rates in some tree species that might otherwise be considered at risk for decline, such as red spruce in the Adirondacks.^203^ At the highest elevations, the future of Adirondack alpine ecosystems remains uncertain (Section 3.4). Tree species in northern hardwood forests are generally shallow‐rooted and more vulnerable to freezing because of the smaller winter snowpack; their wide crowns also increase vulnerability to ice damage.

On Long Island, the Central Pine Barrens are already subject to direct and indirect climate impacts, including summer droughts (which have led to extensive fires) and recent outbreaks of the newly arrived southern pine beetle. These impacts could be a harbinger for future impacts on pine barrens ecosystems.^204^ In general, less diverse forest communities with limited resilience to disturbances and forests located in flatter, lower‐elevation terrain are more vulnerable to climate change. Though lowland forests are well adapted to fluctuating water tables, the impacts of extreme weather events expose these ecosystems to risks of excessive flooding, inundation, and streambank erosion.^128^

### 3.3 Open lands

BOX 4 **Takeaways**

- Open‐land ecosystems are becoming increasingly rare in New York State. Changes in agricultural practices, increasing demand for renewable energy, and a potential increasing future demand for housing in cooler regions will generate new cycles of land‐use change that will make it challenging to protect and manage these ecosystems.
- Low‐lying fields and meadows will be subject to new patterns of flooding, wetting, and drying due to changes in precipitation amounts, intensity, and frequency as well as sea level rise along the coasts. These events will result in changes in plant species composition and corresponding shifts in animal communities.

#### 3.3.1 Description and importance

The New York Natural Heritage Program recognizes 31 forms of native, open‐land terrestrial communities.^4^ Eight of them form on sandy coasts (various dunes, beaches, grasslands, and shrublands); seven are associated with riverbanks (floodplain grasslands and coarse sediment deposits, like gravel bars); four are found on calcareous (high pH) soils (alvar grasslands and shrublands); and eight are restricted to exposed bluffs, cliffs, and rocky summits. The remainder are transitional communities associated with natural and human‐caused disturbances. Of these, the most abundant are old fields undergoing plant succession (Figure 5‐6). Many other open‐land ecosystems are relatively uncommon, and some quite rare, such as those that support alvar (thin calcareous soil‐based) communities. These uncommon ecosystems often harbor rare plant and animal species that would benefit from conservation.^205^

**FIGURE 5‐6:**
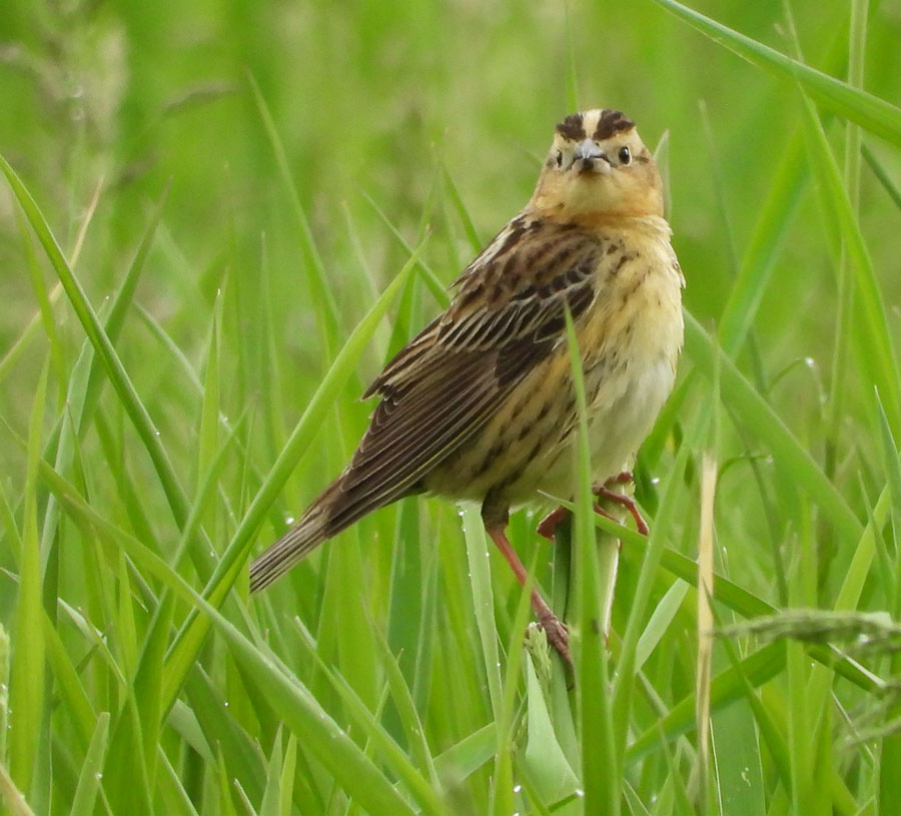
Female bobolink. Bobolinks nest in large, open grasslands. Photo by Peter Waycik.

Most old fields formerly used for agriculture are transitioning to shrub and then forested ecosystems, and as a result, early successional fields have become relatively scarce. Their scarcity poses a problem for wildlife conservation, because these ecosystems also support rare species, including dwindling populations of grassland birds^206^, ^207^ such as the grasshopper sparrow and short‐eared owl. To protect rare species, conservation professionals have adopted active management programs to keep open lands in early successional stages, and their work includes collaborating with farmers who maintain pastures and hayfields (Figure 5‐7).^208^ Given this collaboration, among other reasons, some agricultural ecosystems are an important part of optimal conservation practices on open lands.

**FIGURE 5‐7:**
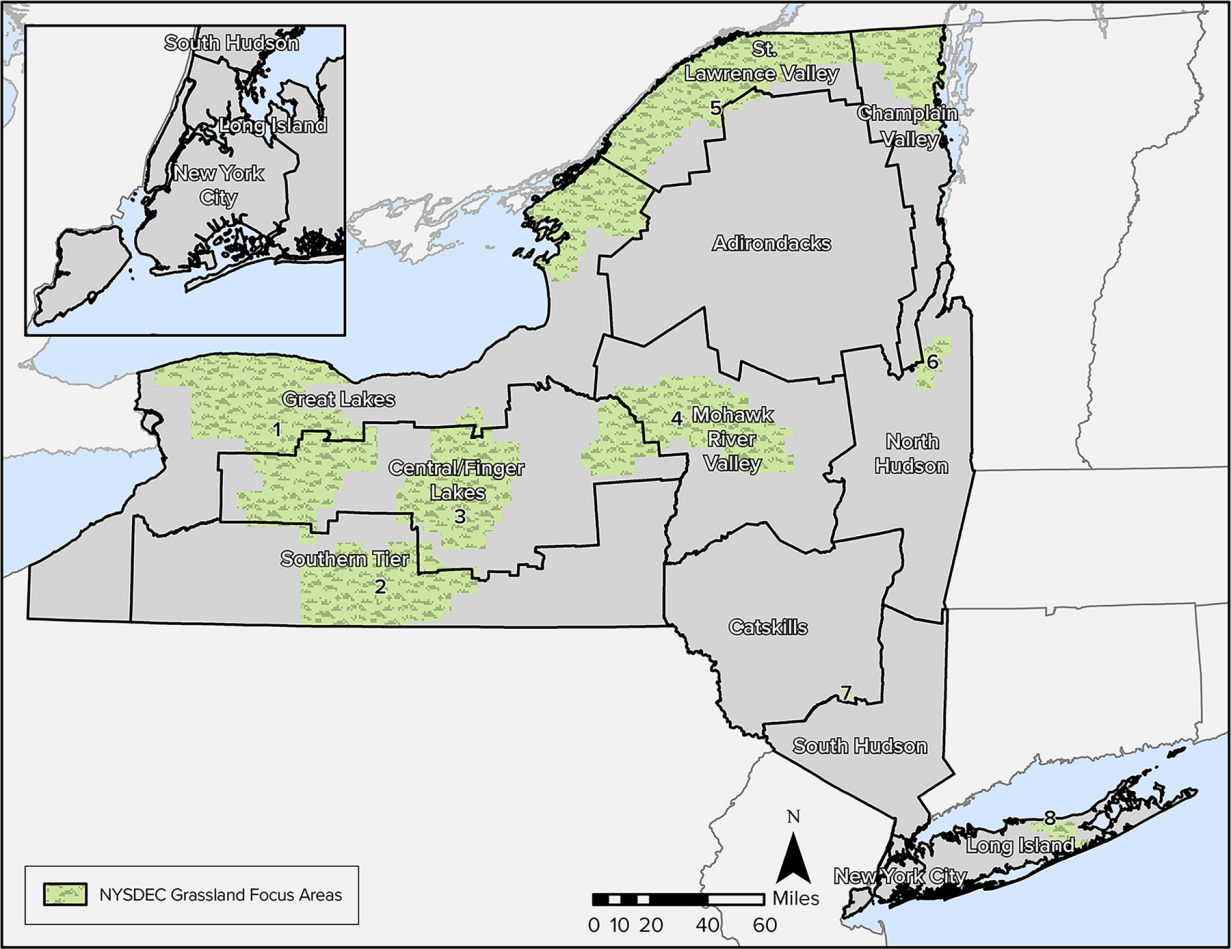
New York State Landowner Incentive Program grassland focus areas. The Landowner Incentive Program for Grassland Protection and Management was administered by the New York State Department of Environmental Conservation (NYSDEC) to conserve high‐quality grassland habitat through easements and management contracts with private landowners in key focus areas. This effort has been replaced by a more refined effort by NYSDEC and partners in Grassland Bird Conservation Centers as outlined in the NYSDEC Strategy for Grassland Bird Habitat Management and Conservation 2022–2027 report.^208^ Data from NYSDEC (2021).^209^

Besides supporting rare species, open lands have other desirable features, such as promoting groundwater recharge and providing seeds for restoration.^210^ Indigenous Peoples in New York have understood the importance of maintaining open land for millennia and have used fire as a tool to reduce forest cover for agriculture and game management.^211^, ^212^ Open lands are home to numerous species of small plants and animals that coexist in dynamic food webs. Because these food webs respond rapidly to annual and seasonal climate variation, open lands represent a dynamic setting that has attracted great scientific interest and extensive ecological experimentation, such as manipulative experiments to model climate change impacts.^213^

#### 3.3.2 Impacts and risks

Open lands are highly exposed systems. All aspects of predicted climate change will have an impact on them. Rising air temperatures and longer seasons will shift competitive relations within plant and animal communities in favor of species with higher thermal tolerances. Longer snow‐ and ice‐free seasons will favor species that can extend their growing seasons. Heavier and more frequent precipitation and more severe storm events will increase flooding in low‐lying fields and erosion in unconsolidated soils like sand dunes and gravel outcrops. The combination of added warmth and more soil moisture may provide advantages to faster‐growing species, and higher rates of productivity may lead to other food web alterations.^214^

Open‐land ecosystems are naturally more dynamic than forested lands, with a higher rate of species turnover and more variable population dynamics.^215^ Therefore, separating the effects of relatively gradual climate changes from the consequences of natural dynamics will be difficult. However, several forms of risk can be anticipated and monitored. Wholesale ecosystem state shifts may occur in response to wetter conditions, such as sparse, grass‐dominated dry alvar grassland being replaced by a denser, sedge‐dominated wet alvar grassland.^4^ In addition, increases in precipitation could accelerate old‐field succession by increasing shrub and tree growth. Other risks, not unique to open lands but important to note, include the following:

- Impacts on plant−animal interactions that are sensitive to synchronized seasonal cycles.
- Competitive replacement caused by the spread of invasive species. Replacement can take place within a few generations for open‐growing, short‐lived species.
- Declines in snowpack, which could affect the survival rates of overwintering species.
- Spread of pathogens due to warmer and wetter conditions. Of particular concern are native and exotic fungal diseases that spread through plant populations in open fields.^216^

#### 3.3.3 Regional variation

Open‐land ecosystems are found in flatter and drier terrain throughout the state. Their association with recently abandoned farmland means that many of these ecosystems are situated in agricultural landscapes, such as the Central/Finger Lakes region. Projections for the latter half of this century show more total precipitation in every region of the state, but with a possibility of more frequent shorter‐term seasonal droughts lasting from weeks to months, most notably in the summer.^162^ Regardless of location, many open lands will be susceptible to changing precipitation patterns. These can lead to wetter or drier conditions that have the potential to alter plant species composition in open grasslands and shrublands, with cascading effects on their fauna.^217^ Stronger cascading impacts will be felt in regions where declines in pollinators lead to declines in agricultural productivity, and where declines in natural predators lead to declines in their biocontrol benefits.

### 3.4 Alpine zones

#### 3.4.1 Description and importance

Alpine ecosystem habitat is found over an area of about 85−175 acres^218^ scattered across several of the High Peaks of the Adirondacks (Figure 5‐8).^218^, ^219^ This alpine terrain is rarely found at elevations below 4430 feet and varies with slope, aspect, and other factors.^219^ Alpine plant communities are characterized by distinct low‐lying biota that includes sedges, grasses, heaths, shrubs, small trees (krummholz), mosses, liverworts, and lichens, in contrast to the boreal forest found at elevations immediately below.^220^ The Adirondack alpine ecosystem is typically grouped with mountaintop alpine habitat found in Maine, New Hampshire, Vermont, and sometimes Quebec, and is believed to have developed as glaciation receded from northeastern North America 10,000 years ago.^221^ The mix of species found in this mountaintop community most closely resembles plant communities found in the Arctic tundra of northern Canada and Greenland.^222^ While common globally, alpine plant species contribute uniquely to regional biodiversity, and many are rare in New York State.^218^

BOX 5 **Takeaway**

Alpine plant communities adapted to harsh winter conditions are expected to persist on Adirondack mountaintops through the end of the 21st century. However, increasing displacement pressure from the boreal forest immediately below, driven by milder winters, is likely to shrink the footprint of alpine terrain in the coming decades.

**FIGURE 5‐8:**
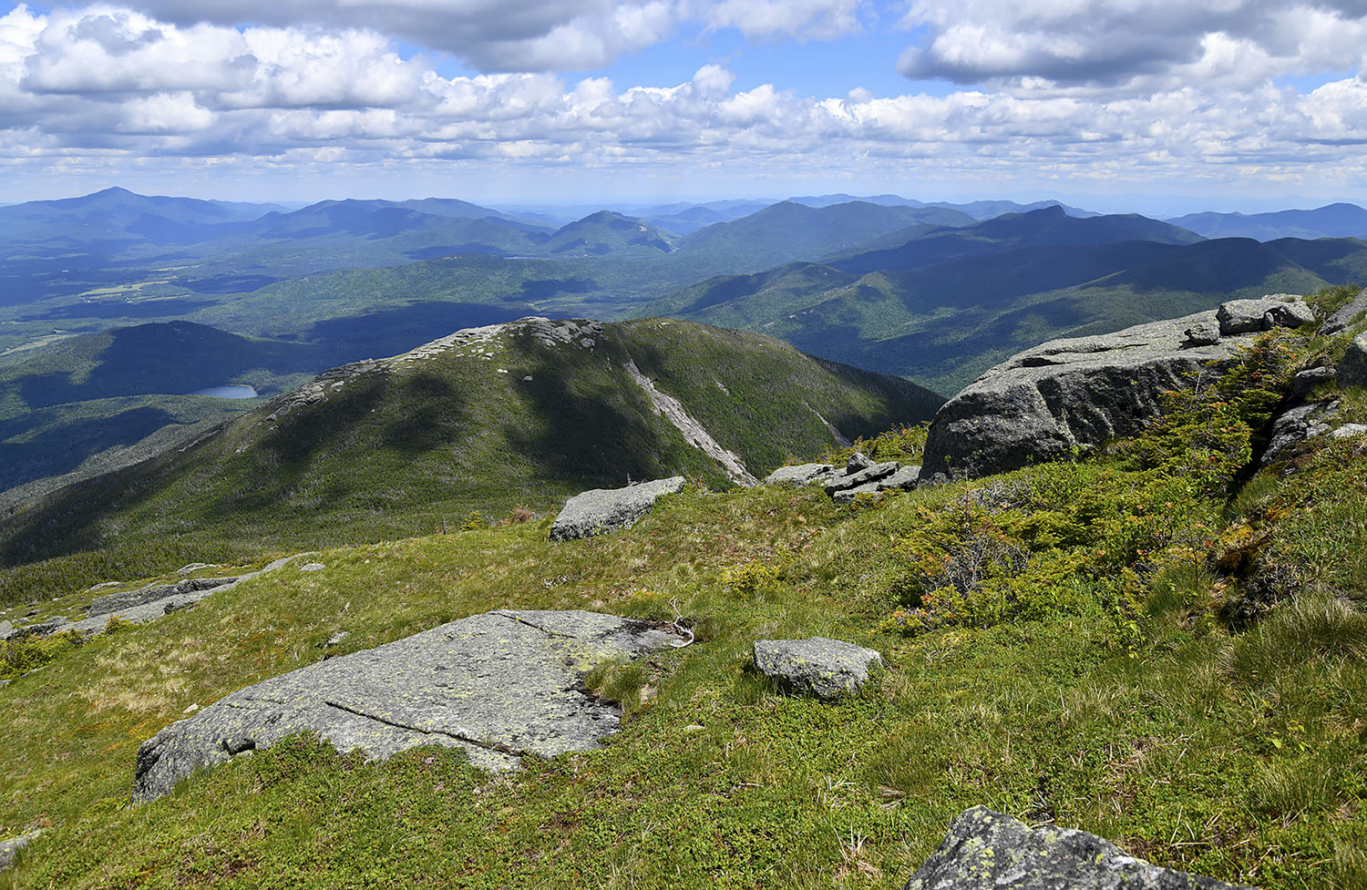
Alpine landscape in the Adirondacks.

#### 3.4.2 Impacts and risks

A common objective of those who have investigated the alpine ecosystems of the Northeast is to determine whether the boundary with the boreal forest below is moving upslope in response to climate change, which would likely reduce alpine habitat.^221^ One group of researchers resampled a set of vegetation plots in the Adirondacks in 2007 that were first established in 1984 and reported an increased frequency of woody shrubs and a decreased frequency of lichens and mosses. These findings were consistent with expected effects of a warming climate, but also consistent with expected successional trends.^223^ However, other studies of alpine ecosystems across the Northeast have reported conflicting patterns that are sometimes consistent with a warming climate^224^ and other times not.^225^ Another researcher applied aerial images over decadal time scales and concluded that the boundary between alpine vegetation and boreal forest was moving upslope by an average of about 10 feet (3 meters) per decade at Mount Katahdin, Maine, and in the Presidential Range of New Hampshire.^144^ This investigator found high variation in the upslope position and in the temporal change of this boundary as a function of landscape and topographic characteristics, but concluded that the trends are generally consistent with expected results of a warming climate. A wide variety of factors affect regional alpine ecosystem plant communities. These include both climatic factors (e.g., predominant wind direction, precipitation, exposure, frequency of rime ice events) and nonclimatic factors (e.g., hiker trampling, air pollutant deposition).^221^ Based in part on investigations of the history of regional alpine ecosystems since the last glaciation, some researchers have concluded that alpine ecosystems will persist on mountaintops of the Adirondacks and the Northeast through the end of the 21st century.^221^ However, species populations are small, and restriction to small isolated patches makes alpine plant communities of the Northeast particularly vulnerable to local extinction.^226^ Furthermore, the pace of projected warming will likely exceed that of past warm periods and the Northeast may continue to experience greater warming than other regions across the globe, which may limit the relevance of conclusions drawn from paleoclimate studies.^227^ The finding that boreal forest may be encroaching on alpine vegetation on some New England mountaintops suggests the possibility that the same trend may be occurring in the Adirondacks, but this possibility awaits confirmation from further study.

### 3.5 Lakes and ponds

BOX 6 **Takeaways**

- Climate change is increasing lake surface water temperatures, decreasing ice cover, and increasing the length and strength of thermal stratification.
- Climate‐induced changes in temperature, ice cover, and stratification are primary contributors to the deoxygenation of lakes. Ongoing and projected future deep‐water deoxygenation represents a major challenge to coldwater fisheries.
- Climate change may be contributing to documented increases in productivity in many lakes, which raises the potential for harmful algal blooms.
- Lake browning is occurring in many New York State lakes. Lake browning modulates climate warming impacts and may exacerbate the stresses of deoxygenation on coldwater fishes.
- Lake levels are responsive to climate and are exhibiting increased variability, which impacts the utility of lake shorelines and shipping routes, especially in the Great Lakes.

#### 3.5.1 Description and importance

Lacustrine habitats, including lakes, ponds, and reservoirs (hereafter, “lakes”), are found in all regions of New York State. Excluding the Great Lakes, lacustrine habitats represent approximately 2.6% of the state's land surface. Some areas are notable for having high concentrations of lakes. For example, lakes make up 7.4% of New York State Indigenous lands and 3.8% of the Adirondacks (Table 5‐1). While lakes represent only a small proportion of the overall land surface, they contain a disproportionally high concentration of global biodiversity.^228^ Lakes also provide a variety of other critical ecosystem services to society, including drinking water and hydropower, and support regional economies through recreational opportunities, aesthetics, and cultural qualities.^229^ Lakes support tourism and act as economic engines in many regions of the state.

The Great Lakes basin holds about one‐fifth of the world's surface fresh water supply. New York State borders two of the five Great Lakes: Erie and Ontario.^230^ Approximately 40 million^230^ people in the United States and Canada rely on Lake Erie and Lake Ontario for drinking water. The lakes also support billions of dollars in tourism, important trade routes, and a large fishery industry, which relies on stable food webs. Lake Erie, the most populated watershed in the Great Lakes basin, is surrounded by agricultural, industrial, and urban land uses and is thus heavily affected by urban and agricultural runoff. Lake Ontario lies downstream of the other four Great Lakes, so it is affected by conditions throughout the basin.

Lakes are highly sensitive to both the direct and indirect impacts of climate change.^231^ Direct impacts result from changes in air temperature, precipitation, and wind speed, as well as changes in the frequency, duration, and magnitude of extreme events related to these climate attributes. Lakes are also sensitive to indirect climate change impacts that occur in their watersheds. Lakes are hydrologically connected to their surrounding watersheds, integrating changes that occur in the landscapes around them.^232^, ^233^ Thus, the response of any given lake to climate change depends on the magnitude and type of climate forcing, interactions with other anthropogenic and natural changes, and the attributes of the individual water body. The sensitivity of lakes to climate change often varies with attributes such as lake depth and water clarity.^83^, ^234^ Hence, lakes have exhibited a wide range of responses to climate change in recent decades.^32^

#### 3.5.2 Impacts and risks

##### 3.5.2.1 Temperature, stratification, and ice cover

Climate change is increasing lake temperatures. In New York State, lake temperatures are regularly monitored by government agencies, academic organizations, and citizen scientist programs such as the Citizens Statewide Lake Assessment Program. Measurements indicate that warming rates vary in different lakes,^32^, ^35^, ^235^ in different seasons,^236^ and at different depths.^35^, ^235^ A worldwide study of lakes found that surface temperatures increased at a median rate of 0.61°F (0.34°C) per decade over the 1985−2009 period.^32^ Other more recent assessments provide similar surface water trend rate estimates of about 0.67−0.70°F (0.37−0.39°C) per decade.^35^, ^235^ Meanwhile, evidence indicates that deep‐water temperatures have not consistently warmed, and in some cases have even cooled.^35^, ^235^ Researchers have also looked at lake heat waves—periods of hot surface water temperatures—and how they may change in response to global warming. One study found that lakes are likely to experience increasingly severe heat waves in future decades. For example, under a very high emissions scenario (SSP5‐8.5), lake heat waves are projected to increase in duration from about 8 days per year (over the 1970−1999 period) to about 96 days per year by the end of the 21st century.^83^ (Refer to New York State's Changing Climate for an overview of emissions scenarios used in this assessment.)

Thermal stratification—the separation of water masses by temperature—is a key physical attribute of lakes that regulates numerous chemical and biological characteristics. In many lakes in New York State and throughout the temperate zone, the combination of warming surface waters and stable deep‐water temperatures has widened the temperature difference between shallow and deeper waters.^35^ This also increases the density difference between water column layers and hence strengthens stratification. Additionally, the seasonal duration of stratification has increased at a rate of 3.7 days per decade, as lakes are stratifying earlier than in past decades, and seasonal summer stratification is lasting longer into the fall.^237^ Models predict that climate change will continue to increase stratification strength in future decades.^83^ Under a fast‐warming scenario, the duration of stratification is predicted to increase by more than 30 days by the year 2100.^238^ These increases in the strength and duration of stratification have numerous implications for the chemistry and biology of lakes. In a study of Wisconsin lakes, fish die‐offs were associated with periods of extreme heat, an association expected to grow stronger through the 21st century.^239^

Ice cover records represent some of the longest continuous data sets of the effects of climate change on ecosystems. For several New York State lakes, ice cover records reach back more than a century, documenting the onset, breakdown, and duration of winter ice cover. Many lakes with long‐term records have displayed a clear and substantial decline in the duration and extent of ice cover.^240^, ^241^, ^242^ For example, Mirror Lake in Lake Placid has seen its ice‐on (freeze) date shift later by 11 days since 1903; its thaw date has shifted 6 days earlier since 1905.^243^ Lakes George and Champlain, which have ice cover records extending back more than a century, have exhibited an increase in the frequency of ice‐free winters in recent decades.^244^, ^245^ An analysis of ice phenology for five lakes in the Adirondack Mountains between 1975 and 2007 revealed a rapidly decreasing number of days in ice cover (up to 21 days less) primarily due to later freeze‐up.^246^ Over the 1973−2020 period, the duration of ice cover has declined in all five Great Lakes by 8−46 days.^247^ Ice cover losses are projected to continue in future decades. For example, ice cover on Lower Saint Regis Lake in the Adirondacks now thaws 1 week earlier than it did in the earliest year of monitoring (1909), and the lake is expected to lose an additional 1−3 weeks of ice cover by year 2100.^248^ Ice cover loss can impact stratification and dissolved oxygen levels; disrupt food webs; reduce cultural ecosystem services such as ice fishing, skating, and hockey; and increase risks associated with winter ice activities.^249^, ^250^

Just as climate change affects lakes, some lakes in the state have discernable impacts on the local and/or regional climate. For example, the Great Lakes directly influence the climate of Western New York through lake‐effect precipitation and reduced daily and seasonal variation in temperatures associated with the lakes’ large thermal mass. Ice cover losses and warmer water temperatures associated with climate change increase the amount and spatial extent of lake‐effect precipitation, and snow in particular.^251^

##### 3.5.2.2 Dissolved oxygen

Dissolved oxygen is considered a “master variable” regulating numerous aspects of aquatic ecosystems. Nearly all complex life depends on oxygen, and the loss of oxygen, termed deoxygenation, fundamentally alters chemical reactions. Deoxygenation can proceed to the point of onset of anoxia, or absence of oxygen, which often leads to the production of methane. Anoxic habitats are often substantial sources of methane to the atmosphere. Additionally, anoxic sediments often release phosphorus to the overlying water column,^252^ which can stimulate high algal growth and lead to conditions favorable to harmful algal blooms (HABs).^253^ Lakes throughout the temperate zone worldwide (including the Northeast) are undergoing deoxygenation at rates up to 10 times the rate of ocean deoxygenation.^35^ Assessment team analysis of a data set from 28 lakes in the Adirondack region^254^ found that, over the 1994−2012 period, dissolved oxygen declined in shallow waters at an average rate of ‐0.16 milligrams per liter per decade and in deep waters at an average rate of −0.64 milligrams per liter per decade. Another analysis of 11 stratified Adirondack lakes from 1994 to 2021 found minimal change in shallow waters but an average decrease at a rate of −0.25 milligrams per liter per decade below the thermocline (the boundary between upper and lower layers in a stratified lake).^255^ These rapid dissolved oxygen losses are faster than the median rates observed throughout the temperate zone.^35^ At present, it is unclear to what extent these Adirondack data sets represent trends across all New York State water bodies.

Dissolved oxygen concentrations are affected by several climate‐related changes to lake ecosystems, including increases in water temperature, increases in the strength and duration of stratification, and decreases in the duration of seasonal ice cover. Surface‐water deoxygenation is primarily due to solubility losses associated with higher temperatures, which is a concern for shallow‐water species because oxygen metabolic demand increases with temperature.^256^ In contrast, deep‐water deoxygenation is more often associated with stronger stratification and earlier onset of stratification.^237^ Earlier onset of stratification, which often results from ice cover loss, provides more time for seasonal deep‐water oxygen depletion to occur.^257^ Predicted increases in the duration of stratification are likely to exacerbate deoxygenation in the coming decades.^238^ Thus, while deep‐water temperatures have remained stable in many lakes in recent decades, ongoing deoxygenation is a growing and substantial threat to biodiversity in New York State, especially to coldwater fishes.^35^

##### 3.5.2.3 Lake productivity and algal blooms

Lake primary productivity is a measure of the amount of photosynthesis that occurs over a given time interval. Because photosynthesis is in part a temperature‐dependent process, rising water temperatures may increase the amount of primary productivity in lakes, resulting in impaired water quality conditions. Additionally, rising water temperatures tend to favor the proliferation of cyanobacteria, which can produce HABs.^258^ (Refer to the Harmful Algal Blooms case study for more information on plans for addressing HABs in New York State.) Some evidence indicates that there has been a global increase in lake primary productivity, as indicated by phytoplankton blooms, since at least the 1980s.^259^, ^260^ Though inputs of nutrients from human‐modified landscapes (e.g., agricultural and urbanized landscapes) are often an important factor stimulating algal blooms, there is no clear single driver of this phenomenon.^260^ Other studies indicate that there has been no widespread increase in algal bloom severity, duration, or occurrence in recent decades in the United States.^261^ Climate‐induced increases in thermal stratification may reduce algal biomass in large lakes by inhibiting the movement of nutrients from deep waters to surface waters. However, increases in thermal stratification also increase anoxia (refer to Section 3.5.2.2) and the release of bound phosphorus from sediments, thereby potentially increasing algal biomass following seasonal mixing.

Climate change may be most important in facilitating increases in lake primary productivity and HABs in lakes that already have high nutrient levels. For example, in a study of 188 globally distributed lakes, warming was associated with chlorophyll increases in lakes with high baseline levels, but was associated with chlorophyll decreases in lakes with low baseline levels.^262^ However, increases in algal blooms have been reported even in nutrient‐poor lakes.^263^ In general, lake primary productivity and algal blooms are sensitive to both climate conditions and land use. Increases in algal biomass and cyanobacterial blooms are predicted in areas where both urban land use and water temperature are increasing and forest habitat is decreasing, such as in the Lake Champlain basin in New York.^264^

In the Great Lakes region, rising air temperatures have resulted in more frequent freeze‐thaw cycles, and an increase in snowmelt has increased nutrient loading to the lakes.^265^ Increases in extreme precipitation events also generally result in increased nutrient loading to lakes, including to the Great Lakes, which can restructure phytoplankton communities and alter ecosystem function.^266^ A 6‐day study in Lake Michigan found that approximately 70% of total nitrogen and phosphorus present in the lake was introduced by its largest tributaries.^267^ In Lake Erie, nearly all the water inflow comes from the Detroit River and its tributaries, indicating that runoff from precipitation has a large effect on nutrient influx. Evidence also indicates that a decrease in seasonal ice cover in Lake Erie has facilitated winter blooms of diatom plankton,^268^ which likely results in winter transfer of carbon to bottom sediment, where it may be later respired in summer and contribute to eutrophication.^269^

##### 3.5.2.4 Lake level

Lake level or depth is a key attribute regulating habitat availability and numerous ecosystem services within lakes. For example, navigational channels often require specific minimum depths, and low water levels reduce the preferred nearshore (littoral) habitat for many fish species. Often, a change in water level in a large lake, such as one of the Great Lakes, equates to a substantial change in stored volume and surface extent. Variations in lake level are often linked with variations in the hydrologic cycle, including changes in the balance between precipitation and evaporation. By extension, lake level is also linked with atmospheric and oceanic circulation patterns and net runoff. Since 1992, more than half of the variation in the water levels of 200 globally distributed large lakes has been attributed to climate drivers,^270^ and water depth has been found to be a key predictor of lake sensitivity to climate change.^32^ Government agencies track water levels closely in large lakes like Lake Erie and Lake Ontario. While lake level is not as uniformly tracked in smaller inland lakes, research indicates that these smaller lakes respond synchronously with larger regional lakes.^271^

In the Great Lakes, the water level is monitored closely because of its importance to neighboring urbanized areas, flooding potential, and shipping. Variable water levels can be dangerous. For example, in 2017, flooding damaged Lake Ontario coastal communities as lake levels rose to record heights.^272^ Large standing waves, called seiches, also present a danger due to reduced ice cover, variable water levels, and stronger storms.^273^ Human activities have “hardened” the Great Lakes’ shorelines over time, reducing the capacity of natural features such as dunes, beaches, bluffs, and wetlands to provide protection from flooding and waves.

A management plan, termed Plan 2014, regulates lake levels for Lake Ontario and the St. Lawrence River. The plan provides for relatively natural variations in water levels as a way of supporting diverse wetland biotic communities.^274^ Projections of future lake levels are often poorly constrained because water depth is sensitive to both climate and nonclimate factors. Models indicate that future warming is likely to lead to greater variations in lake levels with more rapid transitions between extremes, further challenging management efforts.^275^ The Water Resources chapter provides additional information about shared governance of the Great Lakes in response to climate challenges.

##### 3.5.2.5 Lake browning

Browning is a term used to describe increases in the concentration of dissolved organic matter (DOM) in lakes and streams. High concentrations of DOM give lakes a brown “tea‐like” color that is common in many heavily forested regions throughout New York. Lake browning has been documented throughout the Adirondacks,^276^, ^277^, ^278^, ^279^ in other parts of the northeastern United States, and in northwestern Europe.^280^, ^281^ The primary cause of lake browning is thought to be ongoing recovery from historical acid deposition, as increasingly dilute and less acidic water has expanded favorable habitat for many organisms as the flux of DOM from watershed soils into lakes has increased over time.^280^ However, some evidence indicates that climate change is also a contributing factor to increased lake browning.^281^, ^282^, ^283^ Warmer temperatures and a longer growing season lead to increased plant growth and also enhance the breakdown of soil organic matter into DOM where it can be mobilized from the terrestrial to the aquatic environment. Increases in precipitation increase the extent and inundation period of wetlands, which serve as an important source of aquatic DOM.^284^ Increases in precipitation also increase terrestrial DOM loading. Thus, ongoing climate change may be exacerbating lake browning and will continue to do so for decades to come. But, at present, it is difficult to disentangle the effects of climate change from other anthropogenic impacts, and from acidification recovery in particular.

Lake browning modulates the impacts of climate warming on lake temperatures. Overall, established relationships suggest that lake temperatures should increase at a rate that is about 70%−85% of the rate of air temperature increases.^285^ However, researchers have documented lake warming rates in excess of air temperature warming rates in many lakes.^32^ These high warming rates can be induced by several factors, one of which is water clarity changes. Specifically, declines in water clarity, which result from lake browning, can amplify surface temperature warming rates.^286^ This occurs because heat is trapped in a thinner depth range in low‐clarity conditions, resulting in higher temperatures relative to those that occur when clarity is high. It should be noted, however, that while water clarity declines amplify surface temperatures, the concentration of heat absorbance in a thinner depth range results in cooler deep‐water temperatures and often a volumetrically lower temperature.^286^, ^287^ Increasing surface water temperatures and cooling deep water temperatures have the net impact of increasing the strength of stratification, which acts as a barrier to the passive dispersal and movement of organisms, gases, and nutrients between deep waters and surface waters. Additionally, DOM‐associated nutrients may stimulate algal blooms during seasonal turnover. While lake browning preserves deep coldwater fish habitat,^287^ phytoplankton biomass or DOM acts as a substrate fueling oxygen‐consuming respiration,^35^ and therefore, may further stress coldwater fishes despite stable temperatures. The net effect of lake browning may be to shift the primary climate‐induced stress on coldwater fishes from temperature to oxygen.

#### 3.5.3 Regional variation

Lakes vary in characteristics such as size, depth, trophic status, and landscape position throughout New York State, and therefore, lakes will likely exhibit diverse responses to climate change within and among regions. In general, as air temperature warms, water temperature increases volumetrically to a greater extent in clear and shallow lakes than in deep and colored lakes.^234^ Watershed land use is another primary factor regulating lake responses to climate change. Watersheds with high amounts of human‐dominated land use (e.g., agricultural land cover, urbanized landscapes) face a substantial risk of detrimental climate impacts due to climate‐induced increases in algal biomass, as well as precipitation‐mediated increases in inputs of nutrients from the landscape, such as nitrogen and phosphorus.

With precipitation increasing overall, lake habitats are likely to persist throughout the state. However, extreme precipitation events are also increasing in frequency and severity, and these events can flush nutrients such as nitrogen and phosphorus, as well as organic carbon, into water bodies. Therefore, efforts to preserve or improve water quality must account for this nonstationarity in climate, and proactive management may be necessary just to maintain status‐quo water quality conditions.

### 3.6 Wetlands

BOX 7 **Takeaways**

- Climate change will have a disproportionate impact on wetland ecosystems that are most vulnerable due to location, existing pressures, and size. These include coastal wetlands; riverine wetlands in developed watersheds; and small, isolated wetlands such as vernal pools.
- Large, connected wetlands and wetlands within a wetland complex will buffer climate impacts and provide connectivity for wildlife movement and migration in response to climate change.
- Changing hydrology associated with climate change, including an increase in extreme storm events, will challenge the ability of wetland managers to restore and manage wetlands using historic hydrological models and techniques.
- Negative impacts on wetlands from nonclimate land‐use stressors will continue to be more substantial than projected impacts associated with climate change.

#### 3.6.1 Description and importance

New York State hosts more than 50 types of wetlands throughout its major regions.^4^ As Figure 5‐9 shows, wetland ecosystems are dense throughout the Great Lakes, St. Lawrence Valley, and Adirondacks regions.

**FIGURE 5‐9:**
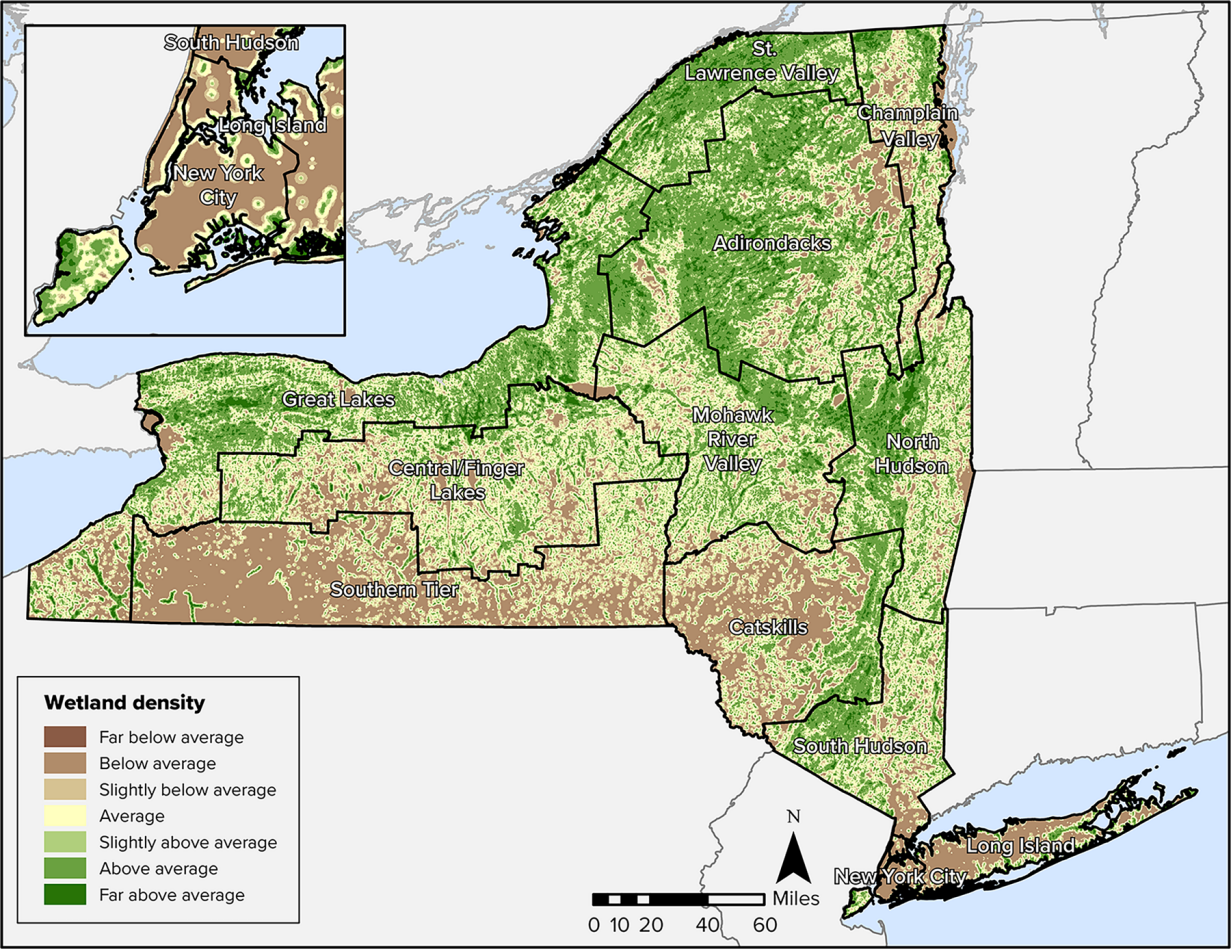
New York State wetland density. Wetland density is measured by the weighted density of wetlands (National Wetland Inventory, NLCD 2001) compared with the regional average, following methods described by Anderson et al.^289^ Data from Anderson et al.^290^

Wetlands range from large swamps and marshes covering thousands of acres to small fens, bogs, vernal pools, and seeps. More than 70% of wetlands in New York State are forested. Wetlands are found along rivers, lakes, and coasts (coastal forested wetlands and salt marshes), and within the Hudson River estuary (tidal wetlands). Wetland complexes are mosaics of numerous and diverse wetlands interspersed with uplands, as seen in the Montezuma Wetlands Complex in Central New York.^288^

Often located at the transition between upland and aquatic habitats, wetlands support a diverse assemblage of plant and animal species including invertebrates, fish, amphibians, reptiles, birds, and semi‐aquatic mammals. Many of these species are rare, threatened, or endangered in New York, including the bog turtle, Atlantic Coast leopard frog, black rail, and bog buckmoth, along with a growing list of plants that includes several species of rushes, sedges, and orchids. Some wetlands provide important climate refugia for plant and animal species vulnerable to climate change. For example, balsam fir trees were shown to be less sensitive to warming summer temperatures in microclimates created by groundwater‐fed fens than in adjacent upland soils.^291^

In addition to offering wildlife habitat and supporting biodiversity, wetlands provide a number of other critical ecosystem services, many of which contribute to climate resilience in watersheds. These ecosystem services include soil retention, groundwater recharge, nutrient and toxin filtration, carbon sequestration, floodwater storage, shoreline protection, and aesthetics. Wetlands have an important impact on water quality, as they intercept, filter, and absorb sediments and pollutants in surface runoff before it enters aquifers, streams, rivers, lakes, and the ocean.^288^

While some wetland areas are protected by state and federal regulations, including New York's Freshwater Wetlands Act, many wetlands remain unprotected. Additionally, federal wetland regulations are subject to change, which sometimes results in reduced protection for many wetland systems. Overall, New York has lost almost half its wetlands^292^ since European colonization. The losses continue today, largely due to the draining and filling of wetlands for agriculture and development. A small net gain in wetland area was recorded between the mid‐1980s and mid‐1990s due to the abandonment of agriculture and increases in runoff in developed watersheds. While these naturally reverting wetlands provide some ecological services, they are typically low quality, dominated by invasive species, and unlikely to offset the continued loss of higher quality wetlands at a landscape level.^293^ Climate stressors are expected to combine with nonclimate stressors to result in major detrimental impacts to wetlands and the species that depend on wetlands, particularly those with low dispersal ability.^294^ Shrinking area has been the dominant outcome to date, driven by either reduced land area (i.e., from filling or increased sedimentation of wetlands) or reduced water availability (i.e., from water diversion or wetland drainage).

#### 3.6.2 Impacts and risks

##### 3.6.2.1 Changing hydroperiod

Wetland ecosystems are sensitive to small changes in seasonal inundation and water levels. Precipitation in New York State is projected to increase by 6%−17% by the 2080s, relative to a 1981−2010 baseline, and the rate of sea level rise along New York's coasts is faster than the global mean.^162^ However, warmer temperatures will likely increase evaporative loss from some wetlands and may stress wetland or wetland‐adjacent species during periods of drought. Predicting impacts to wetlands due to temperature and precipitation increases and sea level rise is difficult and specific to location, wetland type, and adjacent land use. Climate change is projected to affect inland wetlands through a temperature‐related increase in evaporation that will decrease total wetland area, changes in the spatial distribution of wetlands and wetland types on the landscape, and a change in the spatial distribution of wetland‐dependent species.^295^, ^296^ One study modeling changes in tidal wetland habitats in response to sea level rise in the Hudson River estuary projected an increase in net tidal wetland area.^297^ Flooding associated with an increase in total precipitation and extreme precipitation events is projected to have variable impacts on aquatic systems, including wetlands. One recent literature review found that small‐magnitude floods (less than 10‐year recurrence) had neutral or positive impacts on ecosystem services such as primary production, water regulation, and recreation and tourism, whereas large‐magnitude floods (greater than 100‐year recurrence) resulted in a uniform loss of ecosystem services.^298^

If projected changes in snowfall (decreased snowpack) and temperature (increased temperature) culminate in a reduced hydroperiod (the duration of water coverage in wetlands), there may be a loss of specialist plant and animal species that rely on ephemeral wetlands/vernal pools.^299^ More frequent and extreme storm events, along with warming, sea level rise, and shifting patterns of precipitation and drought, could have detrimental impacts that affect some wetland ecosystems more than others. For example, during the course of one extreme storm event, a small riparian wetland can be eroded by scouring or buried completely by sedimentation. An excessive influx of water, nutrients, sediment, or toxins into a wetland from an unusually intense storm can result in eutrophication (excessive growth of algae and plants caused by enhanced nutrient availability) or other threats to wildlife that can temporarily or permanently remove wildlife from the wetland habitat. Coastal wetlands are particularly vulnerable to storm events, as sea level rise and freshwater inflows can combine to amplify impacts.^8^

Projected sea level rise and saline groundwater intrusion along New York State's coasts will affect tidal wetlands that are sensitive to the extent of tidal fluctuations, as well as freshwater wetlands adjacent to coastal zones. At the current rate of sea level rise, it is unlikely that even unobstructed coastal wetlands will migrate inland at the pace of projected changes in tide elevations and coastal flooding. However, tidal wetlands lost to inundation from sea level rise in the Hudson River estuary could be offset by tidal marsh migration into upland areas, which could result in an increase in total wetland extent of about 5600 acres (2260 hectares) by the year 2100 and a shift in the composition of tidal wetlands as high marsh decreases and low marsh increases.^297^ The impact of sea level rise on wetlands is influenced by factors such as adjacent land use, density of development, and differences in the adaptive capacities of freshwater and saltwater systems. Freshwater wetlands adjacent to coastal zones will be negatively affected by saline groundwater intrusion and more extensive storm surge.^296^ Refer to Section 3.8 for a more detailed discussion of sea level rise and additional information about coastal wetland ecosystems.

##### 3.6.2.2 Wetland wildlife habitat and wetland management

Erosion, pollution, and runoff of excessive nutrients are factors that affect the availability and quality of wetland habitat for wildlife. These impacts are expected to increase with an increase in extreme storm events, which will, in turn, negatively impact wetland flora and fauna. Migratory waterfowl and waterbirds are particularly sensitive to changes in the availability and quality of wetlands. Waterfowl and shorebirds, for example, rely on the physical protection of wetlands as well as the food they provide (e.g., plants, insects). Because migratory birds provide important recreational opportunities for hunting and bird watching, changes in the overall availability and quality of wetlands will have cascading impacts on birds, recreation, and communities across New York State.^300^

Natural resource agencies manage many of the large wetland complexes in the state specifically for migratory waterfowl, waterbirds, and other wetland‐dependent wildlife. Such managed wetland impoundments and associated infrastructure (dams, berms, spillways, and other water control structures) are designed to provide specific wetland habitat at certain times of the year based on historic climate and hydrologic conditions. Changes in temperature, precipitation, extreme storm events, and evapotranspiration rates could require replacement of infrastructure and/or adaptive management (an iterative strategy in response to current uncertainty that may change over time) regimes to enable natural resource managers to provide suitable wildlife habitat through wetland restoration and management actions (Hess PJ, Wildlife Biologist, Iroquois National Wildlife Refuge [2022, March 5, Personal communication]).

As rising temperatures affect the growing season, precipitation patterns, and thresholds (e.g., first and last frost dates, frost duration, snowpack), phenological changes may occur in wetland ecosystems. While such changes are difficult to forecast, they may include shifts in the timing of insect emergence, bird migrations, and the life cycles of endemic animals (e.g., amphibians and reptiles). These changes could result in the loss of synchronicity among interacting species, leading to changes in food availability and species abundances in wetlands. Several state‐listed species inhabit wetlands, including Blanding's turtle, bog turtle, pied‐billed grebe, black tern, least bittern, and sedge wren.^301^ Such species could suffer further decline if wetlands are negatively affected by climate change. Some of these species, such as the endangered bog turtle, are habitat specialists that have low mobility and are experiencing high habitat fragmentation. The bog turtle was classified as “extremely vulnerable” to projected climate change in a 2011 report by the New York Natural Heritage Program.^302^

##### 3.6.2.3 Cultural wetland values

Indigenous and rural communities value wetlands as cultural and traditional resources. For example, several Tribal Nations have a tradition of using black ash, a wetland species, for making snowshoes and weaving baskets. The Fond du Lac Band of Lake Superior Chippewa cite the negative cascading effects of tree loss due to the spread of the emerald ash borer, including changes in hydrology within wetlands and watersheds.^73^ Many rural communities rely on ecotourism associated with wetlands and wetland widlife. Wetland degradation and loss of wildlife will have a negative impact on rural communities and reduce opportunities for recreational activities such as nature photography, birdwatching, hunting, and fishing.

##### 3.6.2.4 Regional variation

The degree and extent of climate change impacts on wetland ecosystems will vary depending on wetland type, ecoregion, and geography (Figure 5‐10).^303^ Many wetland ecosystems—particularly large deepwater emergent marshes, forested wetlands, wet meadows, and scrub‐shrub wetlands—are resilient to seasonal and annual variability in precipitation because of their capacity to store and retain water. Wetland flora, fauna, and ecological processes may be more resilient to climate impacts in large wetlands and/or in a wetland complex, where multiple wetlands in close proximity often have a relatively high degree of hydrological and ecological connectivity. Such systems offer more options to plant and animal species with limited mobility than do fragmented or isolated wetlands.^289^ Large wetlands and wetland complexes are found in areas of New York State with lower elevations and flatter topography, such as the Great Lakes and St. Lawrence River Valley assessment regions.

**FIGURE 5‐10:**
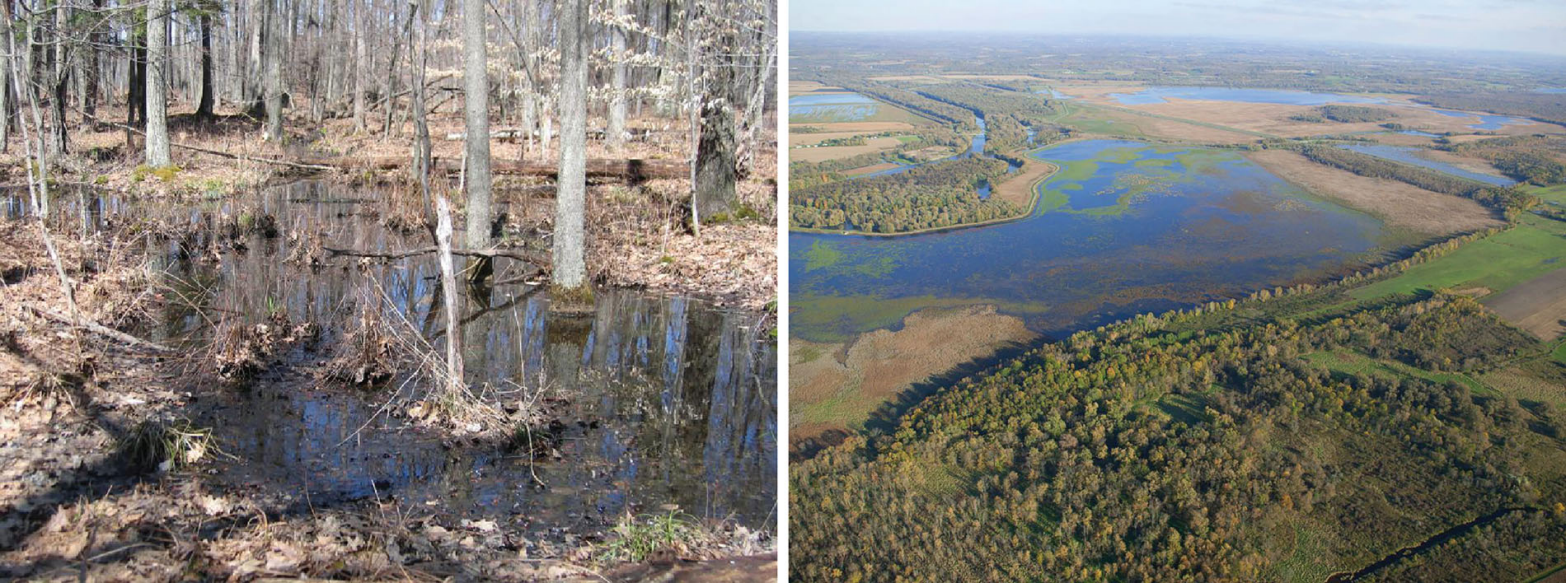
A wetland's size, type, and landscape position all affect its degree of vulnerability to climate change. The small vernal pool in a hardwood forest setting in the photograph on the left provides important and very specific breeding habitat for salamander species. If snowpack, timing of snow melt, and spring runoff change substantially, many of these systems may become unsuitable habitat or a habitat sink. Extensive emergent marsh and forested wetland systems like those in the Montezuma Wetlands Complex (right) are more resistant to climate change impacts and allow greater resilience for the species that depend on them and the ecosystem services they provide. Photos by New York Natural Heritage Program (left); Bill Hecht (right).

Small, ephemeral, or isolated wetlands such as vernal pools—and rarer wetlands such as bogs and fens—may be less resilient to substantial changes in snowpack, precipitation, temperature, and extreme events.^304^, ^305^ Because small wetlands tend to have lower water levels or smaller reservoirs of water, they are more susceptible to increased evaporation rates, which can impact water depth, availability, and temperatures. The unequal vulnerability of smaller wetlands could be exacerbated by existing policies for protecting wetlands, although the New York State Freshwater Wetlands Act was recently amended to include protection of smaller wetland systems (e.g., vernal pools) by 2025. Wetland ecosystems at the southern limit of their range may have greater vulnerability to climate change. A study of tree encroachment in boreal peatlands in the Adirondack Mountains showed measurable changes in plant community composition due to climate change. Small remnants of peatlands were at the highest risk of impact.^306^

In the future, wetlands will continue to suffer from nonclimate stressors such as ongoing and potentially accelerating development and fragmentation of land, filling of wetlands, contaminated runoff due to agriculture, and increased pressure from invasive species. Climate change could reduce many wetlands’ viability and presence even further.

### 3.7 Riverine ecosystems

BOX 8 **Takeaways**

- Extreme climate events (particularly those that lead to flooding) and seasonal changes in hydrology caused by shorter, warmer winters will pose the most urgent management challenges for preserving riverine biodiversity, improving water quality, and protecting life and property throughout New York State.
- Agricultural watersheds are sensitive to heavier precipitation that overwhelms floodplains, inundating cropland and moving contaminants into creeks and rivers. Improved floodplain planning and management can address this issue.
- Urban watersheds are especially prone to flood damage. The pressing need for better stormwater control will grow, as will the need for improvements to the functional properties of urban stream and river ecosystems.

#### 3.7.1 Description and importance

The term “riverine” refers to flowing water bodies embedded in watersheds that capture and transport water, organisms, and materials. New York State has 17 major watersheds,^307^ which are distributed across the 12 climate assessment regions and connect to the Atlantic Ocean and the Gulf of Mexico via the Delaware, Hudson, Mississippi, Susquehanna, and St. Lawrence rivers. As water moves downstream from headwaters to the sea, systematic changes in species composition and ecological processes are often observed. Human activity typically increases in scale and intensity downstream, which influences water quality, flow, and aquatic ecology along riverine ecosystems. The New York Natural Heritage Program lists 15 types of riverine systems in the state based on physical and biological properties, with nine considered natural and six designated as “cultural.”^4^ To address climate impacts, this section considers three main categories of river systems: rural watersheds (low human population densities with few or no farms), agricultural watersheds (landscapes dominated by working farms), and urban watersheds (landscapes dominated by human infrastructure). Each category has unique properties, vulnerabilities, and management challenges.

All three watershed categories share a common set of traits, which may be affected by climate change:

- **Source waters** arrive as overland flow, from groundwater, and from standing water bodies.
- **Instream flows** travel in channels (natural or restricted) over beds composed of various organisms and materials that vary with water depth, surrounded by floodplains of varying dimensions and functionality.
- **Channel geometry** (longitudinal/cross‐sectional) and **stability** are functions of flow rates, flood frequency, and other factors.
- **Connectivity** within the watershed network allows the movement of organisms and materials between small and large streams and rivers and can be considerably impeded by natural and artificial barriers.
- **Sediment transport and deposition** within watersheds varies with sediment sources and character and with the magnitude of streamflow.
- **Nutrient concentrations** (particularly carbohydrates, nitrogen, and phosphorus) vary throughout watersheds and determine the composition of, and transfer of energy within, food webs.^308^, ^309^

#### 3.7.2 Impacts and risks

Several relevant climate impacts are anticipated in riverine systems. More frequent rainfall, heavier rainfall, and more extreme storm events could alter channel and floodplain geometry, disturb protective riparian vegetation, disrupt populations and communities of aquatic species, and raise seasonal water levels. Decreased snowfall will lead to less spring runoff and change the timing of spring peak‐flow periods, especially in cooler regions. A reduction in the number of freezing days will raise spring water temperatures, affecting the seasonal reproductive cycles of aquatic plants and animals (more so in cooler regions). Warmer average air temperatures will yield warmer, oxygen‐depleted waters and create more stress for coldwater organisms statewide. Although total annual precipitation will rise, evolving weather patterns could lead to more frequent or extreme droughts in regions across the state.^310^

##### 3.7.2.1 Rural and agricultural watersheds

Aquatic habitat warming will alter biodiversity by reducing or eliminating coldwater species (e.g., brook trout),^2^ which will alter rates of productivity.^311^, ^312^ Changes in the timing of seasonal high‐flow events and more frequent and intense flooding will alter channel morphology and stability and disrupt the life cycles of fish^313^ and stream‐breeding aquatic species,^314^ affecting the reproductive success of trout.^315^ Conversely, changes in water levels can widen the distribution of some species, such as North American beaver^316^ and certain aquatic plants, which can further alter streamflow and sediment transport. In addition, some riverbank ecosystems historically shaped by seasonal climate and flow dynamics (e.g., Hudson River ice meadows)^317^ could be permanently altered by shifting norms and extreme events.

Increased contamination from fertilizers, pesticides, animal waste, and septic systems could follow unusually heavy rainfall events and enhance the growth of harmful algae, which in turn suppresses animals.^318^, ^319^ In many cases, potential contamination is exacerbated by narrow or nonexistent shoreline vegetation buffers, which would otherwise intercept and modify contaminants.^320^ Farm ponds could overflow more frequently as precipitation increases, carrying nutrients and non‐native organisms into nearby streams and rivers. In addition, an increase in the frequency and severity of storm events could alter channel geometry through excessive bank erosion and floodplain deposition, which can eliminate or bury adjacent farm fields.

##### 3.7.2.2 Urban watersheds

Due to the high percentage of impervious surfaces in urban watersheds, any increases in the frequency and intensity of precipitation events will continue to stress confined channels, leading to incision, reduced filtration, increased surface runoff, and reduced riparian groundwater levels.^321^, ^322^ Efforts underway to reduce runoff and contamination from combined sewer overflows and industrial effluent, which threaten aquatic ecosystems and public health, will also become more critical. Green and gray infrastructure such as rain gardens, green roofs, and stormwater detention basins are being implemented to address these challenges and are further discussed in Section 4.8. Continued warming will further stress and reduce biodiversity in already‐stressed aquatic communities as thermal tolerance limits for various species are exceeded.

##### 3.7.2.3 All watersheds

As heavy precipitation events become more frequent, the threat of catastrophic flooding and dam failure will grow in riverine systems of all sizes, increasing the likelihood that aquatic communities and human populations will be displaced and invasive species will spread. While rivers need small floods to support functions such as habitat diversity and biological productivity, an increase in large‐magnitude floods can result in the loss of aquatic ecosystem services by increasing the occurrence of HABs.^298^ With warmer winter temperatures, mid‐winter thaws can result in ice jam flooding. These incidents have been increasing in the St. John River along the U.S.−Canadian border in Maine, resulting in replenished nutrients in floodplain soils but also deleterious effects from erosion of fish habitat.^323^

Changing climate conditions may also lead to changes in seasonal water levels and potential losses of population connectivity. For example, lower streamflow due to a reduction in spring snowmelt (or unusually early snowmelt) may impede the upstream migration of spawning fish species. Lower streamflow and warmer waters will alter nutrient loading and decomposition, which will in turn affect the structure and function of resident species assemblages as well as local food webs.^309^, ^324^ River and stream biota will also be subjected to changes in seasonal reproductive cues, affecting the growth rates of species populations and competitive interactions among resident species.^311^, ^314^, ^315^, ^325^ Of particular concern are those native species found in areas of high biological diversity (biodiversity hotspots). The largest concentration of hotspots in New York State is found along the Hudson River,^326^, ^327^ which is fed by all three watershed types. Complex interactions between downstream currents and ocean tides, which determine salinity levels and dispersal behaviors of aquatic organisms, make the Lower Hudson estuary biologically dynamic^328^ and particularly vulnerable to climate change. One positive effect of warming winter temperatures is that an increase in the number of ice‐free days could reduce the need for de‐icing salt, which has positive ecosystem benefits by reducing concentrations of de‐icing salt from runoff.^329^ Current salt levels in lakes and groundwaters indicate that polluted runoff from roadways is a chronic problem in many rural, urban, and agricultural watersheds across the state.^330^

#### 3.7.3 Regional variation

The impacts of climate change on riverine systems are expected to vary in different parts of the state. Four geographic features will play a primary role in influencing how watersheds respond to climate hazards such as changes in the frequency and intensity of precipitation events. The first is topographic variation. In New York State as elsewhere, watersheds with steeper catchment basins and narrower floodplains are prone to higher flow rates following precipitation.^331^ Earlier snowmelt and increases in extreme rainfall will lead to even greater episodic flow rates. A second attribute with similar consequences is land cover. In urban environments with large areas of impervious surface, and in environments with highly compacted soils, precipitation cannot seep into the ground but instead flows over the land and drains directly into rivers, leading to sudden surges in flow rates and increased potential for flooding. The confined channels of urban rivers will be increasingly challenged as the magnitude, frequency, and intensity of large precipitation events increase,^332^ heightening the need for adaptive management. A third consideration is latitude, which contributes to variations in climate trends, with northern regions undergoing disproportionately higher rates of average temperature change^333^ and greater changes in the length of cold seasons. The same is true of higher‐elevation areas. Many northerly locations in the state, such as the Adirondacks, also have steep slopes and greater topographic variation than other parts of the state, compounding future climate impacts on fluvial systems. The fourth feature of concern is proximity to major water bodies, where conditions are typically wetter under current climates—a situation that will increase in a more humid future. Marine and estuarine coastal riverine ecosystems will also face impacts associated with rising sea levels, which will shift saltwater intrusion upstream and impede drainage from low‐lying areas.^334^ Although the four cases described here focus on flood risks, seasonal droughts are also possible, particularly in urban and topographically variable watersheds where water retention could be limited during periods of low precipitation.

### 3.8 Marine and coastal ecosystems

BOX 9 **Takeaways**

- Rising water temperatures and changes to circulation will have cascading effects on the composition, range, and distribution of species in marine and coastal waters.
- Sea level rise will reduce the spatial extent and quality of existing coastal ecosystems.
- The effects of eutrophication, including local acidification and anoxia, will become more frequent and severe with higher temperatures and more terrestrial runoff.
- Impacts of climate change on marine and coastal ecosystems can be reduced with proactive ecosystem management that decreases runoff, protects shorelines, and improves habitat quality and connectivity.
- Increasing scientific monitoring that documents ongoing changes and model future changes will inform ecosystem management and regulatory response to ensure resilient coastal and marine ecological and human communities.

#### 3.8.1 Description and importance

New York State has more than 2600 miles of marine and estuarine coastline, a resource that is ecologically, economically, and culturally important.^335^ Coastal ecosystems are dynamic and diverse, hosting multiple habitat types (e.g., tidal wetlands, submerged aquatic vegetation, beaches and dunes, pelagic and benthic habitats) and numerous species (e.g., finfish, shellfish, zoo‐ and phytoplankton, seagrasses, algae) that provide essential services to the people of New York, such as minimizing coastal flood damage.^336^ Many of these ecosystems are already responding to climate hazards such as warming water temperatures and rising sea level,^337^, ^338^, ^339^ as well as to land‐use stressors such as nutrient runoff and infrastructure development. As current climate trends continue, climate and nonclimate stressors will likely increase, with varying impacts depending on ecosystem type and location.

#### 3.8.2 Impacts and risks

Five climate hazards have widespread and intersecting impacts on marine and coastal ecosystems:

- Increases in water temperature, driven by both atmospheric warming and changes to ocean circulation, will alter habitat extent and quality as well as species survival and distribution. Some of these impacts are already being observed.
- Increases in precipitation will alter water chemistry (salinity, acidification, oxygenation), altering habitat extent and quality and species distributions.
- Sea level rise will increase coastal erosion, reduce the spatial extent of coastal habitats such as salt marshes, and magnify the impacts of storm surge and higher tidal reach, leading to more frequent and intense flooding and terrestrial pollutant runoff.
- Changes in ocean carbon chemistry from the absorption of carbon dioxide will exacerbate already‐occurring local acidification in coastal habitats from terrestrial nutrient inputs, resulting in reduced survival of many plant and animal species.
- Ocean deoxygenation from increased temperatures and nutrient runoff will reduce the habitability of waters and the health of species.

Some of these factors, such as water temperature, will have a greater impact in New York than in other areas due to ocean circulation patterns and the predominance of coldwater species in the state's coastal waters. These effects are already manifesting. Factors such as changes in carbon chemistry and ocean acidification may not impact New York State waters in the near‐term but could in the future. The specific drivers and impacts of these factors are detailed in the following sections.

##### 3.8.2.1 Water temperature

Trend analyses indicate that surface water temperatures in the ocean near New York State have been increasing at a rate of 0.5−0.7°F per decade.^162^ Two primary factors drive this trend: increasing atmospheric air temperatures and changes in ocean circulation patterns. Model projections show that air temperatures in the state's coastal regions will increase by an additional 3.8−6.1°F by the 2050s, relative to the 1981−2010 average.^340^ This will result in additional increases in water temperature. Water temperatures off the coast are largely modulated by the Atlantic Ocean circulation, which brings cold water from Greenland's melting ice to the New York region. Numerous studies have documented already‐notable changes in the speed and location of the Atlantic circulation.^227^, ^337^, ^341^, ^342^, ^343^ As climate change shifts circulation patterns, a higher proportion of warm Atlantic water is reaching New York State's coast, compounding the warming from increased atmospheric temperature. In addition to the long‐term warming of coastal waters, marine heat waves—periods of days to months when ocean temperatures increase well beyond average seasonal conditions—are becoming more frequent and longer‐lasting globally. This pattern is expected to increase by more than an order of magnitude by the late 21st century.^344^

These changes have far‐reaching consequences. One easily observed consequence is the effect on seasonal weather patterns—for example, the well‐documented trend of a shortened cold season and a lengthened warm season, which is likely to continue.^337^, ^345^, ^346^ Two other notable effects of temperature change are the loss of the summer 20°C isotherm (the boundary where surface waters above and below 20°C meet) and the shrinking of the “cold pool.” Historically stable off the New York coast, this isotherm has now shifted north of the New York Bight during summer months due to climate warming. As waters continue to warm, the isotherm will be north of the New York Bight during autumn as well.^337^ The cold pool is a body of cold bottom water that develops in the spring and is maintained by northerly currents off the coast of New York during the summer and autumn months. The cold pool serves as a critical source of nutrients, food, and refuge for many species in the New York Bight. Studies have shown that the geographic extent and seasonal duration of the cold pool are rapidly decreasing due to changes in ocean circulation (though climate‐related increases in wind and ocean mixing could also be contributing factors).^337^, ^341^, ^347^

Because of these changes in water temperature off New York's coast and across the Northeast Shelf, some researchers consider these “among the fastest warming ecosystems worldwide.”^346^ Warming is already impacting some species in the state's coastal waters.^337^, ^345^, ^348^ Fish species are particularly sensitive to changes in water temperature for two reasons: fish are unable to modify their internal temperature (i.e., ectothermic), and they get their oxygen directly from water (dissolved oxygen is inversely related to water temperature).^345^ A warming ocean increases metabolic demand while simultaneously decreasing available oxygen.^349^ To maintain optimum metabolic conditions, fish must move to their ideal temperature habitat. As temperatures warm, fish in the northern hemisphere can either migrate northward or relocate into deeper water to reach colder temperatures. For example, over the past century, the distribution of cod in the North Sea has shifted to the north and into deeper habitat in response to warming waters.^345^ A survey of Long Island Sound that examined changes in community composition from 1984 to 2008^351^ demonstrated that warming water temperatures are displacing coldwater species northward, and also that species historically found south of New York's waters are now increasingly populating the Sound. In the New York−New Jersey Harbor Estuary, numbers of tomcod, perch, hogchoker, white catfish, striped bass, blueback herring, shad, and eel have all decreased due to a combination of rising temperatures, fishing, and habitat pressure.^351^

Marine mammals, in contrast to fish and many other marine organisms, are more adaptive to increases in water temperature as their internal temperature and oxygen use is not water‐dependent. However, marine mammals could feel the indirect effects of climate change as their prey and habitat move northward and deeper.^345^ In the New York Bight, changes in abundance and relative composition of bottom‐dwelling organisms and fish‐eating organisms over the past 60 years have been interpreted as indicating “major changes in the food web.”^337^ There is evidence that *Calanus finmarchicus*, an ecologically important copepod in the New York Bight, is declining in response to warming temperatures,^337^ which may affect the presence of the North Atlantic right whale. Other important nonfish species, particularly shellfish, are also likely to shift in response to warming, influencing the many organisms that consume them. For example, Atlantic surf clams in the Delaware‐to‐Virginia area have moved to deeper waters since the early 1980s in response to warming temperatures.^352^ This has changed the locations of their fisheries and made them potentially less accessible to their predators.

The temperature‐driven species shifts that occur as the climate warms, while problematic for existing fisheries and species compositions, could present opportunities for New York State in the future. Although historical fisheries may be lost, new fisheries could arise, along with new economic and cultural opportunities. The Shifts in Lobster and Crab Populations case study provides an example of this effect, but there are other examples as well. For instance, while winter flounder has been declining sharply due to increased temperatures,^338^ summer flounder (fluke) and black sea bass have increased in abundance over the past decade, likely in response to warming water temperatures.^337^ A synthesis of bottom trawl surveys from the New York Bight identified an overall increase in warmwater species since the 1960s.^337^ Moreover, a study of 82 species in the Northeast Shelf indicated that while many species (including the Atlantic sea scallop, Atlantic cod, Atlantic mackerel, and many bottom‐dwelling organisms) will be negatively affected by increased temperatures, 17% of species are likely to benefit from warming waters.^353^ These include inshore longfin squid, butterfish, and Atlantic croaker. The Atlantic States Marine Fisheries Commission, New York‐New Jersey Harbor and Estuary Program, and New York Ocean Action Plan have all compiled lists of species of concern and noted expected changes for important species in the state's waters.

Besides increasing metabolic stress and altering food availability, temperature changes will affect species survival in New York's coastal waters in other ways. Changes in seasonal temperatures affect species life cycles, mating and development, interactions between predator and prey species, and other ecosystem interactions.^345^ For example, recent declines in winter flounder could be largely driven by changes in predator−prey interactions caused by warming temperatures. Winter flounder larvae have historically found refuge for development in cold winter waters, but with sand shrimp becoming more active in the winter due to warmer temperatures, shrimp consumption of flounder larvae has increased.^338^ Temperature‐induced changes in black sea bass populations have led to concerns that these fish are increasingly eating lobster and other crustacean larvae.^354^

##### 3.8.2.2 Precipitation

Increased precipitation in the state will have downstream impacts on water quality and habitats in marine and coastal waters.^355^ With more precipitation on average, as well as a rise in the frequency, intensity, and duration of extreme precipitation events, there will be an increase in runoff of land‐derived pollutants, nutrients, and sediments into coastal waters. These inputs are already at levels detrimental to habitat and water quality,^356^ and greater inputs from increased precipitation will exacerbate impacts. Large storms that affect the watersheds of river‐connected estuaries can result in temporary decreases in salinity, which can have adverse impacts on fauna that require higher salinity levels.^357^ Increased sediment supply could allow coastal habitats to keep pace with sea level rise, preventing effective drowning, but would also limit visibility and light penetration, potentially reducing biological productivity.

##### 3.8.2.3 Sea level rise

While several factors contribute to sea level rise at any single location, climate change is the major driver of contemporary sea level rise via the melting of glacial ice and thermal expansion.^358^ New York's coasts are experiencing rates of sea level rise faster than the global mean.^338^, ^359^ At Manhattan Island, sea level has risen at a rate of about 1.6 inches per decade over the past 40 years, relative to local fixed reference on land.^162^ Similar rates have been observed at other locations in the New York−New Jersey Harbor Estuary, such as Sandy Hook.^338^ Such rates of sea level rise have major impacts on habitat distribution and area, severity and extent of storm influence, and populations of organisms, from reefs and grasses to birds and humans.^360^

Coastal wetlands and marshes are at particular risk from rising sea levels. These ecosystems depend on tidal flooding yet are sensitive to both lateral and vertical changes in the extent of flooding. As sea levels rise, high tides encroach farther inland and low tides recede less. Under natural conditions, marshes would migrate inland as sea levels rise, maintaining an ideal location for tidal inundation. However, marshes may be unable to migrate at the pace of future sea level rise and could also encounter built features that prevent migration.^361^, ^362^ Other factors that reduce the capacity of marshes to maintain equilibrium with rising sea levels include erosion from increased storm frequency and strength, land‐use change for development, and reduced sedimentation of coasts from dredging and watershed disturbance. Decreased wetland plant species richness is another common outcome of sea level rise.^363^ Climate‐induced changes in plant and animal communities can also destabilize marsh sediments, speeding erosion and habitat loss.^364^ If marshes are drowned and reduced, there will be a considerable loss of critical habitat for many important shellfish, finfish, bird, and mammal species. Moreover, the loss of marsh area will cause more contaminants to enter the ocean, further exacerbating eutrophication (refer to BOX 10) and acidification. Seagrasses and other aquatic vegetation in coastal wetlands and marshes also play an important role in carbon sequestration. With the loss and degradation of these ecosystems due to sea level rise, marshes could transition from a carbon sink to a carbon source.

BOX 10 **Harmful algal blooms and eutrophication—How land use affects water quality and ecosystems**

A major impact of land use across freshwater and marine ecosystems, potentially exacerbated by climate change, is the presence and persistence of harmful algal blooms (HABs) and eutrophication. These occur when excess nutrients from land enter waters, promoting algal growth and leading to harmful outcomes for ecosystems and water quality.

In the case of HABs, some algae release chemicals into the water that are toxic to humans and animals (refer to the Harmful Algal Blooms case study for more information). During the process of eutrophication, algae decompose and consume oxygen in the water, which makes the water no longer habitable for many animals (Figure 5‐11). This decomposition process also produces greenhouse gases (carbon dioxide and methane) and contributes to acidification. Other downstream consequences of these phenomena are described throughout this chapter.

HABs such as red tides are increasing during the summer months in New York State's coastal waters due to warming temperatures, and HABs are predicted to increase in frequency as temperatures rise.^37^ The increasing prevalence of HABs and eutrophication indicate that detrimental nutrient delivery to waters from land is a growing problem. Practices that can reduce nutrient runoff include reduced fertilizer use on agricultural land, prevention of erosion and runoff from developed land, and improved infrastructure to reduce stormwater and wastewater runoff. Such practices can greatly reduce stress to ecosystems, which can improve ecosystem resilience to climate change.

**FIGURE 5‐11:**
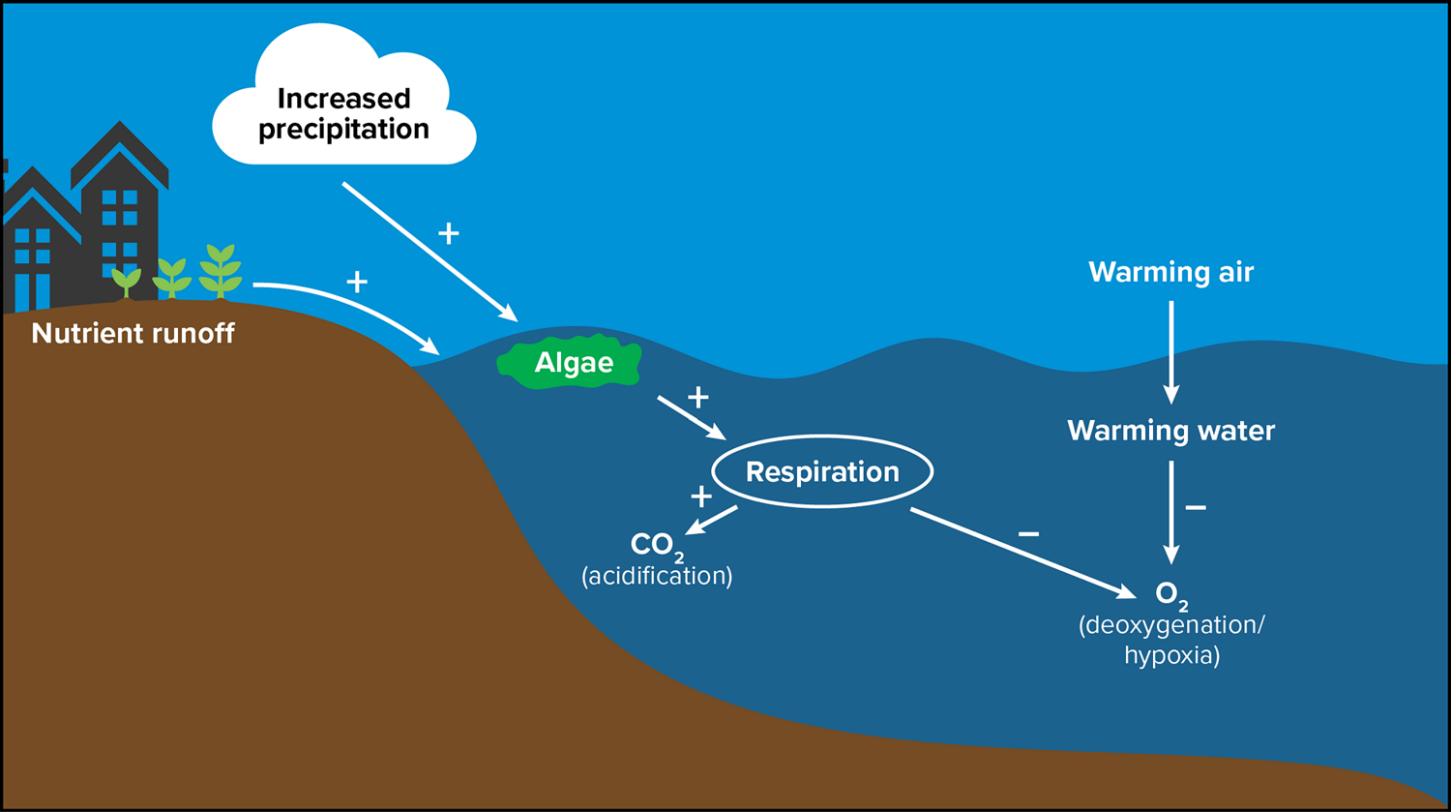
The eutrophication process. Figure adapted from an illustration by assessment team members F. G. Boudinot and R. L. Shuford.

Even as some marsh habitats are lost to climate change, new tidal marsh habitats could be created as waters extend laterally along floodplains with sea level rise.^297^ Marsh expansion depends on sufficient sediment supply and effective land management. This represents an opportunity for the generation of new wetlands to compensate for or even exceed projected losses.^297^ Any aggregate expansion of marsh area could result in an increase in tidal wetland‐derived ecosystem services.

Estuarine ecosystems are also highly sensitive to sea level rise. These systems represent the boundary between salt (ocean) and fresh (river) waters. As sea levels rise, estuarine habitats migrate upward and inland. The resulting inland flux of saline waters can affect the distribution of species and habitats, with those more tolerant of salt water moving upstream.^365^

Coastal groundwater sources will become more saline as sea level rise increases the underground infiltration of saline waters. Saltwater intrusion into groundwater will affect plant communities inshore that are not adapted to saline water. Coastal forests and wetlands host plants that are adapted to saline groundwaters, yet those habitats have been rapidly razed for development over the past several decades in New York State at a rate of 47−122 acres per year.^351^ Freshwater wetlands that are upland of and adjacent to coastal wetlands will be negatively impacted by saline groundwater intrusion and more extensive storm surge.^296^ While this will negatively affect freshwater‐adapted wetland and forest species, some studies indicate that salinization of freshwater wetlands will also reduce their rate of methane emissions.^366^, ^367^ Because wetlands are the dominant natural source of methane,^368^ such an effect could reduce the state's overall greenhouse gas emissions. Saltwater intrusion into groundwaters in coastal areas will also affect drinking water, posing new health risks that will require infrastructure changes.^369^, ^370^ Refer to the Water Resources chapter for more information on the effects of saltwater intrusion on drinking water supplies.

Sea level rise will also amplify the magnitude and damaging effects of storm surges, which could affect many ecosystems. Climate change is expected to increase the frequency and intensity of storms along the coast.^371^ For example, a coastal flood that currently occurs every 100 years in New York City is expected to occur every 24 years by 2050 under the SSP2‐4.5 climate scenario, and at Montauk, the same flood is expected to occur every 3 years.^372^ The influence of rising sea level will cause storm surges to reach farther inland and will increase the volume of floodwater, exacerbating impacts on ecosystem dynamics on land and in coastal waters. Water in New York State's estuaries, for example, is already contaminated by bacteria (e.g., *Enterococcus*) from sewage overflow. Inundation of land during extreme storm surges will cause such contamination to increase.^351^ Storm surges will also remobilize on‐land sediments contaminated with metals, plastics, and mercury, increasing levels of these contaminants in coastal waters. Research has shown that, as environmental temperatures rise, fish and other organisms accumulate these toxins at higher rates.^373^, ^374^ Communities in coastal areas will also experience impacts from increased pollution related to storm surges, raising concerns about environmental justice.^374^, ^375^ Such impacts have already been observed. According to the New York City Department of City Planning, 30% of all open industrial facilities in the city are within the 100‐year floodplain indicated on the Federal Emergency Management Agency's preliminary work maps, yet 60% flooded during Superstorm Sandy, contributing to environmental contamination during that event.^376^ Increases in the frequency and magnitude of storm surges can lead to coastal erosion and loss of coastal wetland, beach dune, and barrier island habitats. These habitats serve as natural buffers for coastal communities, protecting them from wave action and flooding, removing pollutants and nutrients from runoff, and serving as carbon sinks.

##### 3.8.2.4 Compounding effects: Acidification, deoxygenation, and living habitats

###### Acidification

The global oceans have absorbed 20%−30% of the carbon dioxide emitted to the atmosphere by anthropogenic activity in the last two decades.^377^ This addition of carbon dioxide alters the oceans’ carbon chemistry. Reductions in pH, a process referred to as ocean acidification, make it harder for organisms to create shells and affect other physiological processes. In some parts of the global ocean, such as upwelling regions in the Pacific, ocean circulation patterns make acidification particularly problematic for marine organisms.^378^, ^379^ The particular conditions and ocean circulation of the Atlantic could lead to a loss of calcium carbonate saturation in the coming decades, which will negatively affect many species.^380^

Localized acidification occurs periodically in some coastal areas of New York State due to eutrophication or reduced salinity associated with greater freshwater runoff. During eutrophication, algal blooms raise pH during photosynthesis; pH declines during respiration as carbon dioxide is released.^381^, ^382^, ^383^, ^384^ Stratified coastal waters may show higher pH in the shallow photic zone and lower pH at depth where respiration dominates.^383^ Waters with high inputs of fresh water have lower salinity and alkalinity, which also changes carbon chemistry and drives acidification.^382^ Increases in precipitation in coastal New York State will further exacerbate acidification, partly by reducing salinity and partly by increasing nutrient delivery (thus driving eutrophication). Measurements of pH taken from 2007 through 2021 indicated that the areas most affected by acidification were the western Long Island Sound and the New York−New Jersey Harbor.^341^ Those locations have historically hosted Atlantic surf clams, which are negatively affected by acidification.^385^ In addition, Long Island Sound and Jamaica Bay have shown persistent acidification for up to 40 days in response to brief algal blooms,^383^ highlighting the long‐term impact that eutrophication can have on carbon chemistry.

Locations with eutrophication and high freshwater input that today experience acidification are vulnerable to increasing acidification from climate change, which could have a major negative impact on important finfish and shellfish. Oysters and other mollusks have been classified as very highly vulnerable to climate change because of their need for stable carbon chemistry to create shells and because they lack the ability to migrate when carbon chemistry is not ideal.^353^, ^380^, ^386^ Evidence suggests that increased carbon dioxide concentrations in marine waters have contributed to reductions in the quantity and quality of shellfish.^387^ Changes in ocean carbon chemistry and acidification can also have negative effects on the growth and development of finfish,^381^ including summer flounder.^388^ Future changes in carbon chemistry from increases in carbon dioxide and precipitation could further affect finfish and shellfish populations in the state.

###### Deoxygenation

Cold water holds more oxygen than warm water. Thus, concentrations of dissolved oxygen in ocean waters will decrease as temperatures rise. Changes in circulation could reduce vertical mixing, further reducing dissolved oxygen.^389^ Meanwhile, higher nutrient input from sea level rise, increased precipitation, and storm surge could heighten the prevalence and extent of eutrophication,^390^, ^391^, ^392^ which also reduces available oxygen. Because many marine organisms require oxygen to consume food and produce energy, deoxygenation is a primary ecological concern, both today and under future climate change scenarios.^393^ In some scenarios, deoxygenation can lead to anoxia and hydrogen sulfide production, generating toxic impacts on affected species. Paleoclimate evidence indicates that climate change events in Earth's history occurring at slower rates than today occasionally created sufficient ocean deoxygenation to generate widespread euxinia (water with no oxygen and elevated hydrogen sulfide),^394^ which highlights the potential for substantial future impacts if deoxygenation becomes widespread.

Locations most susceptible to deoxygenation are coastal areas with high nutrient input, as well as areas with deep channels such as Long Island Sound, Jamaica Bay, and the Hackensack and lower Passaic rivers in New Jersey.^338^ The deeper channels already experience low oxygen^338^ and will be subject to more intense anoxia as temperatures warm. In Long Island Sound and Jamaica Bay, deoxygenation from eutrophication has been shown to persist for over a month in response to brief algal blooms, highlighting the susceptibility of those locations to short‐lived events.^383^ While mobile species may be able to move to avoid the stresses of deoxygenation, less mobile species are more vulnerable to such impacts. Already, eutrophication is contributing to the decline of eelgrasses and horseshoe crabs in New York waters.^351^ Bivalves have been shown to be sensitive to both acidification and deoxygenation, and are particularly susceptible to the combination of lowered pH and dissolved oxygen levels that can result from eutrophication and climate change.^381^ In the New York−New Jersey Harbor Estuary, dissolved oxygen has actually been increasing since 1950 in response to reduced sewage inputs under the Clean Water Act,^351^ which reveals the importance and success of on‐land eutrophication reduction efforts.

###### The special case of living habitats

Some marine and estuarine habitats are essentially organisms themselves. For example, oyster reefs and marsh grasses serve as habitat, refuges, and feeding grounds for a wide range of organisms, from fish to mammals to birds. Some scientists have postulated that these living habitats are the most susceptible to climate change because of the multiple interactions they have with various species (including predation) and physical conditions such as temperature, sea level, and water chemistry.^360^ Oyster beds are vulnerable to climate‐related factors such as increases in predation, disease increases, and changes in water quality. The spike in blue crab populations in New York State's coastal areas brought on by warming waters has led to increased predation pressure on oysters, because the crabs eat oysters and other bivalves^338^ (refer to the Shifts in Lobster and Crab Populations case study for more information). Temperature‐driven increases in disease provide another source of stress,^395^, ^396^, ^397^ with parasites such as MSX and Dermo becoming increasingly prevalent as winter temperatures grow warmer.^338^ These temperature‐related stressors could be compounded by changes in water quality, water carbonate chemistry, and oxygen availability.^380^ Oysters improve water quality by filtering out contaminants; as a result, they are vulnerable to toxic levels of contaminants^398^, ^399^ and accumulate contaminants in their tissues.^400^ Because they create carbonate shells, oysters are particularly stressed by acidification.^380^ Oysters are often found in coastal regions with high levels of organic matter that are vulnerable to eutrophication, so they are susceptible to metabolic stress from deoxygenation as well.^381^ Finally, sea level rise poses a long‐term threat to oysters, as deep waters restrict their growth and their ability to migrate is limited.

Some living habitats have already been harmed by rising temperatures. Eelgrass, for example, serves as an important habitat for summer flounder and blue crab. Both species are increasing in New York waters due to rising temperatures and may present new fishery opportunities. However, growth in blue crab and summer flounder populations could be stymied by the lack of eelgrass habitat. Existing restoration efforts in the state, including in the New York−New Jersey Harbor Estuary and Jamaica Bay, have been largely unsuccessful due to the stress exerted on eelgrass by high water temperatures.^338^ Other climate hazards affecting eelgrass include eutrophication, extreme storm events, and sea level rise. Algal blooms that contribute to eutrophication can restrict light from reaching eelgrass beds, preventing their growth and leading to their demise.^351^ Storm events can break up, dissipate, and destroy eelgrass beds, with reestablishment taking up to several years.^351^ Sea level rise will compound these issues by moving habitat location, or even outpacing habitat migration rates to the point of habitat loss.^401^ Nonclimate land‐use stressors can also compound these effects, with nutrient and pollution runoff exacerbating temperature‐ and carbon‐related reductions in water and habitat quality. Land‐use change and development can reduce the capacity of habitats to migrate in response to sea level rise and temperature change.

#### 3.8.3 Regional variation

Six geographic zones constitute most of New York State's coastal and marine ecosystems (Figure 5‐12).^390^ While region‐specific studies are not available for each of these geographic zones, the following generalized regional variability for each can be inferred based on impacts by ecosystem type as described in Section 3.8.2:

- **Zone 1: The marine ecosystem from Long Island's southern coastline to deeper waters 70**−**100 miles offshore**. This region is experiencing observable changes in temperature that are affecting species composition and seasonal variability.^337^ Deoxygenation, carbon chemistry changes, and salinity changes are less pronounced here than in other coastal regions. Changes in the frequency and magnitude of storms increase the vulnerability of dunes and beaches on Long Island's southern coastline, with storm surge impacts further exacerbated by sea level rise.
- **Zone 2: The tidal wetlands and lagoonal bays on the southern side of Long Island, from New York City to Montauk**. This area is experiencing deoxygenation, largely from nutrient inputs, which combine with increasing temperatures to drive HABs and fish die‐offs.^402^, ^403^, ^404^, ^405^ The Shinnecock Nation has experienced reduced shellfish harvests due to declines in water quality, which may be exacerbated by warming and carbon chemistry changes associated with climate change.^406^ Projected increases in precipitation will further increase damaging nutrient inputs and associated eutrophication. Combined with other stressors like sea level rise and changing sedimentation,^361^, ^407^, ^408^ coastal wetland habitat loss will continue to be pronounced in this region.^339^ Some storm events have breached barrier islands, worsening water quality changes of these otherwise isolated lagoons.^339^ Some storm events have breached barrier islands, changing the water quality of otherwise isolated lagoons.^409^, ^410^ The Shinnecock Nation and other communities are already losing land and experiencing increased flooding due to storm surge and sea level rise.^406^ Increasing salinity from sea level rise impacts both surface (lagoon) and groundwater resources.
- **Zone 3: The tidal wetlands and estuarine ecosystems in Long Island Sound**. Warming temperatures could combine with a rise in nutrient inputs from increased precipitation to amplify the extent and duration of deoxygenation in this region. Habitat loss from sea level rise and storm surge will accelerate. Changes in species composition caused by temperature increases are already being observed, with lobster populations declining in recent years.
- **Zone 4: The tidal wetlands and estuarine ecosystems in the Peconic Estuary system at the east end of Long Island**. Increased temperatures and nutrient inputs are causing more frequent HABs, with associated deoxygenation and carbon chemistry changes. This pattern could worsen as temperatures rise further and precipitation increases nutrient inputs. Species composition is shifting in response to temperature changes, with species such as bay scallops suffering from warming waters.
- **Zone 5: The New York**−**New Jersey Harbor Estuary at Long Island's west end and the shores of New York City**. Increased precipitation will cause an upsurge in pollution from runoff of urban and terrestrial contaminants. Warming temperatures are already changing the composition of species, and that trend is expected to continue. With sea level rising and storm surges increasing in intensity and frequency, flooding and erosion of marsh habitats will reduce habitat area and associated biodiversity.
- **Zone 6: The tidal wetlands and waters of the Hudson River estuary from the harbor up the Hudson River to the Troy Dam**. Salinity gradients in this zone could change with sea level rise and increased precipitation. Habitat loss from sea level rise and storm surge could worsen. Flooding and erosion of marsh habitats will reduce habitat area and associated biodiversity. Some new tidal marsh area could develop as water levels rise.

**FIGURE 5‐12:**
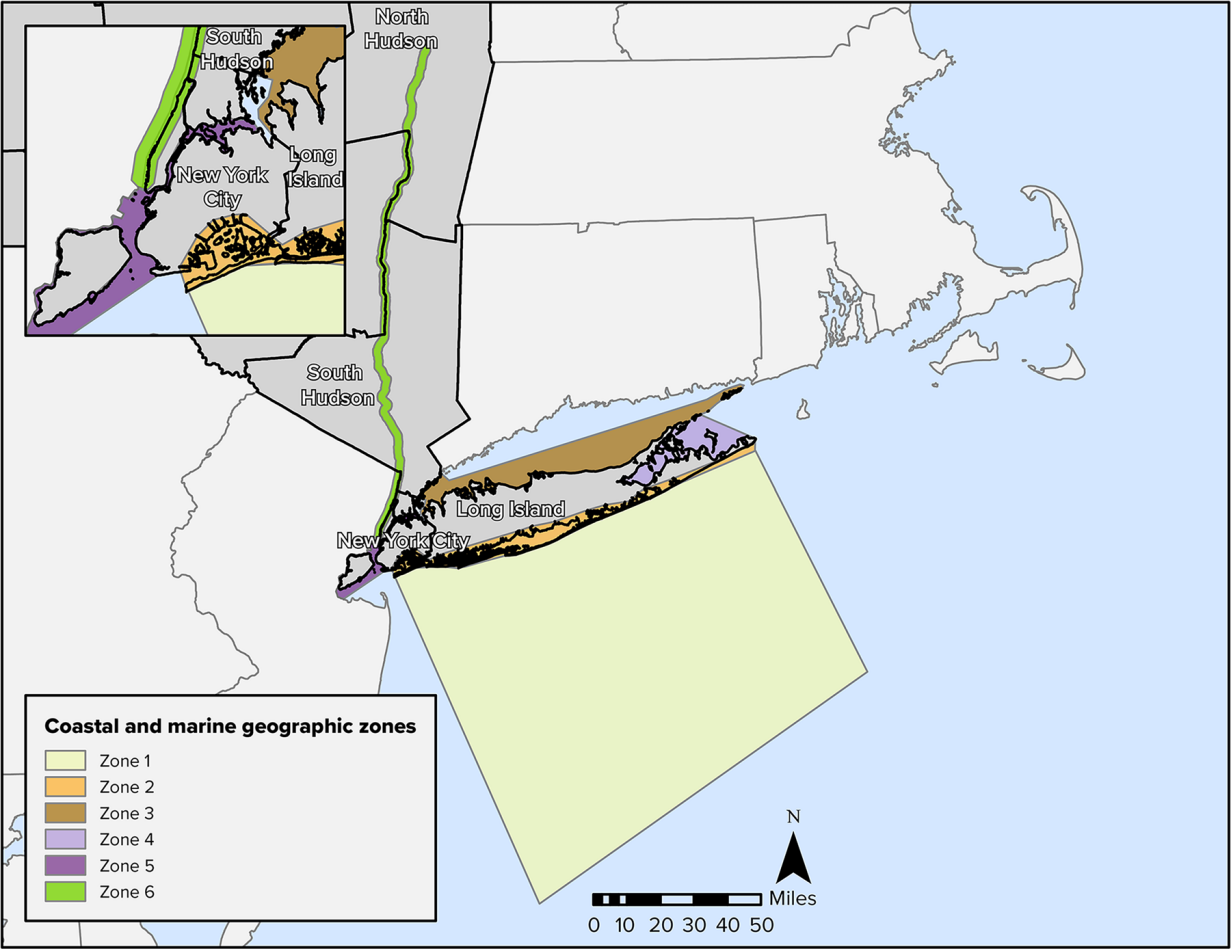
Six geographic zones constitute New York's coastal and marine ecosystems. *Data from U.S. Geological* Survey (2023)^411^ and New York State Department of State (2020).^412^

### 3.9 Cross‐cutting ecosystem topics

This section looks at climate impacts on urban ecosystems, Indigenous lands, native flora and fauna, and invasive species. These topics span many of the ecosystem types discussed above.

#### 3.9.1 Urban ecosystems

BOX 11 **Takeaways**

- Based on projected changes in climatic conditions, impacts already being observed in urban areas will increase in severity and duration, adding impetus to enhance urban ecosystem properties that protect natural habitats and human well‐being.
- Urban areas are complex socioeconomic−ecological systems, with strong interdependence between human activities and ecosystem properties.
- Most of New York State's urban areas include substantial marine or freshwater coastal ecosystems, which provide critical support for human communities.
- The benefits of functioning urban ecosystems include the cooling effects of trees and other vegetation, but those benefits are unequally distributed and disproportionately favor higher‐income neighborhoods.

##### 3.9.1.1 Description and importance

Urban ecosystems are of great importance in New York State. Approximately 88% of state residents^413^ live in urban areas, a number that is expected to increase in the future. New York City is the largest urban area in the United States. The metropolitan areas of Buffalo and Rochester each have more than 1 million people. New York State has three additional metropolitan areas with more than half a million residents, and seven more with more than 50,000 residents.^414^ Climate change is expected to have multiple impacts on ecosystems in urban areas.^415^ New York City alone includes 520 miles of coastline and has high vulnerability to storm surge, which is expected to increase in severity as climate change raises sea level in the future.^162^ The cities of Buffalo and Rochester are located adjacent to Lake Erie and Lake Ontario, respectively, and are influenced by lake conditions and lake‐effect weather patterns.

Urban areas are complex socioeconomic−ecological systems whose components interact dynamically across varied temporal and spatial scales, with humans, their activities, and associated infrastructure as important drivers.^416^ The study of urban ecosystems typically considers the central city, suburbs, and exurbs, including outlying regions that are linked to cities through the exchange of materials and energy. Urban ecosystems include most of the ecosystem types discussed in this assessment, such as forests, wetlands, lakes, rivers, and grasslands. However, in urban settings, humans act as a stronger driving force than they do in rural and natural settings where these same ecosystems are present. Increasingly, planning for the design and maintenance of urban green infrastructure is informed by principles of ecological science with an aim of maximizing multiple ecosystem services, among which resilience to climate change is of increasing prominence.^417^ Integrating traditional ecosystem science approaches with those of socioeconomic‐based ecosystem planning has been posed as a central challenge to developing an interdisciplinary systems science with sustainability as a foundation.^418^

Consideration of climate change impacts on the state's urban areas also raises questions of equity. For example, as average summer temperatures increase, heat mitigation from trees and greenspaces becomes a critical ecosystem service for city residents. Urban trees provide localized and regional cooling through shading and transpiration, and they also reduce air pollution and provide carbon sequestration.^419^ However, these benefits are often not equally distributed. Low‐income areas of cities often have less access to urban green infrastructure such as street trees, parks, and stormwater detention basins that help lower exposure to flooding.^420^, ^421^ Research within New York City reinforces the finding that access to urban green infrastructure is uneven and unequal based on race and income.^422^ Yet, efforts to create more greenspace in low‐income neighborhoods can precipitate gentrification, leading to the displacement of longtime community residents.^420^ These findings highlight the importance of prioritizing environmental justice concerns when considering adaptation strategies for climate change in urban areas.

##### 3.9.1.2 Impacts and risks

The urban heat island effect, in which air temperatures in cities are warmer than those in surrounding rural areas, is a widespread and well‐recognized global phenomenon.^423^ In New York City, the heat island intensity (temperature differential between urban and adjacent rural areas) averages about 3−5°F but shows high spatial and temporal variability.^424^ The cities of Buffalo and Syracuse show similar heat island intensity, which can exceed 10°F during summer.^425^ Urban areas also cause other localized climatic patterns such as diminished wind speeds^424^ and increased cloudiness and precipitation.^426^ The existence of the urban heat island and related climatic effects has facilitated studies of plants and insects that have provided insights into expected ecosystem responses to climate change by assuming that the rural‐to‐urban increase in temperature can serve as a model for how future climate warming will affect these organisms.^427^

Urban areas generally have less vegetation than surrounding rural areas due to the replacement of native plants by structures and roads. However, studies have shown that urban vegetation can have enhanced productivity and growth rates compared with vegetation in nearby rural areas due to factors such as warmer temperatures and higher carbon dioxide concentrations.^428^ This pattern has been observed in New York City and other cities.^429^ However, trees and forests in urban areas face greater risks from drought, invasive pests, pollution, and extreme heat than those in rural areas, which highlights the increased challenges involved in managing urban trees.^430^ Urban trees are important to preserve as they provide many ecosystem services, including localized cooling (Figure 5‐13).

**FIGURE 5‐13:**
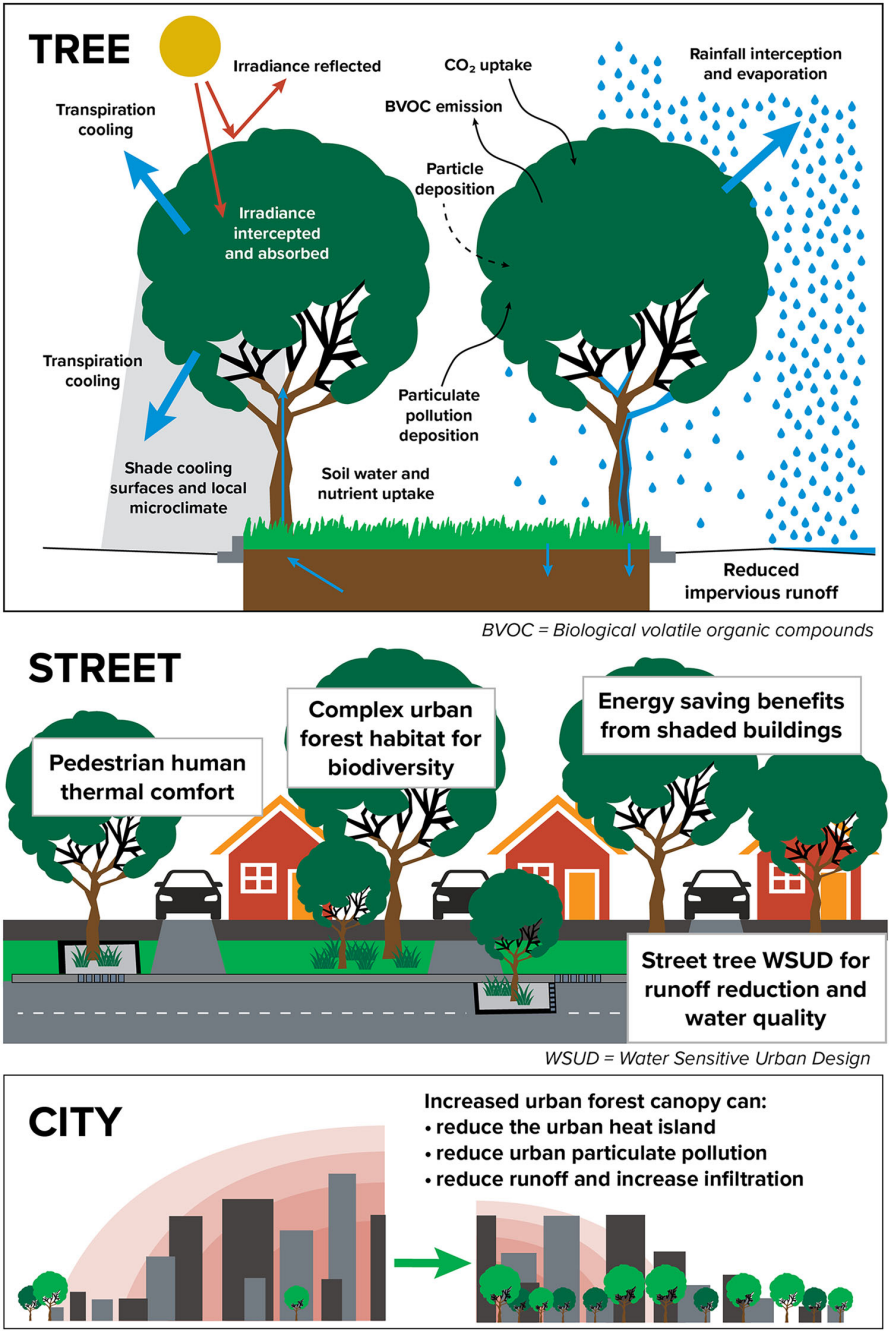
Urban forest ecosystem services and functions at the tree, street, and city scales. Figure adapted from Livesley et al.,^71^ licensed under CC BY 4.0.

Impermeable surfaces that accelerate runoff and other human activities that affect water quality heavily impact urban aquatic ecosystems and hydrology.^322^ Water quality investigations have shown increased levels of nutrients, sediment, trace metals, and trace organic pollutants in urban lakes, rivers, wetlands, and groundwater relative to similar regional waters in natural landscape settings.^431^ Management approaches to urban water quality include stormwater detention ponds and basins,^432^ constructed wetlands, and riparian ecosystem restoration.^433^ An increase in the frequency of large storms associated with climate change has exacerbated water quality concerns and related aquatic ecosystem responses such as HABs.^434^ Impermeable surfaces result in rapid runoff of precipitation, limiting groundwater infiltration and resulting in higher‐than‐natural storm runoff.^435^ This rapid runoff can cause deep erosion of stream channels that disconnects streamflow from adjacent groundwater, which limits natural ecosystem processes such as denitrification (removal of nitrate from solution).^321^ Management practices such as stormwater detention and the use of permeable pavement can help minimize rapid urban runoff and its associated nutrients and sediment.^436^ Increases in water temperature driven by runoff from hot pavement are another impact of impermeable surfaces—one that can have deleterious effects on fish and aquatic insects.^437^ Ongoing and projected future increases in air temperature and the frequency of large storms will pose continuing challenges for the management of urban water resources and aquatic ecosystems in the 21st century.^438^

##### 3.9.1.3 Regional variations

New York State's urban ecosystems are widely distributed, but the most populous cities are concentrated in marine and estuarine coastal zones or near freshwater lakes. Regional differences in the ways that climate change affects these urban ecosystems will be determined in large part by how climate change affects these adjacent aquatic systems. To the southeast, cities from the South Hudson to Long Island assessment regions will face continuous sea level rise with increasing flood hazards and saltwater intrusion. Cities in the Great Lakes assessment region, whose climates are modified by lake effects, will face new challenges associated with the warming of lake waters, which can add more energy and precipitation to storm events.

#### 3.9.2 Indigenous lands

BOX 12 **Takeaways**

- Climate change is exacerbating existing environmental justice issues that affect Indigenous Nations’ lands and people.
- Climate change negatively impacts flora and fauna that are culturally significant to Indigenous Nations.
- Indigenous Nations are actively engaged in climate change mitigation research and policy for their communities.

##### 3.9.2.1 Description and importance

Prior to the arrival of non‐Indigenous people beginning in the 17th century,^439^ the area that is now New York State was home to more than a dozen Indigenous Nations.^440^ The Assessment Introduction introduces these Indigenous Peoples and their lands, which are quite diverse throughout the state both in terms of their ecosystems and their historical and political contexts. The territories held by federally recognized or state‐recognized^440^ Indigenous Nations located in New York total 137 square miles (87,650 acres)^76^ and are located across the state, from the eastern part of Long Island to Lake Erie and north of the Adirondacks. The borders defining these territories continue to change due to purchases and donations of land and past and ongoing legal actions over land claims. Additionally, New York City is host to the largest urban population of Native Americans in the country. Relocated Algonquin Nations maintain a connection to their homelands. The Stockbridge‐Munsee Band of the Mohican Nation recently had one of their sacred sites, the Papscanee Island Nature Preserve, returned to them,^441^ and other parcels of land of cultural significance to Indigenous Nations have been returned through land trusts or private donations.^441^, ^442^

In New York, approximately 54% of the total land area in Indigenous territories is covered by forest, while grass/shrub ecosystems make up less than 2% of land cover—figures that are consistent with land cover patterns statewide. However, as shown in Table 5‐1, Indigenous‐managed lands are especially rich in lakes, ponds, and wetlands, and have less developed and agricultural land. The Akwesasne and Allegany territories have more than 10% lake and pond land cover, and the Tonawanda, Akwesasne, and Tuscarora territories each include more than 30% wetland land cover.^76^ Additionally, the Shinnecock Reservation has about 20% wetland land cover, which is largely coastal wetlands.^76^ These data highlight the importance of lake, pond, and wetland ecosystems for these territories. Water and aquatic ecosystems are highly valued in Indigenous cultures for both the sustenance they provide and their spiritual significance.^443^, ^444^

##### 3.9.2.2 Impacts and risks

Climate change poses many challenges and risks to ecosystems within the territories that Indigenous Nations manage for agriculture, hunting, gathering, fishing, forestry, energy, recreation, and tourism. These threats to Indigenous lands must be understood in the context of historical injustices, from land dispossession to disproportionate impacts from industry.^445^ Indigenous Nations already face longstanding institutional barriers to their self‐determined management of water, land, and other natural resources. Because Indigenous health and well‐being is rooted in interconnected social and ecological systems, disruptions from a changing climate can “threaten sites, practices, and relationships that have cultural, spiritual, or ceremonial importance and that are foundational to Indigenous Peoples’ cultural heritages, identities, and physical and mental health.”^446^

There are widespread concerns that climate change impacts will exacerbate longstanding environmental justice issues and create new ones for Indigenous communities. For example, the Onondaga Lake and St. Lawrence River watersheds already have suffered severe ecological impacts from the industry. The Onondaga and Mohawk Nations’ ability to engage in fishing, water recreation, and plant harvesting has been negatively affected by extensive environmental degradation in their homelands. For these Indigenous Nations and others dealing with environmental justice concerns, climate‐induced changes in precipitation and temperature have the potential to worsen drainage and water quality problems. Sea level rise will have particularly severe effects on the Indigenous Peoples of Long Island due to a limited reservation land base directly abutting the ocean.^447^

Climate changes that affect culturally significant species are of particular concern for Indigenous Peoples. Species of concern include:

- **Sugar maple**. Maple sugaring is an important activity for Indigenous communities. Sugaring maintains traditions and helps Indigenous Peoples achieve food sovereignty. The timing of maple sugaring is expected to be disrupted due to changes in the freeze‐thaw cycle that drives sap production.^154^ Researchers from the Northeast Climate Action Science Center have engaged members of Indigenous communities as part of their studies of climate impacts on sugar maples.^448^
- **Sweetgrass**. Used by the Indigenous Peoples of New York in basketry and as a ceremonial herb, sweetgrass has long been declining in abundance. Indigenous communities have undertaken research and restoration efforts,^449^, ^450^ but concerns remain that climate‐induced changes to wetlands and rapid growth of invasive species could negatively affect sweetgrass habitat.^451^
- **Black ash**. Wood from black ash trees is used for baskets, lacrosse sticks, and other traditional crafts. However, black ash stands across New York State are threatened by the invasive emerald ash borer, which is projected to spread in the future. Impacts of climate change on black ash are being monitored closely by Indigenous craftspeople and environmental professionals,^452^, ^453^ as well as by other researchers and scientists.^454^
- **Quahogs (hard‐shell clams)**. Quahog shells are used to make wampum beads and jewelry and have always been highly regarded culturally. Indigenous Peoples of Long Island have also traditionally relied on clams and other shellfish for food. As one of the longest‐living marine animals, the quahog has drawn attention for what it reveals about climate change.^455^ Its habitat range is sensitive to ocean temperatures, and research about current and projected changes in quahog distribution is ongoing.

In addition to the species listed above, Indigenous Nations in New York State are concerned about a variety of fish species that they commonly harvest for food. For example, warming water temperatures in the rivers and creeks of Seneca Nation threaten populations of trout and walleye.^456^ Some Nations have invested extensively in fisheries restoration. Climate impacts on HABs and invasive species, as well as temperature and precipitation changes that affect fish habitat and distribution, are priority concerns for Indigenous fisheries managers and citizens.

#### 3.9.3 Native flora and fauna

BOX 13 **Takeaways**

- Plant and animal species will not respond uniformly to climate change. Beyond those occupying threatened habitat types, the species most vulnerable to extirpation from New York State include those on the southern edge of their range, those sensitive to warming temperatures or severe storms, those with low dispersal ability, and those strongly dependent upon other species under threat.
- Habitat loss and fragmentation remains the single biggest threat to the state's native flora and fauna, with climate change expected to exacerbate the impacts of that ongoing stressor.
- Some species may expand their ranges in New York or move into the state from neighboring states as New York's climate becomes more suitable for them. In some cases, these expansions will have impacts on other native species.

##### 3.9.3.1 Description and importance

Stretching from the Atlantic Ocean to the Great Lakes, ranging from sea level to elevations over 5000 feet, and feeding five major waterways that discharge to the ocean, New York State has a wide array of habitat types that are home to many thousands of species of animals and plants. It is no overstatement to say that this extraordinary biodiversity is the basis on which all life in the state depends, and the foundation for a wide variety of human interests, including wildlife photography, birding, botanizing, fishing, and hunting. More than 600 species of plants^457^ and nearly 500 species of animals^458^ are at some risk of extirpation from New York due to habitat loss and fragmentation, pollution, overharvesting, invasive species, and other factors.

##### 3.9.3.2 Impacts and risks

Native plants and animals vary widely in their responses to climate change. With warming temperatures, changing precipitation regimes, and increases in the frequency and intensity of extreme storm events, some species will increase in population and expand their ranges in New York State. Other species will decline as climatic conditions shift outside their range of physiological tolerances. Apart from these direct effects, a changing climate will also affect species indirectly by altering their habitats or causing shifts in the distribution and abundance of co‐occurring species. Such indirect effects may be subtle and take longer to detect, and are sometimes difficult to disentangle from direct effects. The sections above on specific ecosystem types provide examples of plant and animal species expected to be affected by climate‐induced habitat changes.

In general, distributions for many species are expected to narrow as compression at the warm edge of the range outpaces expansion at the cool edge.^106^, ^113^, ^459^ However, predicting biogeographical responses to climate velocities is challenged by several factors. These include variation in climatic tolerance among species, variability in habitat connectivity (such as in mountainous and marine habitats), and the availability of the nearest climatically suitable protected habitat.^101^, ^110^, ^111^, ^460^ Limitations imposed by organismal dispersal modes and landscape connectivity are expected to dictate whether a species can relocate quickly enough to keep pace with climate warming.^461^

A complete assessment of all of New York State's flora and fauna is beyond the scope of this document. An earlier assessment^302^ found that 70 of 119 at‐risk animal species assessed (59%) were moderately to extremely vulnerable to climate change. Later work incorporated landscape factors and species’ traits into finer‐grained products to make spatially explicit, species‐specific management recommendations.^462^, ^463^, ^464^ Following from those assessments and tools and the 2011 ClimAID assessment, this section describes critical vulnerabilities of several particular groups of species: those for which the state is at the southern edge of their range, those that are temperature sensitive and of low mobility, those that have close interdependency with other species, those that are commonly harvested by humans, and those that are already rare. This section also discusses plant and animal species that are poised to expand their range or increase their population in New York in a warming climate. Species highlighted as examples are primarily ones for which there is recent research specific to New York State or the northeastern states in general.

###### Species at the southern edge of their range

Species that have New York State as the southern edge of their range may be lost from the state if the climate becomes unsuitable for them. Most cold‐adapted species are alpine or boreal species that occupy only small areas of the state (mountaintops and boreal forest remnants, respectively, in the Adirondacks and Catskills). Mountaintop species in danger of disappearing from the state include Bicknell's thrush (Figure 5‐14), a specialist of high‐elevation spruce and fir forests—habitats that are expected to decline under most climate change scenarios.^465^ This thrush is further threatened by changing climatic conditions at its wintering grounds in the Caribbean,^466^ highlighting that climate change outside New York could affect members of the state's fauna that are migratory. Other montane birds such as the blackpoll warbler have shown uphill shifts in their distributions in the Adirondacks,^467^ complementing the northward shifts documented in New York's second Breeding Bird Atlas.^468^

**FIGURE 5‐14:**
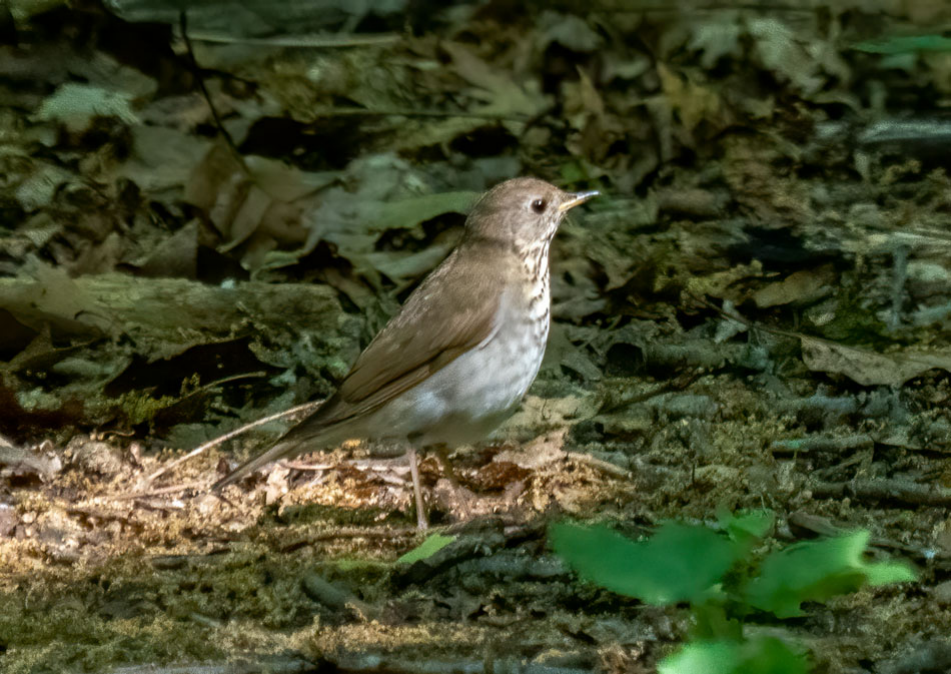
Bicknell's thrush. Photo by Ryan McGrady, licensed under CC BY‐SA 4.0.

The Adirondack population of the common loon, an iconic and cherished resident of Adirondack lakes, has recovered notably since the 1980s after being depleted by mercury pollution, acid rain, shoreline development, human recreation, and other threats.^469^ However, as with other species at the southern edge of their range, loons may face new stresses as a result of changing climatic conditions. In particular, warming temperatures and fluctuating lake levels (caused by changing precipitation patterns) could have direct impacts on loon nesting success.^470^

Animals of the “lowland boreal”—the assemblage of low‐elevation bogs, forested swamps, and other peatlands for which the Adirondacks are famous—face an uncertain future as well. Several species of boreal songbirds, including Canada jay, rusty blackbird, and boreal chickadee, are in decline due to changes in average temperature and precipitation.^471^ Another lowland boreal specialist, the spruce grouse, has declined in population and distribution in the Adirondacks, apparently as a result of changing habitat and genetic isolation rather than climate.^472^, ^473^ However, its presumed extreme sensitivity to climate change^302^ may yield future management challenges. Monitoring data for lowland boreal insects are scarce, but species occurring nowhere else in the state include various species of emerald dragonfly that are of conservation concern regionally.^474^

The moose is an archetypal mammal of northern forests that is at the southern edge of its range in New York. Suitable habitat for moose is predicted to decline in the Adirondacks and other northeastern regions under most climate scenarios, but protection of thermal refugia could allow populations to adapt to increasing temperatures.^475^

###### Temperature‐sensitive and low‐mobility species

Coldwater fish such as brook trout are a textbook example of a species sensitive to warming waters.^476^ Reptiles and amphibians, as ectotherms, are especially vulnerable to changing temperatures and are also constrained in their movement abilities. The eastern hellbender salamander (Figure 5‐15), which grows to lengths of up to 29 inches,^476^ is at once one of the most fascinating and most endangered amphibians in New York State. It occurred historically in the Allegheny and Susquehanna rivers,^477^ with limited occurrence in the Upper Susquehanna watershed.^478^ Laboratory studies have shown decreased growth rates for hellbenders subjected to warmer waters.^479^ As bottom crawlers that rarely swim, hellbenders could face considerable challenges in northward migration.

**FIGURE 5‐15:**
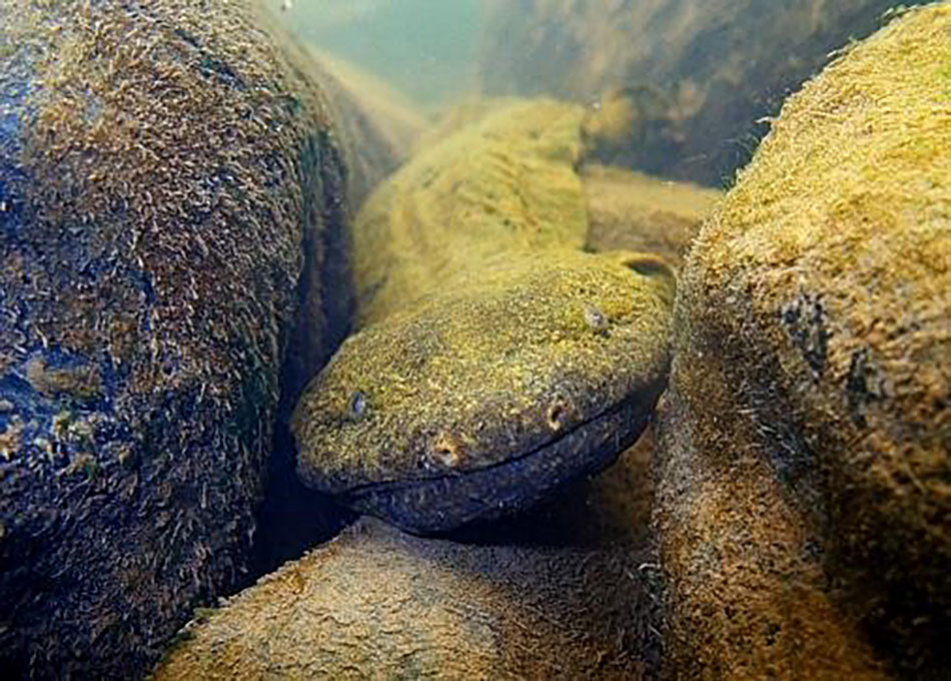
Eastern hellbender in the upper Susquehanna River watershed. Photo by Michelle Herman.

Freshwater mussels are one of the most threatened groups of animals, facing a variety of stressors including pollution and alteration of their habitats.^480^ Climate change, particularly extreme and erosive storm events and warming water temperatures, can exacerbate these threats and add new stressors. For example, some species of mussels have upper‐temperature limits beyond which they may be subject to thermal stress.^481^ Recent conservation status assessments for mussels in the state showed declines in most species over the last few decades.^482^ In the past year, the U.S. Fish and Wildlife Service has listed or proposed to list four additional mussel species currently or historically found in New York State.^483^, ^484^

###### Species with close interdependencies

In addition to their temperature sensitivity, mussels depend on host fish for dispersal of their larvae, and different mussel species rely on different fish species as hosts. Because of this, any change in the distribution of a host species can affect where mussels are able to disperse, particularly for mussels that are specialized for particular host fish species.

Insects often have tight mutualistic relationships with host plants, which serve as egg‐laying sites, sources of larval food, and sources of nectar and pollen for adults of some insect species. Wild bee populations have declined in the northeastern states,^485^, ^486^, ^487^ and an analysis of historical data showed that the timing of their emergence in spring has advanced by slightly more than 10 days on average, keeping pace with similar shifts in host plants.^488^ Few studies have investigated how climate‐induced changes in host plant distribution or physiology affect specialist pollinators, partly because of the long‐term, detailed data sets needed. However, mismatches in the timing of insect emergence and availability of floral resources remain a concern,^489^, ^490^ and changes in insect populations could have cascading impacts on species throughout the food web, including on birds that eat insects.^489^, ^491^

###### Commonly harvested species

The 2011 ClimAID assessment discussed the potential impacts of climate change on commonly hunted and fished species, and the main conclusions of that report remain valid. Decreased snowpack may lead to increased overwinter survival of white‐tailed deer, causing populations to grow in mountain regions where these deer are currently less common.^146^ This may require shifts in management in those areas to avoid damage to forest understory observed where deer are overabundant.^492^ Black bears, given their wide habitat and climatic tolerances, are expected to be adaptable to climate change in most of their range.^493^ Refer to Sections 3.5 and 3.7 for discussions of the predicted effects of warming waters on coldwater fish species and marine shellfish, respectively. Finally, the combined effects of climate change and harvesting have been forecast to increase extinction risk in the medicinal plant American ginseng,^494^, ^495^ and other commonly harvested medicinal plants may be at risk.^496^

###### Rare plants and animals

For rare plants, disentangling direct (physiological) and indirect (habitat‐based) effects of climate change is especially challenging because the plants themselves are often a key component of “habitat” for other biota. As with animals, the hundreds of rare plants in New York State will respond in varied ways to climate change. Rare plants expected to see impacts include species in presumed climate refugia on mountaintops and in deep gorges, species subjected to sea level rise, species in wetlands in a warmer upland matrix, and species that depend on ice scour for reducing competition with invasives and more common natives.^291^

New York's small alpine zone hosts many rare plants that occur nowhere else in the state, including purple crowberry, tundra dwarf birch, and Fernald's blue grass.^218^, ^220^ Effects of climate change on this natural community are discussed in Section 3.4. The federally listed northern monkshood occurs on Catskill summits, where increased temperatures may stress the plant,^497^ and in streamside habitat, where severe floods may be a threat.^498^ Other rare plants at the southern edge of their range, such as the purple mountain saxifrage (Figure 5‐16) and insectivorous butterwort, are usually found at higher elevations and latitudes but occur in New York in presumed glacial refugia in gorges and on cliffs where the cool, exposed habitat mimics those conditions. The current rapid rate of warming may exceed the physiological tolerances of these species, and their potential to migrate to new sites via seed dispersal is low.

**FIGURE 5‐16:**
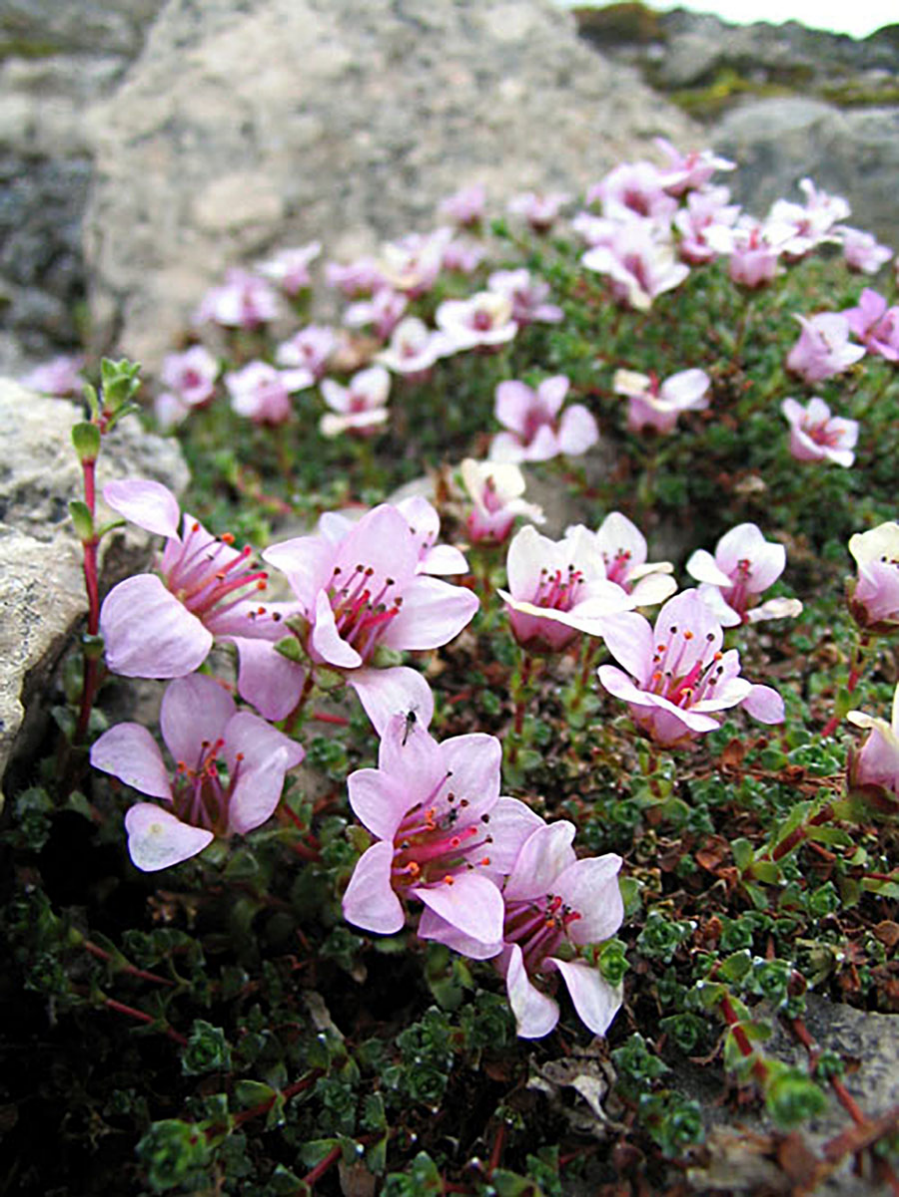
Purple mountain saxifrage. Photo by Michael Haferkamp, licensed under CC BY‐SA 3.0.

Beach specialists that are subject to inundation from sea level rise are addressed in Section 3.8. By eroding and shrinking beaches, rising sea level and major storm events threaten populations of obligate flora and fauna, which are already stressed by beach recreation and sand management. Species at risk include plants such as the federally listed seabeach amaranth,^499^ birds such as the federally listed piping plover,^500^ and insects such as the hairy‐necked tiger beetle.^501^ Riverside ice meadows support a unique assemblage of rare plants, including dwarf cherry and New England violet (which in New York State occurs only at a single ice meadow).^317^ These plants owe their persistence to annual ice scour that reduces competition and establishment by invasive species. Should ice cover decrease due to warming temperatures, the integrity of this community could be threatened.

###### Species preferring warmer conditions

Climate change is expected to facilitate the range expansion of some plants and animals, both within New York State and into New York from neighboring states. Species with strong dispersal capabilities will be favored in this regard. A general finding among migratory birds is that the “latitudinal distributions of temperate‐wintering species have increased while the latitudinal distributions of neotropical migrants have decreased.”^502^ Researchers expect farther spread northward and upslope of birds that have started breeding in New York in recent decades, such as northern cardinal,^468^, ^503^ red‐bellied woodpecker,^468^, ^504^ and black vulture.^505^, ^506^ Birds that may begin breeding in New York in future decades, based on their current breeding range south of the state and documented recent northward shifts, include summer tanager and Carolina chickadee.^507^, ^508^ The Carolina chickadee is known to hybridize with the black‐capped chickadee,^508^ which could have unknown consequences for that abundant New York native.

Future climatic conditions in New York State may be suitable for some reptiles and amphibians whose current ranges end just south of the state, such as the carpenter frog, ground skink, and northern pine snake. However, other factors, including interspecific competition and dispersal barriers such as roads and large waterways, may prevent these species from moving into the state. Warming conditions may favor range expansion by New York's three current species of lizards, but habitat and dispersal limitations may prevent this. It remains to be seen whether the 2018 nesting on Rockaway Peninsula by a Kemp's ridley sea turtle—the first‐ever sea turtle nesting recorded in New York State^509^—was a fluke occurrence or a harbinger of things to come.

Flying insects will continue to colonize New York and expand their ranges within its borders. The giant swallowtail butterfly has shifted its range northward at a pace consistent with warming temperatures. It could continue its spread where suitable host plants are available.^510^ A recent statewide survey of dragonflies and damselflies documented several “southern” species not previously documented in New York,^511^ and select species have been documented expanding their ranges northward as temperatures rise.^512^, ^513^

Such changes in species distribution will not always be welcome. For example, the destructive southern pine beetle will continue its spread north with increasingly mild winters.^514^ Some species’ movements could displace other native species, as in the case of southern flying squirrels displacing northern flying squirrels in nearby states.^515^, ^516^ Moreover, the spread of certain species could lead to more frequent conflicts with humans, as has been documented with expanding black vulture populations.^517^

###### Other impacts

A variety of less easily categorized impacts from climate change on animal biodiversity are being observed in New York State and the region; the impacts are as varied as the species themselves. For instance, the reduction in snow cover has led to a “camouflage mismatch” for snowshoe hare, whose seasonal coat change from white to brown is now happening well after snowmelt in many years. This has resulted in increased predation risk for hares^518^ and caused their range to contract northward.^519^, ^520^ Mammalian predators who undergo similar color changes in winter, like the ermine, may also be at risk. Climate change has facilitated the spread of some diseases, such as the ranaviruses that affect frogs (and other ectotherms) in the northeastern United States and elsewhere.^521^, ^522^ There are numerous cases where changing climatic conditions have accelerated the spread of invasive species, affecting native organisms (refer to Section 3.9.4).

#### 3.9.4 Invasive species

BOX 14 **Takeaways**

- Human transport remains the primary driver of species invasions, and prevention efforts designed to address climate change are best served by focus on invasive animals, plants, and pathogens arriving from warmer, wetter locations.
- A drop in the number of freezing days will allow new pests, such as tree‐feeding insects, to survive winter, and warmer average air temperatures will permit established pest populations to grow faster and larger.
- Future climate‐related impacts will reach all regions and all sectors identified in this report, but New York has a well‐developed, statewide network of professionals and volunteers engaged in invasive species management.

##### 3.9.4.1 Description and importance

The definition of *invasive species* applied by the New York State Legislature originates from Federal Executive Order 13112 (1999).^2^ It stipulates that invasive species are non‐native to the ecosystems of concern and cause economic or environmental harm or threaten human health. This section focuses on damage caused to New York State ecosystems by invasive plants, animals, or microbes. When discussing invasive species, it is helpful to distinguish exotic or alien species transported to a region by human activities from neonative invasive species that have migrated into a region through natural dispersal.^523^ Natural dispersal of a neonative species may be facilitated by human activities such as land‐use change and climate change without a direct human vector. Per a 2016 amendment to Executive Order 13112,^524^ federal agencies charged with the prevention, control, and eradication of invasive species must also consider the impacts of climate change on species invasions. A recent survey of state experts who research and manage invasive species found that climate change was one of their most pressing challenges.^525^ In response, a regional network and data‐driven method for prioritizing management has been established to provide guidance to land managers and policymakers on how to address this growing challenge.^526^, ^527^

Biological invasions proceed in steps—from transport at points of origin, to alerts of new arrivals, to evidence of threats and damage. However, most species invasions are not detected until damage is underway.^528^, ^529^ A primary driver of species invasions has been transit by human commerce,^528^, ^530^, ^531^ and new global climate patterns are affecting commercial transit routes (e.g., new shipping ports) and sources (e.g., shifting agricultural zones) of goods, as well as the potential for newly arriving species to become established.^530^, ^532^, ^533^ Another driver is natural or human‐induced ecosystem disturbance, which creates opportunities for non‐native species to invade new ecosystems^534^ and is increasingly linked to climate change—particularly extreme events. Vegetation management using motorized vehicles along linear features such as power line corridors was shown to be a common vector for the dispersal of invasive species.^535^

Calculating the full economic, social, and environmental costs of invasive species has proven difficult, but even cautious analyses have found that preventing and managing their spread is highly cost‐effective.^536^ The most recent estimates of financial damage caused annually by invasive species in the United States put the cost at almost $120 billion,^537^ but researchers emphasize that the problem is still growing. For example, pathways for introduction and spread are expanding via shipments from internet commerce.^538^, ^539^ A recent estimate shows that invasive species have caused almost $500 billion in financial damages in the United States since 1980, making them the second‐most destructive category of natural disaster—more destructive than floods, droughts, and wildfires.^540^

The northeastern United States, including New York State, has been identified as one of three global hot spots for invasive species.^541^ Recent and emerging threats to the state's ecosystems include beech leaf disease (Figure 5‐17),^542^ emerald ash borer,^543^ round goby,^544^, ^545^ spotted lanternfly,^546^ hemlock woolly adelgid,^547^ and exotic earthworms.^548^ Tree of heaven is an invasive tree species that is likely to invade New York forests in the coming decades.^549^ Meanwhile, numerous well‐established invasive species such as *Lymantria* moths continue to attack forest ecosystems.^550^ In some cases, these and other pests benefit directly from climate change (e.g., refer to the Hemlock Pest case study). In other cases, the damage from invasive species is compounded by climate hazards that weaken native organisms or provide an advantage that allows invasives to outcompete their native counterparts. The spread of some invasive species in New York State, such as the emerald ash borer, does not appear strongly linked to climate change at present,^551^ but future climate change could allow these species to spread to regions that are not currently suitable habitat.^552^

**FIGURE 5‐17:**
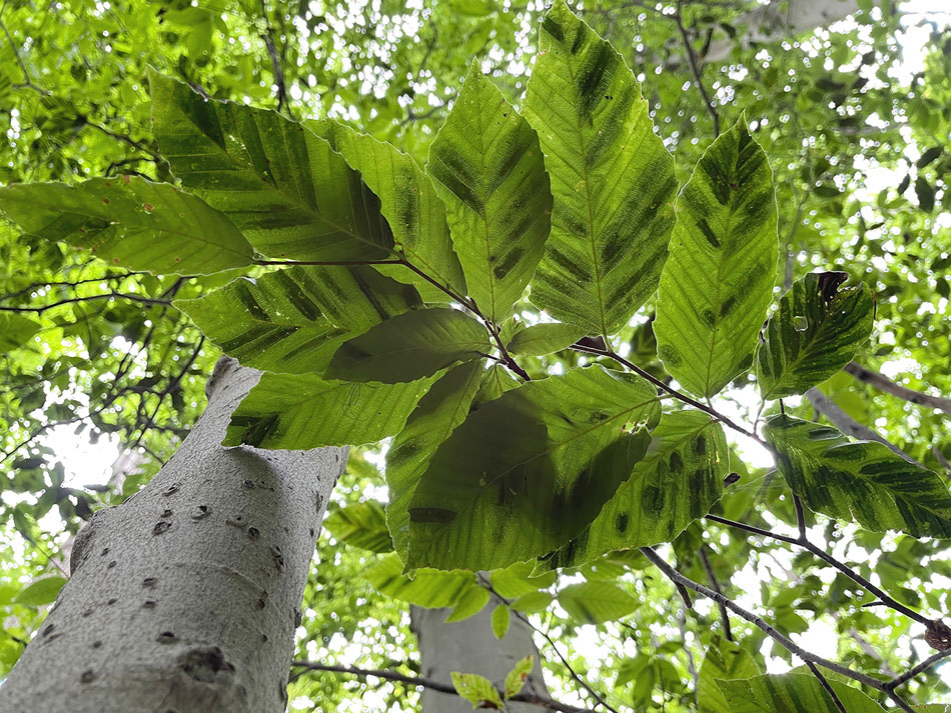
Beech leaf disease. Photo by Matt Borden, licensed under CC BY‐SA 2.0.

Since the publication of the 2011 ClimAID assessment, laws, regulations, and networks of organizations in New York State have evolved to address threats from invasive species. Title 17 of the Environmental Conservation Law (revised 2014) established an Invasive Species Council (section 9‐1705) and an Invasive Species Advisory Committee (section 9‐1707), both of which include public and private partners.^553^ These organizations communicate with similar organizations in other states and provinces, including through the regional network mentioned above, and with federal agencies. The New York State Department of Environmental Conservation (NYSDEC) has added a Bureau of Invasive Species and Ecosystem Health to support Title 17. The bureau funds eight Partnerships in Regional Invasive Species Management (PRISMs),^554^ which are geographically distributed. PRISMs comprise interested parties from the public and private sectors. Their shared mission includes prevention, detection, and control of invasive species, as well as public outreach and education. PRISMs have access to a database of invasive species locations and tools for species identification and communicating management strategies (iMap Invasives). PRISMs were used to develop a prioritization scheme for managing non‐native invasive plants statewide and regionally.^527^ The New York Invasive Species Research Institute helps coordinate and communicate invasive species research and improve the scientific basis of decision‐making related to invasive species. Title 17 also invests state agencies with regulatory authority, which to date has been used for issuing lists of prohibited species and rules for cleaning watercraft. New York's comprehensive system for addressing the threat of invasives is unique to the state and serves as a model that other states are seeking to follow.

##### 3.9.4.2 Impacts and risks

Experts anticipate that changing climatic conditions will affect the spread of invasive species in two major ways. First, a drop in the number of freezing days will allow overwintering pests, many of which originated in warmer climate zones, to survive. Second, warmer average air temperatures and longer growing and reproductive seasons will permit established pest populations to grow faster and reach a larger total size. Another possible impact is a direct result of increases in atmospheric carbon dioxide concentrations. Experiments and observational studies indicate that invasive plants are able to take better advantage of elevated carbon dioxide levels and could outgrow native species and crop plants.^555^, ^556^ Other studies suggest that chemical controls become less effective against weeds as carbon dioxide levels rise.^557^ Figure 5‐18 summarizes several of these connections between invasive species and climate change.

**FIGURE 5‐18:**
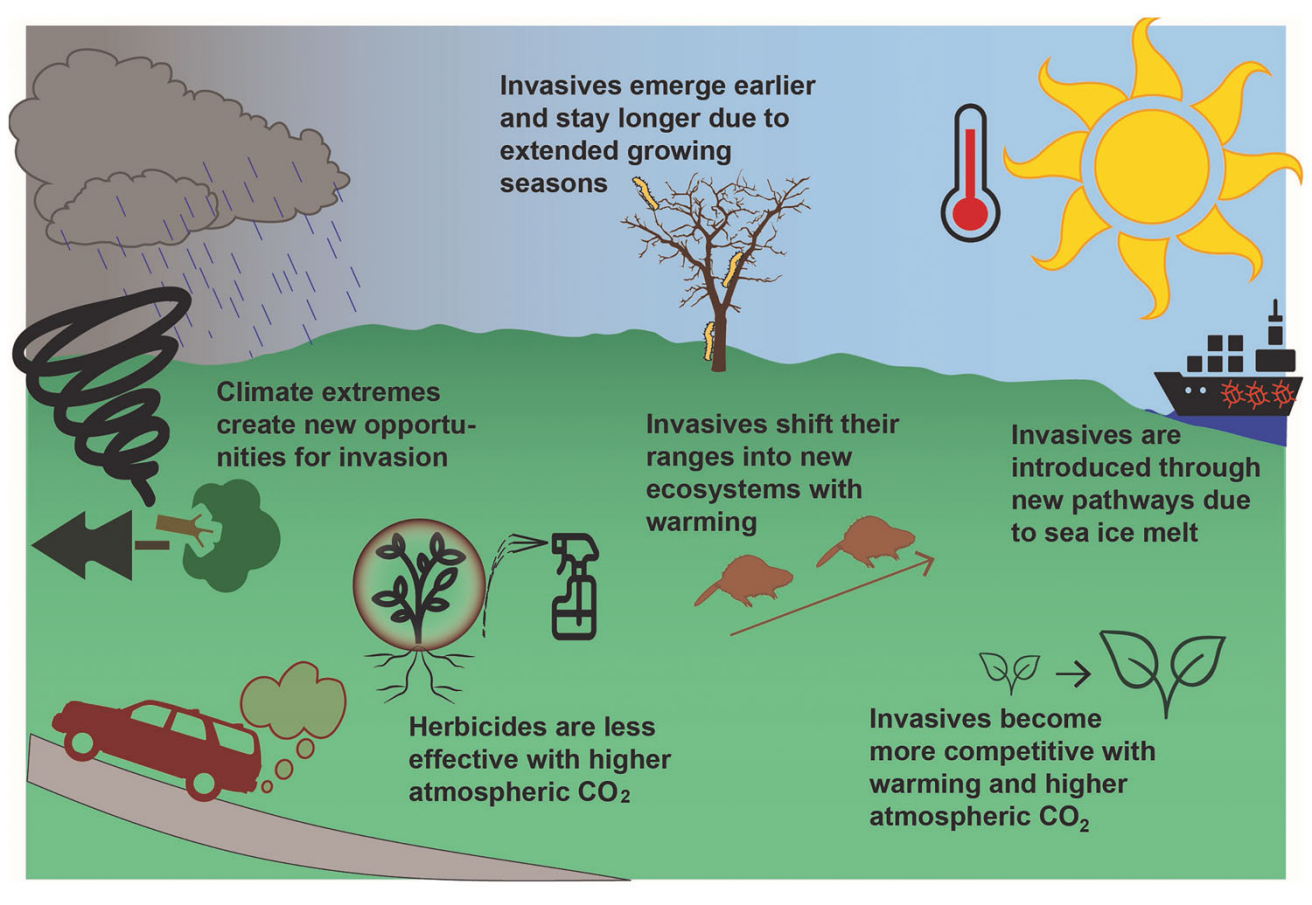
Major interactions between invasive species and climate change. Figure from Bradley et al.^558^

Based on these climate‐related impacts, scientists and resource managers anticipate new waves of invasive species and more damage to warmer terrestrial and freshwater ecosystems. They also see the possibility that warming coastal waters will create new opportunities for aquatic invasive species in the Northeast, including in New York.^559^ Other potential risks include:

- Vector‐borne pathogens—namely tick‐ and mosquito‐borne diseases—could continue spreading north and west.^560^, ^561^
- Invasive plants could continue spreading by horticulture exchange, with new exotic species arriving as growing zones shift.^530^, ^538^
- Urban heat islands coupled with habitat disturbance already favor some invasive species,^562^ and projected warming will intensify this situation.^563^
- The spread of aquatic weeds via watercraft could increase as boating seasons grow longer. Aquatic invasive plant species already disrupt many of the state's freshwater ecosystems.^564^
- Stresses to native flora and fauna from heat, drought, and a changing hydrological cycle could reduce native species’ ability to compete with invasive species, rendering them more vulnerable to aggressive invaders.^565^

##### 3.9.4.3 Regional variation

Regional differences in current and projected climate variables are better understood in the context of geographic differences in susceptibility to invasive species. For example, much of Long Island has a thriving trade in horticultural plants, due in part to its more temperate coastal climate. The steady flow of exotic plants into the region raises the risk that invasive species will be introduced as well.^186^, ^566^ Aquatic ecosystems from the Hudson River estuary to the New York Bight carry large volumes of commercial and recreational boat traffic that can spread invasives from abroad and between local areas. A recognition of regional variability in invasive species distributions and management needs underlies the organizational model of the PRISM network, and each of the eight partnerships has its own unique sets of plans and priorities.^554^

### 3.10 Cascading and cross‐sector impacts

While individual climate hazards can have major impacts, the interactions of climate effects and their compounding and cumulative impacts on ecosystem processes pose a greater risk in terms of magnitude and uncertainty.^80^ For example, warming temperatures combined with seasonal changes in precipitation result in asynchronous phenological shifts among interdependent species (e.g., flowering plants and pollinators), which could disrupt the resilience of species populations, ecosystems, and ecosystem services.^7^ Other cascading impacts are associated with temporal and spatial collision of multiple impacts and vulnerabilities. An example could be fragmentation, loss, and degradation of forest ecosystems resulting from a localized “perfect storm” of climate impacts such as disturbance from an extreme storm in a forest already in decline due to invasive species and with impaired regeneration due to deer browse and herbivory.

The combination of climate and nonclimate stressors can also be a major driver of change, sometimes resulting in profound and unpredictable consequences for ecosystems. These combined impacts can lead to cascades of ecological changes that reverberate in multiple sectors. One example of cascading impacts that cross multiple sectors is the climate‐induced northward spread of the invasive hemlock woolly adelgid. Decimation of hemlock stands in ravines along headwater streams can result in a loss of dense canopy along the riparian corridor and destabilization of steep slopes. These changes can lead to increased penetration of sunlight, warming waters, and a greater risk of erosion. Vulnerability to erosion is further compounded by an increase in the frequency and intensity of extreme storm events. Hemlock loss also reduces available habitat for a wide range of insect and bird species.^567^ These compounding factors and cascading events eventually lead to reduced water quality in the upper reaches of the watershed, negative impacts on aquatic biota (including eastern brook trout), and increased vulnerability of both the stream and the riparian corridor to invasive plant species infestation. This further translates to multisector impacts on society and economy (recreational fishing and ecotourism), human health, and water resources (water quality). The Hemlock Pest case study describes this cascade of impacts in greater detail.

Ecosystems are the foundation of human resilience, serving as the primary source of life‐sustaining materials and processes. Impacts to ecosystems and ecosystem services have a corresponding effect on human communities. Thus, climate impacts to ecosystems inevitably affect other sectors. Additionally, human actions within other sectors can contribute to the conservation and resilience of ecosystems and ecosystem services—or to their degradation and loss. Because of these connections, there are important opportunities for coordinated efforts to design and implement ecosystem management and climate adaptation strategies that benefit multiple sectors within New York State's communities.^12^ For example, integration of nature‐based climate solutions into economic development projects and local government land‐use policies that support the protection, restoration, and reconnection of ecosystems will increase climate resilience by supporting ecosystem services and reducing negative impacts to air and water quality, real estate value, environmental justice and equity, and public health. Additional compounding factors and cascading impacts associated with ecosystems and other sectors are briefly explored below. The discussions of urban ecosystems in Sections 3.9.1 and 4.8 provide additional examples of cross‐sector considerations.

#### 3.10.1 Agriculture

Agriculture and riverine ecosystems are closely connected and subject to compounded climate impacts such as catastrophic floods that simultaneously damage farmlands and pollute streams and rivers. Collaborative floodplain management can ameliorate both impacts. Establishment and preservation of riparian forest buffers and other natural areas adjacent to or within agricultural landscapes is an effective and inexpensive climate adaptation that can protect farms from floods, crop loss, and soil erosion while also supporting native pollinators that bolster crop pollination.^568^, ^569^ A lack of natural buffers increases the impact of climate hazards on farmland and also increases the deleterious impacts of agricultural activities. Refer to the Agriculture chapter for additional discussion.

#### 3.10.2 Water resources

Ecosystems such as headwater streams, wetlands, and upland buffers provide the critical service of protecting water quality. Climate hazards such as rising temperatures and extreme storm events lead to direct and indirect impairments of this vital function due to increased water temperatures; flooding; erosion; and an influx in sediment, toxins, and pollutants. Loss of water quality affects the water resources that human communities depend on and raises the cost of water treatment. Adaptive management strategies, including reconnecting floodplains and protecting wetlands, headwater streams, and riparian buffers, are the most cost‐effective way to support water resources in the face of climate change.^570^ Refer to the Water Resources chapter for additional discussion.

#### 3.10.3 Human health and safety

Climate change impacts on ecosystems can present risks to human health and safety. For example:

- Climate change impacts on species distributions, combined with the fragmentation of natural areas and connecting corridors, can increase the risk for negative human−wildlife interactions, including the spread of disease associated with mosquitoes and ticks.^571^
- Declines in fisheries and terrestrial game species can result in a negative impact on subsistence harvest activities and consumptive recreation.
- As noted in Section 3.5, higher nutrient loads and increased water temperatures can result in the degradation of aquatic ecosystems, leading to a decrease in water quality. Under such conditions, there could be an increase in the frequency, duration, and extent of HABs and the spread of infectious diseases (for more information, refer to the Harmful Algal Blooms case study).
- Contamination in aquatic environments increases substantially after intense storm events and includes pollution that results in negative health issues and even mortality, although deaths from contaminated aquatic environments are rare in developed nations.^572^
- Loss of natural environments or reduced access to natural areas due to extreme events (heat waves, intense storms) can exacerbate mental and physical health issues by removing the health benefits that exposure to natural areas provides.^7^

A specific example of how compounding and cascading impacts can lead to human health concerns is associated with sea level rise and coastal communities. Sea level rise, coupled with an increase in the frequency and intensity of storms, will cause storm surges to reach farther inland with greater volumes of floodwater, resulting in greater impacts on coastal and inland ecosystems. Water in aquatic ecosystems—already contaminated by sediments; nutrients; toxins; and pathogens from agriculture, stormwater, and sewage—will further decline in quality as inland contamination is mobilized by inundation. The increase in contaminants, combined with higher temperatures, will cause fish and other organisms to accumulate toxins at higher rates. These events could create public health concerns associated with water quality and consumption of fish, particularly in environmental justice communities. The cascading events associated with storm surge are discussed in more detail in Section 3.8. Refer to the Human Health and Safety chapter for additional discussion.

#### 3.10.4 Society and economy

Without exception, every aspect of society and the economy can be traced to the provisioning, regulating, supporting, and cultural services of ecosystems. These services provide fresh water, wood products, floodwater retention, air and water purification, soil formation, biodiversity, recreation, and education. Climate impacts on ecosystems result in countless examples of cascading impacts that affect society and the economy. For example, warming water temperatures can lead to species and regime shifts (e.g., predator/prey dynamics), increased thermal stress and disease susceptibility, and changes in the timing of spawning activities. These changes, in turn, can affect commercial fisheries and fishery‐related recreation and subsistence activities, which can negatively affect associated communities and economies. Climate change impacts on ecosystems and species of cultural significance can affect jobs, recreational opportunities, spiritual traditions, and nature‐based tourism. Rural communities depend on ecosystems to support jobs based on agriculture, silviculture, and ecotourism. Climate impacts on ecosystems can affect entire industries. The logging and forest products industry, in particular, will require significant adaptation. Profitability is already marginal for many logging businesses,^573^ and effective climate adaptation will require substantial equipment upgrades and capital investment.^197^ Cut‐to‐length harvesting systems can help address the challenges of operating on wet soils, but these are among the most expensive production systems in the industry and cost more than whole‐tree harvesting.^195^, ^197^ In forested areas, many roads and landings that were suitable for winter use will require extensive upgrades if they are to be useful for logging activities through wet summers and warm winters. The operational adaptations required to maintain production are reasonably well understood, but the economics of those adaptation measures are less clear.^197^ Refer to the Society and Economy chapter for additional discussion.

#### 3.10.5 Buildings, transportation, and energy

Degradation and loss of ecosystems such as wetlands and riparian floodplains due to climate impacts can result in increased flood peaks and flood duration, which in turn can damage buildings, roads, bridges, railways, and energy infrastructure. Loss of tree cover in urban and suburban areas can increase energy demand. Conversely, protecting and restoring ecosystems can be a relatively inexpensive way to protect infrastructure from climatic events. Poorly planned development, renewable energy, and transportation projects result in the continued fragmentation of ecosystems and an increase in impervious surfaces, leading to the loss of the same ecosystem services that could have protected the newly built infrastructure. Numerous climate adaptations and best management practices can help build resilience for both ecosystems and the built environment. Refer to the Buildings, Transportation, and Energy chapters for additional discussion.

## 4 KEY VULNERABILITIES AND ADAPTATIONS

Based on the assessment of climate impacts detailed in Section 3, key ecosystem vulnerabilities emerge. Highly vulnerable ecosystems and species are often those that are most constrained by inherent traits and/or affected by factors in addition to climate change. Adaptations to moderate the effects of climate change can target varying scales (e.g., species, habitats, ecosystems) and address attributes; processes; and services such as biodiversity, nutrient cycles, or floodwater retention.^574^ Adaptive measures can be responsive to observed climate change impacts, preparative for anticipated impacts of climate change, and proactive to reduce the extent of potential future change. Depending on geography, ecosystem type, and resource availability, ecosystem management can offer opportunities for each of these types of measures. For decades, ecosystem management has sought to adapt and respond to nonclimate stressors, including general loss of ecosystem and habitat area; disconnection between ecosystems, species movement corridors, and resources; overharvest; and chemical and biological stressors such as acidification from acid rain, HABs, and eutrophication. Ecosystem management that seeks to adapt to new stressors must now consider both climate and nonclimate stressors for successful management and climate‐resilient outcomes. However, adaptation strategies have often fallen short by being too incremental and by failing to evaluate outcomes, which heightens the challenge of meeting this moment when climate change is increasingly affecting New York State's ecosystems.^575^ The following sections outline important adaptation and resilience actions for major ecosystem types and cross‐cutting topics and provide a summary of resources to support ecosystem adaptation in the state.

### 4.1 Forests

Key vulnerabilities of forest ecosystems to climate change over the next 80−100 years include:

- Amplified impediments to forest regeneration and productivity due to summer drying, increased herbivory, and invasive species.
- Increased disturbance regimes from drought, intense wind events, freeze‐thaw cycles, extreme precipitation, flooding, and, potentially, wildfire.
- Increasing impacts to spring ephemeral plant and animal species using the forest understory.
- Sea level rise impacts on coastal forests and tidal wetlands.
- Tree loss due to forest pests, diseases, and invasive species (e.g., hemlock woolly adelgid).
- Shifts in hydroclimatic regimes in vulnerable forest communities, including subalpine and coastal pine barrens.

The forest climate adaptation literature is extensive.^126^, ^127^ NYSDEC provides specific information and adaptation priorities for New York State's forests on the NYSDEC website and in the two reports *New York State Forest Action Plan* ^119^ and *Landscape Planning Guide for Managing Forests in a Changing Climate*.^576^ The discussion below consolidates the key strategies that have been identified in the literature and are being developed and implemented by forest managers on the ground.

Activities that support existing forest ecosystems include protecting forests from conversion and fragmentation, using best management practices to avoid impacts to topsoil and environmentally sensitive areas within forests (streams, vernal pools, seeps), and managing invasive species. Restoration and enhancement of forests is a key strategy in climate adaptation. Management that promotes overall resilience diversity of the forest ecosystem, along with practices such as facilitated migration of resilient species and transitioning some stands to new species compositions, could help address increasing challenges with forest regeneration, climate stress, and slowing growth rates of resident species.^577^, ^578^, ^579^

Two categories of forest deserve special mention: old‐growth forest and forever‐wild forest. Old growth generally refers to forests in a late successional stage with many large trees that have not experienced past human or catastrophic natural disturbance, although definitions of old growth are highly variable, can include many biological and physical traits, and vary regionally.^580^ Old‐growth forests are valued for their capacity to store carbon, as well as many ecosystem services, such as biodiversity enhancement, protection of air and water quality, and scenic beauty.^581^ New York State is estimated to have 200,000 or more acres of old‐growth forest, and much of this forest is protected from human disturbance.^582^ To maintain old‐growth status, these forests can tolerate only minimal active human intervention, and management strategies across the globe have generally focused on preservation to allow the continuation of the natural climate change resilience they provide.^583^ Forever‐wild forest is defined in the New York State constitution and includes nearly 3 million acres in the Adirondack and Catskill Parks.^584^ This designation provides protection; thus, the role of forever‐wild forest land in climate change resilience and mitigation has largely focused on preservation to maintain the valued ecosystem services these forests provide (as with old‐growth forests). Many of the climate change adaptation strategies currently being discussed by the management community such as assisted species migration cannot be implemented in New York's forever‐wild forest land, limiting resilience strategies.^585^ At national and global scales, however, the state's forever‐wild forest contributes to resilience strategies focused on the preservation of biodiversity and maintaining habitat connection.^586^

Climate‐adaptive forest management strategies for managed timber stands include general silvicultural best management practices. A forest with structural and compositional diversity fosters climate resilience. Having diverse species mixtures and complex stand structures and arrangements creates a wide variety of microenvironmental conditions and maintains a broad suite of functional traits, including support for air and water quality, biodiversity, aesthetics, silviculture, and recreation. Such diversity also hedges against the risk of a single disturbance agent (e.g., insect pest) affecting most of a forest.^587^

Other stand‐level strategies to increase diversity and to promote desirable traits in individual trees include variable density thinning and nonuniform regeneration methods, such as irregular shelterwood methods or multitreatment methods.^588^, ^589^ At the landscape level, structural and compositional diversity are attained by varying the timing, intensity, and methods of regeneration treatments and thinning regimes. Importantly, landscape‐scale diversity sometimes requires silvicultural treatments that reduce diversity and complexity at the stand scale. Such treatments might be needed, for example, when young forests or early successional tree species are underrepresented on the landscape. In many areas of the state, the main regeneration challenge is not an overall lack of regeneration, but rather that undesirable tree species are regenerating, resulting in low biodiversity.^590^ However, 32% of the state may not have sufficient regeneration to replace the forest canopy should overstory disturbance occur.^591^ High deer pressure is a major factor contributing to forest fragmentation and a lack of regeneration, and climate plays an important role in regeneration while also interacting with deer pressure.^189^ While this suggests that managing deer could improve tree regeneration, climate and landscape connectivity also have an impact and are more difficult to manage. Together, these factors highlight the challenges of maintaining current and future forest resilience in the face of disturbances such as climate change.

The Menominee Nation in Wisconsin (Forest Keepers) provide an example of ecosystem stewardship and climate‐adaptive forestry by using traditional forest management practices while allowing foresters (and their observation and knowledge of the forest) to determine annual harvest, instead of the mills and the market.^592^ The Haudenosaunee Confederacy in New York State have long traditions of effective forest stewardship that include the use of fire as a management tool and assisted migration and cultivation of plants species, including several trees and medicinal forest herbs more common in the southern United States such as butternut, sassafras, wild ginger, and elderberry.^452^, ^593^

Prescribed fire can also be applied as a management strategy to address many of the climate change risks to forests discussed in this chapter and to maintain biodiversity and habitat for rare species and wildlife, as well as to manage invasive species such as the southern pine beetle.^119^ Furthermore, prescribed fire can reduce the risk of more destructive wildfires by reducing fuels.^119^ Prescribed fire is currently being used for management in fire‐dependent forests in the state in areas such as the Albany Pine Bush, the Shawangunk Ridge, and Central Long Island.^119^

The strategies discussed above are all likely to create value for landowners and society across any plausible climate scenario. New York State has extensive forest reserves as well as actively managed forests. All forests are important to climate resilience and for achieving the state's net zero emissions goal by 2050.^594^ Helping forests meet their resilience potential through a myriad of conservation strategies will be necessary to meet that goal.

### 4.2 Open lands

Land‐use change and natural succession will continue to affect open land terrestrial communities such as grasslands and successional old fields. Open lands are particularly attractive for renewable energy project development because of low clearing costs.^595^ Climate change could increase pressure on open lands if it leads to additional development for renewable energy projects, changes in agricultural practices, and hydrological change such as drought or waterlogged soils. The rate of transition of successional old fields into early successional forests could increase with a warming climate in some regions.^596^ Vulnerable species include threatened or endangered grassland birds that require relatively large tracts of grassland and are sensitive to changes in hydrology, as well as pollinator species that depend on specific plants and their associated phenology. Climate‐related vulnerability is also evident in the current roster of invasive plants spreading across New York State, which includes a preponderance of species that grow in full or partial sunlight.^597^

Most of New York's open lands are dynamic systems that rely on natural processes (e.g., wildfire), managed processes (e.g., controlled burning or mowing), or local environmental features (e.g., naturally poor soils) to remain in open, nonforested states.^598^ Species that inhabit them are vulnerable not only to shifting management (e.g., new agricultural practices) but also to habitat loss due to wholesale land use conversion.^599^ In cases where active management is required to keep protected open lands in a desirable state, adaptation could entail revising management strategies, including seasonal adjustments. Example strategies include prescribed burning and grazing.^599^, ^600^ In more extreme cases that override current management options, adaptation could require transplanting better‐adapted species (if available) or shifting management to more suitable locations.

### 4.3 Lakes and ponds

Climate change is altering the physical structure of lacustrine ecosystems in critical ways that affect the chemistry and biology of these ecosystems. For example, ongoing reductions in ice cover and consequent increases in water temperature are altering patterns of thermal stratification, often contributing to the deoxygenation of deep‐water habitats.^601^ These changes can increase phosphorus releases from sediments, which can stimulate high algal growth and lead to conditions favorable to cyanobacterial blooms.^602^ In turn, HABs can produce toxins that are harmful to people and wildlife. Lakes that are already severely impaired due to factors such as anthropogenic inputs of nutrients could be at the greatest risk of experiencing climate‐related increases in algal blooms, reductions in water clarity, and increases in cyanobacterial toxins. Continued monitoring of temperature, dissolved oxygen, and nutrient conditions is essential to understanding climate impacts on lacustrine ecosystems. Climate change has heightened the need for monitoring and management of nutrient sources to lakes.

Increases in temperature and decreases in dissolved oxygen regulate carbon‐processing rates, which affects the exchange of greenhouse gases between aquatic ecosystems and the atmosphere. However, the effect of climate change on the direction and magnitude of greenhouse gas exchange is not straightforward. For example, increases in anoxia lead to increases in aquatic sediment organic carbon sinks,^603^ as well as the production of potent greenhouse gases such as nitrous oxide and methane.^604^ Monitoring of aquatic greenhouse gas fluxes is critical to understanding the role of lakes in the carbon cycle and overall interactions with climate change.

Changes in oxythermal habitat alter the community composition of lake fisheries and the potential for species invasions. For example, the sea lamprey is a parasitic invasive fish species commonly found in the Great Lakes that is responsible for historic population declines among lake trout, lake whitefish, and burbot and is expected to benefit from rising water temperatures.^605^ Sea lamprey can also survive in low oxygen conditions due to its high concentrations of hemoglobin, so low dissolved oxygen concentrations from increasing water temperatures are less likely to increase mortality in this species than in native species.^606^ Continued warming is likely to expand the range of many other invasive fish species such as snakehead and reptiles such as red‐eared sliders into the Great Lakes in the coming decades.^607^, ^608^ Independent of species invasions, increased water temperatures will also lead to decreased growth rates in coldwater species living at the boundaries of their habitable ranges. Moreover, climate‐induced changes in prey availability will have as much of an impact on native fish populations as changes in abiotic habitat condition.^609^ For example, as the timing of spawning activity changes in reaction to warming water conditions, hatched fish larvae may have less access to zooplankton prey,^610^, ^611^, ^612^ further challenging fisheries and overall food web dynamics. New York State has implemented an aquatic invasive species management plan, which is designed to be modified frequently as climate change increases the challenge of limiting the impact of invasive species on lakes and ponds in the state.^564^

Biodiversity monitoring, such as through eDNA techniques, is proving to be a helpful tool for characterizing the extent of the impacts of climate change on aquatic ecosystems.^613^ Adaptation strategies focused on maintaining connectivity to coldwater refugia, enhancing aquatic shading where practical, and promoting management practices such as dam releases of cold bottom water will benefit thermally stressed species in the coming decades.

### 4.4 Wetlands

Worldwide, the continued loss and degradation of wetland ecosystems results primarily from nonclimate stressors associated with residential/commercial development and agricultural activities.^614^ Projected increases in precipitation, temperature, and periods of drought due to climate change will intersect with these nonclimate stressors, with a disproportionate impact on small, isolated wetlands; coastal wetlands; riverine wetlands in developed watersheds; managed wetland impoundments; and newly restored wetlands that fail to consider changing climate variables.^615^ Increases in the frequency and severity of extreme storm events will have substantial negative impacts on vulnerable wetlands, and these impacts are difficult to predict in terms of both timing and location.^300^ Given the multiple stressors affecting wetlands, including projected climate change impacts, wetland ecological services will likely decline as the number and extent of wetlands shrink and their functionality is impaired. Concurrently, as the climate changes, ecosystem services provided by wetlands will become increasingly valuable due to the capacity of wetlands to store carbon, filter and store water, buffer storm surge, and harbor diverse species populations.^616^ Adaptations that improve the protection, management, and restoration of wetlands in response to climate change impacts are, in themselves, important nature‐based climate solutions.

Protection of existing wetlands is by far the most cost‐effective method for maintaining the ecological services that wetlands provide. Conservation strategies that focus on the protection and restoration of coastal wetlands, large wetlands, wetland complexes, and small wetlands such as vernal pools that support unique and vulnerable wildlife species will contribute to the resilience of wetland ecosystems, wetland wildlife, and associated ecological services.^304^, ^617^, ^618^ Stronger state and federal protection laws are critical to effective wetland preservation, and the recent U.S. Supreme Court decision in *Sackett v. U.S. Environmental Protection Agency* removes federal protection from wetlands that lack a visible connection to permanent surface waters, thus posing further challenges to wetland protection.^619^ New York State has begun to address this need with legislative updates to the Freshwater Wetlands Act that will protect more than 1 million acres of wetlands^620^ that are currently unmapped and smaller wetlands of unusual importance.^621^ Passing strong local laws that protect wetlands at the municipal level is another key adaptation for preserving existing, functional wetlands. Wetland resource mapping and prioritization tools are easily accessible online, along with model local laws for wetland preservation. The Natural Areas Conservancy and the New York City Department of Parks and Recreation have developed a Wetlands Management Framework for New York City to protect and manage existing wetlands, create new wetlands, allow space for wetlands to migrate as sea level rises, improve access to wetlands, and promote stewardship.^622^

To offset climate impacts, wetland best management practices such as revegetation, enhancement of buffers, planned retreat of coastal wetlands, and nutrient control will be of increasing importance in maintaining critical habitat.^623^ Areas managed specifically for wildlife (e.g., state wildlife management areas and national wildlife refuges) that use managed wetland impoundments may need to review sizing and placement of infrastructure (e.g., culverts and water control structures) and to ensure that the method and timing of water level management account for changes in precipitation, extreme storm events, and phenology of target species. There are significant opportunities to create wetlands such as vernal pools and wet meadows and to restore degraded wetland areas including forested wetlands and salt marshes. An important consideration for wetland creation and restoration projects is to ensure that the project design integrates projected changes in precipitation, temperature, and storm events due to climate change.^624^ These variables affect the hydrological model that often serves as a basis for the creation and restoration of wetland systems. Using updated models will increase the success of wetland creation and restoration attempts.

### 4.5 Riverine ecosystems

Among the most vulnerable riverine ecosystems are creeks and rivers that lack connected floodplains, forested buffers, or contact with groundwater and headwaters. Extreme climate events and seasonal changes in hydrology result in substantial impacts to these systems. Heavier precipitation events will leave all watersheds vulnerable to flooding and nutrient pollution from runoff and water treatment overflows. These and other vulnerabilities can apply to rural, agricultural, or urban watersheds, but typically intensify in the lower reaches of a river system.

Adaptations that can help address vulnerabilities in riverine ecosystems include implementing best management practices, managing invasive species, and closing knowledge gaps associated with the needs of aquatic fish populations and other aquatic species.^310^, ^625^ These adaptations are discussed in more detail below.

Best management practices are critical to the protection of riverine systems and the services they provide, including water quality protection and flood attenuation. For example:

- Agriculture and riverine ecosystems are closely connected and subject to compounded climate impacts such as catastrophic floods that simultaneously damage farmlands and pollute streams and rivers.^626^ Collaborative floodplain management can ameliorate both impacts.
- There is an increasing need to manage stormwater in the context of the hydrologic changes wrought by urbanization, including both flooding and “hydrologic drought.” In such cases, restoring natural flows and floodplains will increase capacity for flood management, while also improving habitat features and even aiding socioeconomic revitalization.^321^
- Higher nutrient loads driven by heavier precipitation point to the need to reduce downstream pollution that produces HABs^627^ and threatens drinking water systems with toxins and dissolved organic carbon loads.^628^ This challenge can be met with two well‐recognized adaptations. First is the effort to manage nutrient loading (agricultural, septic, and sewage), and second is the enhancement and restoration of riparian buffers, which capture, retain, and modify runoff constituents.^629^ Buffers also provide critical habitat and shade for narrow channels.
- Potential adaptations for warming waters include breaching or removing any legacy dams that have contributed to increased downstream water temperature. This also improves drainage and opens migration paths for native fish and invertebrates.^630^
- Adaptations for saltwater intrusion into estuaries and marine tidal creeks include improvements in the quality of proximal habitat to ensure that native species can relocate according to their tolerances.^631^

Climate adaptation efforts also require invasive species management. As described in Section 3.9.4, changes in climatic conditions such as temperature, precipitation, and the frequency and intensity of storm events can cause ecosystems, including riverine systems, to become more susceptible to invasive species colonization. A coordinated early detection/rapid response system can help protect the biodiversity of riverine systems.

Riverine ecosystem management is limited by several knowledge gaps. Many aquatic organisms are poorly studied, and professionals have an imperfect understanding of how to secure and manage their natural habitats.^632^ For those that are better studied, new considerations are emerging. For example, one study found that extreme precipitation events can have stronger impacts on fish populations when they occur during spring spawning periods.^633^ Exploration of knowledge gaps will help overcome current limitations in the ability of managers to respond to future demands of ecosystem protection. Decades of demographic modeling of fisheries, collection of aquatic invertebrate indicator species, and mapping the spread of invasive species have produced rich data sets and networks of skilled professionals, but a broader knowledge base is required to address the compounding impacts of climate change. More robust scientific guidance can assist with overcoming (1) an inadequate body of research on thermal tolerances of native fish and other aquatic animals; (2) uncertainties over future water levels and salinity profiles in estuaries and tidal creeks (e.g., an uncertain migration of the salt wedge in the Lower Hudson River estuary^634^; (3) untested capacities to restrict movements of aquatic invasive species between water bodies (e.g., preventing invasive fish from passing through the Champlain Canal into Lake Champlain^635^; and (4) a lack of interdisciplinary communication between researchers, such as those engaged in urban water management and those engaged in riverine ecosystem science.^636^, ^637^

### 4.6 Marine and coastal ecosystems

Climate change is already producing multifaceted impacts on the physical, chemical, and biological composition of marine and coastal ecosystems, such as enhanced warming from shifts in the Gulf Stream and increasingly unfavorable conditions for shellfish.^337^ Management of these ecosystems is challenged by the changes already occurring and those that lie ahead. Altering species and habitat use, establishing or reestablishing particular habitats, and improving interhabitat connectivity are all important management practices for achieving resilience. New York State has already established critical monitoring programs (e.g., the New York City Department of Environmental Protection Citywide/Open Water Long Term Control Plan program),^337^, ^638^ protection plans (e.g., the New York Ocean Action Plan),^339^ and cross‐sector interstate partnership infrastructure (e.g., the New York−New Jersey Harbor and Estuary Program)^639^ to enact necessary adaptive strategies and nimble management changes, and these programs will become increasingly important as species composition and habitat quality and availability continue to change. Specific adaptive management strategies for specific impacts may become more necessary in individual locations.

#### 4.6.1 Monitoring

Robust monitoring is necessary for ensuring adaptive management of harvests and resource use in marine and coastal ecosystems. Several examples of monitoring programs exist in New York State, including the New York Bight Whale Monitoring Program^640^ established by NYSDEC and New York Natural Heritage Program to standardize surveys in collaboration with other organizations (e.g., Stony Brook University School of Marine and Atmospheric Sciences); an Annual Report for New York Bight ocean ecosystem monitoring program run by the School of Marine and Atmospheric Sciences at Stony Brook University to monitor ocean indicators^641^; and various other federal, state, and county agency monitoring programs (e.g., those run by the National Oceanic and Atmospheric Administration Northeast Fisheries Science Center, NYSDEC, Suffolk County, and the New York City Department of Environmental Protection). However, sustained and increased monitoring of species, chemistry, and changes in marine and coastal ecosystems requires adequate resources. For example, only recently have the already‐experienced increases in acidification and deoxygenation been systematically documented, partly due to their punctuated and temporally disparate nature.^383^ Similarly, seasonal variations in species life cycles and interactions could necessitate changes to the timing of critical management interventions, which will require ongoing observations. With better data on ongoing changes, communities will be better prepared and equipped to adapt.

#### 4.6.2 Infrastructure

Infrastructure changes are another type of adaptation that can help address climate change impacts to New York State's marine ecosystems. Strategies for gray infrastructure (i.e., hard structures) and green infrastructure (i.e., plant and natural structures) are advancing and can contribute to addressing both the physical impacts of climate change, such as sea level rise, and the biological impacts, such as habitat loss. Because of the permanence of many of these infrastructural management strategies, decisions about their application should be based on extensive information, and careful consideration should be given to both beneficial and detrimental effects. For example, armored shorelines have become popular as stabilizing features, but their impacts underwater and downstream can destabilize important ecosystems. By redirecting wave energy, armoring can encourage scouring of seafloor sediments, and by trapping sediment, can stop the natural replenishment of beaches elsewhere along the shore.^642^

On‐land infrastructure can also help protect marine ecosystems from the impacts of climate change.^643^ Improved sewage management infrastructure that reduces combined sewer overflow will decrease the potential for eutrophication as sea level rises and storm intensity grows. Development with hard infrastructure set back from the shore will allow room for marsh migration. Additionally, the establishment of green infrastructure that buffers against storm surges and high sea level will provide a trap for contaminants, nutrients, and other pollution that otherwise would end up in waters, while also buffering coastal‐adjacent lands from increased salinity as sea level rises. A number of such green infrastructure projects have already been installed in coastal New York State and New York City, and more are planned.^644^, ^645^

#### 4.6.3 Management

Specific management strategies for individual habitats can be developed to improve the resilience of those habitats to climate change. For example, as changing climate conditions cause phenological changes (such as impacts on species life cycles and the timing of events such as migration and mating), ecosystem managers may need to change the timing of certain activities, such as fishing and dredging. Managers may also begin employing other strategies in response to climate‐related changes in local conditions. For example, as eutrophication and acidification increase, responses such as the development of seagrass beds^646^ or the release of alkalinity via methods that increase mineral weathering^647^ could become increasingly common.

#### 4.6.4 Connectivity

Within and between marine and coastal ecosystems, improving interhabitat connectivity will remain a primary method for increasing species and ecosystem resilience. While this principle holds true for most species and ecosystems, it is particularly important to many marine and coastal species because they use different habitats for specific life cycle stages, and connectivity across and between such habitats is critical to maintaining habitability across the marine and coastal system. This connectivity also enables adaptive strategies like migration and regime shifts. Connectivity makes it possible for species to retreat to refugia when conditions are not suitable, increasing their chances of survival. Over the past decade, many connectivity projects have been completed, including dam removal and culvert replacement upstream from the New York−New Jersey Harbor Estuary.^351^ Improved numbers of important species such as the American eel have followed those connectivity projects, highlighting their utility for ecosystem resilience.

### 4.7 Native flora and fauna

New York State's native plants and animals are affected indirectly by climate‐related changes in habitat, but also directly by increasing temperatures, changing precipitation patterns, and rising sea level. Additionally, increases in the frequency and intensity of extreme events such as storms can alter habitat and fish behavior, impacting fisheries,^648^ and an increase in marine heat waves can result in thermal displacement of species.^649^ As noted in Section 3.9.3, particularly vulnerable species include those at the southern edge of their range, alpine species, coastal species, those with strict interdependencies with other species, and those with limited mobility. The major challenge of climate change for these species is that it exacerbates the multitude of stressors that they already face—habitat loss and fragmentation, pollution, invasive species, and human intrusion, among others.^80^, ^650^ Thus, the top recommendation for reducing the effects of climate change on biodiversity is to continue to reduce stressors acting on wildlife and plant populations *right now*. For some species, these stressors are climate‐related, but for many, threats such as habitat destruction are more immediate and pressing. Animals and plants may be able to better withstand the effects of climate change if other stressors are reduced or eliminated.

Maintaining and restoring habitat connectivity has long been a top priority for wildlife conservationists seeking to counter habitat fragmentation caused by roads and development, and this strategy has only increased in importance with climate change.^80^, ^651^ Future temperature and precipitation regimes in the southern portions of species’ ranges could prove unsuitable for survival and reproduction. Facilitating movement to new habitat—and facilitating gene flow with nearby populations, especially those north and upslope—will be critical. At‐risk species are affected by habitat fragmentation, and these species will benefit from improved habitat connectivity even if their ability to adapt in place is greater than expected. Connectivity conservation comes in many forms and occurs at many scales^652^; it could include improving culverts and removing dams to allow the passage of aquatic organisms, installing bridges and wildlife underpasses to reduce highway mortality, and taking landscape‐level actions such as protecting large forest blocks. Conservation organizations such as The Nature Conservancy have developed mapping tools to assist with prioritizing landscapes for protection to maintain ecosystem connectivity.^586^ Application of the climate velocity concept has shown potential for informing such conservation strategies in the northeastern United States,^653^ as overlaid maps of climate velocities with biodiversity assessments help identify priority areas that can improve or maintain habitat connectivity.^654^ However, practitioners must take care to ensure that their efforts are not facilitating the spread of invasives or disease.

The Habitat Continuity Efforts case study gives examples of local and regional connectivity efforts. In certain extreme cases where the need for long‐distance migration (into, out of, or within New York State) is clear but the barriers are impassable or the species lacks the mobility to keep up with changing climate conditions, conservation practitioners may wish to consider assisted migration.^655^, ^656^, ^657^ This strategy, also called managed relocation, involves moving individuals to locations where the changed climate is more favorable. It is a controversial strategy among scientists and ethicists due to its risk of spreading diseases and invasives, as well as its unknown impact on other native species in the new location. The idea is so new that long‐term data sets on successes, failures, and corollary impacts have yet to be generated; however, some argue it is the only hope for certain species.^655^, ^658^

Some species could leave New York State no matter what conservation actions are implemented. Empirical evidence and models suggest that several decades from now the temperature could be too hot, the rain too sporadic, or the storms too intense for certain species to persist in the state. Most species expected to leave are already rare and threatened by other factors. Debates are occurring in the conservation literature about when it is appropriate to apply concepts of “triage”—choosing to spend resources on species most likely to persist.^659^, ^660^, ^661^ Triage concepts may be relevant at the state level, but they are highly controversial. Is it acceptable to lose a species from New York State if it is stable in other parts of its range, such as New England or Canada? And how certain are scientists and resource managers that species will respond in the ways that models predict? Could some species be more capable than expected of adapting to climate change? As always, management agencies must make difficult choices when choosing how to spend limited resources. Uncertainties like these, combined with the certainty that at least some changes in species composition are coming, have led some commentators to suggest that resource managers should focus on the conservation of geophysical conditions that promote resilience and diversity—either as an alternative to or as a supplement to species‐level conservation.^662^, ^663^, ^664^, ^665^ The Nature Conservancy has developed a suite of data sets to facilitate the conservation of resilient landscapes.^666^ Conserving known climate refugia—“safe havens” where species can remain relatively buffered from climate‐related impacts—is a useful strategy, regardless of conservation philosophy.^80^, ^667^, ^668^

It is clear that biodiversity monitoring is a beneficial investment under climate change. Monitoring plant and animal populations, distributional change, habitat associations, and movements will allow for adaptive management as the effects of a changing climate play out in real time. Without past monitoring, the scientific community would know far less about declines in boreal birds, loon physiology and population dynamics, the response of seabeach amaranth to rising sea levels, and the prospects for moose remaining in New York. Keeping a close eye on plants and animals of concern will allow resource managers and policymakers to act swiftly to encourage their continued presence in the state.

### 4.8 Urban ecosystems

Urban ecosystems are uniquely vulnerable to the effects of a changing climate. Urban ecosystem residents (human and otherwise) in New York State must cope with such impacts as amplified heat waves via the urban heat island effect, increasing runoff from precipitation, storm surges in coastal regions, and the spread of harmful invasive species. However, climate change also provides opportunities for creative adaptive management of urban ecosystems, because nowhere else are so many human lives dependent on the services provided by their local ecosystems. Urban resilience can be defined as the capacity to sustain functioning ecosystems and human well‐being, including the ability to respond to climate change by buffering, adapting, or transforming urban ecosystems.^669^ Urban conservation and restoration programs can recruit large groups of volunteers to implement resilience strategies and all‐important follow‐up monitoring. In addition, many cities have government agencies and nonprofit organizations with expertise in land use planning, project management, horticulture, arboriculture, and wildlife management, along with other skills necessary to cope with the new demands of urban ecosystem design and management.

Urban residents in New York City and across the state continue to invest in green infrastructure or nature‐based solutions, such as urban gardens; street trees; rain gardens; green roofs; and the protection of urban parks, forests, and waterways.^19^ Managing urban trees more broadly as forests by applying silvicultural management approaches could further enhance the benefits and ecosystem services that these trees provide, including reduction of air pollutants, energy savings, and enhanced infiltration of precipitation.^71^, ^670^ Urban trees are especially vulnerable to pressures from climate change, and planting new species that are better adapted to the evolving climate could be a better long‐term adaptation strategy than planting current native species.^671^

Environmental justice considerations are especially critical in urban areas because climate impacts are felt more strongly in low‐income communities, and current green infrastructure in U.S. cities disproportionately benefits high‐income neighborhoods.^419^, ^420^, ^672^ In addition, urban residents who rely on parks, ponds, community gardens, and remnant natural areas to enrich their lives and reduce climate hazards have uneven access to planning and decision‐making for urban ecosystem preservation and enhancements.^420^ Thus, a major challenge for resource managers seeking to design resilient urban ecosystems is to ensure that all residents benefit. Strategies focused on greening of abandoned properties and repurposing of abandoned or obsolete transportation corridors into parks are examples of ways to reverse the existing disparity. However, caution is warranted because highly attractive nature‐based solutions can lead to gentrification and reinforce unequal access to ecosystem benefits.^420^

The fates of urban ecosystems are inextricably linked to their dense and diverse human populations. One team of researchers recently proposed a set of six traits to serve as guiding principles for urban ecosystem resilience planning, each of which incorporates nature‐based and socioeconomic solutions, recognizing that their integration is necessary for successful climate change adaptation (Table 5‐3).^673^ All six traits—diversity, redundancy, connectivity, modularity, regenerative ability, and equitability—are fundamental, well‐tested principles or strategies in conservation ecology that are given new meanings in an urban ecosystem context.

**TABLE 5‐3:** Six guiding principles for urban ecosystem resilience planning.

	Diversity	Redundancy	Connectivity	Modularity	Regeneration	Equitability
**Definition **	Variety	Similarity and overlap among functions	Linkage among components	Independence and portability	How rapidly functions are renewed	Broad representation
**Purpose **	Facilitates adaptation	Insures against loss	Promotes dispersal and migration to minimize loss	Facilitates recovery after damage; limits spread of harm	Promotes recovery from impacts	Increases likelihood of success
**Ecological measures **	Species richness; mixture of adaptive traits	Number of similar species within functional groups; multiple components that provide the same ecosystem service	Habitat corridors; linkage to adjacent natural areas	Autonomy of components	Reproductive, growth, and migration rates	Broad and even spatial distribution
**Sociological measures **	Cultural, ethnic, and organizational variety	Similar or overlapping missions among environmental organizations	Communication to achieve shared goals	Autonomy of environmental organizations	Participation by a wide age range; youthful representation	Environmental justice considerations

*Note*: Table adapted from Reynolds et al.^673^

Biological diversity in the urban context includes functional diversity such as how much tree leaf area is available for seasonal heat mitigation.^421^ Redundancy in the urban context refers to the need to preserve key ecosystem functions (e.g., fruit‐bearing trees) so that the loss of one or a few species does not lead to loss of functions. Social redundancy ensures that many voices are heard from a variety of environmental and community organizations that share overlapping goals.^673^ Urban ecosystem connectivity includes greenspace corridors that link to natural areas outside of the urban setting and allow species migration, a critical need in a changing environment.^674^ Connectivity also includes facilitation of communication to achieve shared goals among stewardship groups.^673^ Modularity in the urban context refers to the independence and portability of green infrastructure components, which serve to limit harm and promote recovery following a damaging climatic event. Repurposing vacant lots for urban agriculture provides an example of modularity that has both an ecosystem context and a social context.^675^ Regeneration is a measure of ecosystem resilience, such as the capacity to recover from a wildfire or drought. Regeneration of urban ecosystem communities includes their capacity to maintain and rebuild multigenerational populations in relatively harsh conditions.^670^ Regenerative social capacity is indicated by the participation of community members representing a wide age spectrum. Finally, equitability refers to a balance of species occupancy, such that no species are exceedingly rare. In the social context, equitability reflects broad representation across multiple ecological and cultural communities to increase the likelihood of success, with special attention to frontline communities that lack economic and political influence.^420^

To date, green infrastructure has largely focused on stormwater management to minimize pluvial flooding, an evident climate change impact, with concerted efforts in New York City and Syracuse.^676^ The engineered network for managing stormwater contributes to other forms of ecosystem resilience—for example, when green infrastructure, such as detention ponds and constructed wetlands, supports urban biodiversity.^643^ Green infrastructure, however, is rooted in landscape ecology that considers a broad array of ecosystem services and has a long history of implementation in the United States.^677^ Integrating a landscape concept into green infrastructure planning provides a broader and more equitable set of adaptive outcomes than those realized from stormwater management alone.^678^ Experience with ocean storm surges, as during Superstorm Sandy,^679^ has led to a managed retreat from shorelines in New York City, providing opportunities to restore more natural coastal ecosystems.^680^, ^681^ Many states and localities are facilitating managed retreat in a proactive manner through setback regulations, zoning, and land acquisition.^682^ However, there is a risk that managed retreat will exacerbate social inequity.^683^ In contrast, engineered projects to increase resilience to sea level rise and storm intensification have been proposed, such as a gated storm surge barrier to protect New York City.^684^ In another example, a land‐based storm surge barrier is currently under construction on the east side of the city.^685^

### 4.9 Indigenous lands

Indigenous communities in New York State play an active role in climate change assessment and adaptation. For example, the Saint Regis Mohawk Tribe published a climate adaptation plan in 2013 that evaluated the impacts of climate change on the resources, assets, and community of the Mohawk Nation Territory (Akwesasne) and recommended adaption actions.^686^ Likewise, the Shinnecock Indian Nation published a climate vulnerability assessment and action plan in 2019 in cooperation with the Peconic Estuary Partnership.^687^ The environmental departments of both Tribes continue to work on existing issues and plan for a future affected by climate change.

Other Indigenous Nations also have active environmental departments that work to address climate impacts and other ecosystem stressors, often in collaboration with partners from government, academia, and the nonprofit sphere. Several Nations are part of the Haudenosaunee Environmental Task Force.^688^ For decades, Haudenosaunee leaders have been advocating at the national and international levels for humanity to address environmental issues, including climate change. With a long history of demonstrating resilience through major changes to their lifeways and homelands, Indigenous Nations have unique concerns and perspectives regarding climate change. Federal, state, and local officials can gain valuable insights from these concerns and perspectives through consultation and collaboration and incorporate them when developing mitigation and adaptation strategies and policy.

Examples of specific adaptation measures used by Indigenous Nations include:

- **Ecosystem restoration and conservation**. Indigenous Nations across New York State are working to restore and conserve ecosystems of all types, from wetlands to forests, as a way of increasing resilience in the face of climate change. The Tuscarora Nation has restored about 80 acres of grassland, replacing mostly invasive cool‐season grasses with native warm‐season prairie grasses that store carbon more efficiently and help regenerate soils.^689^ The Saint Regis Mohawk Tribe participated in a collaborative multiyear effort with the State University of New York Ranger School and other partners to increase forest health and production in black ash habitat.^686^
- **Invasive species management**. Responding to the likelihood that changing climate conditions will lead to the spread and increased survival of invasives, several Indigenous Nations are working to actively manage existing problems. The Saint Regis Mohawk Tribe has undertaken efforts to control invasive species such as purple loosestrife and the common reed, working in collaboration with organizations such as the Bureau of Indian Affairs’ Noxious Weed Eradication Program and Cornell University.^686^ The Shinnecock Indian Nation has assessed managing invasives such as the southern pine beetle through active forest management strategies, including restoring wildlife habitat and native vegetation and controlling overgrazing.^687^
- **Shoreline stabilization**. To address threats from sea level rise and coastal erosion, the Shinnecock Indian Nation is using a nature‐based approach to shoreline stabilization that involves deploying natural materials such as wetland plants, oyster reefs, sand, and stone.^687^ This type of “living shoreline” provides flood and erosion protection while also creating habitat for aquatic and coastal species such as crabs, shellfish, and flounder. As described in the Shinnecock Nation Marine and Land Farming Adaptations case study, the Nation restored 3000 acres of shoreline using a living shoreline approach, working collaboratively with Cornell Cooperative Extension and Suffolk County.^687^
- **Green infrastructure**. The Onondaga Nation has worked with county officials and other partners to apply green infrastructure solutions to prevent combined sewer overflows into Onondaga Creek, a tributary of Onondaga Lake.^690^ The Shinnecock Indian Nation's adaptation plans call for the use of bioretention basins, rain gardens, and other types of green infrastructure that promote stormwater infiltration as a way to remove pollutants from stormwater before it moves into groundwater and sensitive coastal habitats.^687^

Indigenous Nations can face unique challenges due to the misalignment of their ecosystem management priorities with those of federal and state environmental agencies.^78^ These challenges result from the fact that climate change adaptation priorities identified for broad ecosystem types do not always match the adaptation needs for smaller parcels of Indigenous‐held land. In addition, Indigenous Nations might seek to prioritize vulnerable species that are of cultural importance to them but not to others.

Adaptation and resilience initiatives often produce opportunities for cross‐jurisdiction partnerships between Indigenous Nations and federal and state agencies. Mutually respectful relations, research, and knowledge sharing are key to developing climate change adaptation strategies and actions that benefit all while remaining mindful of the unique political status, contemporary contexts, and environmental perspectives of Indigenous Peoples. Federal agencies are guided by executive memoranda that call for government‐to‐government, nation‐to‐nation engagement with Indigenous Nations. In 2021, the White House Office of Science and Technology Policy and the White House Council on Environmental Quality jointly released a new memorandum that commits to elevating Indigenous Traditional Ecological Knowledge in federal scientific and policy processes.^691^ NYSDEC maintains a policy of government‐to‐government relations with Indigenous Nations and has an Indian Nations Affairs Coordinator.^440^

### 4.10 Invasive species

All ecosystems in New York State are prone to further outbreaks of exotic and neonative invasive species, with climate change as a cofactor in their impacts. Agricultural and aquacultural ecosystems are relatively confined, so containment of invasive species is often possible, but climate change will stress cultivated species and render them more susceptible to pests and diseases. Natural ecosystems do not permit a similar level of containment, and this leads to a management dilemma. The dilemma is that there are two competing needs for biological conservation in the context of changing climatic conditions.^692^ The first is a need to provide more connectivity between wild lands and waters to allow migration of species sensitive to warmer and more variable climates. The second is a need to prevent the spread of invasive species, which could require setting or reinforcing barriers to movement. The expansion of native species, which may or may not have positive impacts on ecosystems, will add challenges to land management decisions.

New York State is nationally recognized for leadership in invasive species management and has already developed supporting resources for climate change adaptation, including networks of public and private partnerships, bipartisan legislative mandates, and stable state funding. The PRISM network (described in Section 3.9.4) is overseen by a new unit within NYSDEC and is assisted by a clearinghouse for research, the New York Invasive Species Research Institute. Comparable efforts are growing in nearby states and provinces, which share a deep interest in the impacts of climate change and recognize the need to collaborate. The next step will be defining how to grow and deploy the expertise required to guide future invasive species management. An effective management program requires a strong invasive species research portfolio with advanced tools for anticipating and detecting the arrival of new invasive species; improved predictive modeling that employs climate change forecasts; and more comprehensive impact assessments, such as quantification of the broad environmental and economic impacts of invasive species. Equally important is the need to increase education and outreach at all levels, with further engagement of community volunteers in invasive species monitoring and management, as well as spreading of public awareness. Following invasive species management activities, restoration decisions will benefit from the incorporation of climate considerations to ensure ecosystem resilience against the combined threat of invasive species and climate change. At higher levels of governance, new measures and policies can be enacted to prevent introductions of problem species (e.g., introductions of forest pests via international commerce)^186^ and to keep out invasives that are likely to expand their ranges into New York State under future climatic conditions. Finally, climate change response plans can incorporate funding and strategies for early detection and rapid response to new invasions facilitated by extreme events and warming temperatures.

### 4.11 Resources and support for ecological adaptation and resilience strategies

The resilience of human communities depends critically on the functional integrity of ecosystems. Protection, restoration, and connection of ecosystems are adaptation measures that not only can reduce the impacts of climate change on ecosystems but also can increase resilience for human communities and infrastructure. Adaptations that increase the resilience of ecosystems are integral to broader climate resilience, and support agriculture, infrastructure, society, and health. As part of a broader strategy, ecosystem adaptations tend to be cost‐effective and produce greater equity (i.e., benefit more people) than engineered, site‐specific solutions.

In recent years, there has been growing interest in and support for climate resilience approaches that include ecosystem strategies (nature‐based solutions). There has also been an increase in available resources. It is important to recognize and build upon these resources and activities to energize and inform future action at all scales.

#### 4.11.1 Federal resources

The National Fish, Wildlife, and Plants Climate Adaptation Strategy was developed in 2012 by an intergovernmental working group of federal, state, and Tribal agency representatives and then updated in a 2021 review.^80^, ^650^ The strategy provides a framework of responsible actions that can be taken by natural resource managers, conservation partners, and other decision‐makers at all levels. The strategy includes eight adaptation goals. Broadly, these include: (1) conserve and connect habitat; (2) manage species and habitat; (3) enhance capacity for management; (4) support adaptive management; (5) increase knowledge and information; (6) increase awareness and action; (7) reduce nonclimate stressors; and (8) include local communities.

The Federal Emergency Management Agency and the U.S. Environmental Protection Agency developed a local assessment tool titled Resilience Implementation and Strategic Enhancements (RISE).^693^ This tool has an entire focus area dedicated to conserving ecosystems in critical coastal areas, river corridors, and other flood‐prone environments.

#### 4.11.2 State resources

At the state level, New York has advanced several policies and programs to help support and fund climate adaptation, including some that employ ecosystem management strategies and nature‐based solutions. For example, the Community Risk and Resiliency Act of 2014,^694^, ^695^ which was amended by the 2019 Climate Leadership and Community Protection Act (Climate Act),^696^ integrates consideration of future physical climate risk due to sea level rise, storm surge, and flooding into project designs, siting regulations, permit reviews, and smart growth criteria for public infrastructure.^594^ The Act required state agencies to develop guidance and model local laws^594^, ^697^ to facilitate community efforts that use natural resources to enhance resilience.

Examples of state resources include model laws for wetland and watercourse protection measures and a 2020 guidance document called Using Natural Measures to Reduce the Risk of Flooding and Erosion.^698^ Climate Smart Communities ^699^ is another New York State program that offers technical assistance and funding for local governments to take climate action and adaptation measures, including nature‐based solutions. State funding cycles include grant monies to fund the implementation of resilience tools. NYSDEC has a growing set of resources to assist communities with climate resilience. These include the Resilient NY program,^700^ which provides resources to improve community resilience to extreme weather events, and the Hudson River Sustainable Shorelines project,^701^ which facilitates nature‐based shoreline management. In addition, New York State's Soil Health and Climate Resiliency Act^702^ recognizes and incentivizes growing opportunities for improved agricultural practices that reduce ecosystem impacts and improve environmental health consistent with productive farms.

These are just a few specific examples of state resources. There are many others that help to support climate resilience projects, from the establishment of community forests to the implementation of green infrastructure solutions for stormwater management. The website for New York State's annual Consolidated Funding Application process provides a comprehensive list of relevant resources and opportunities for climate adaptation.

#### 4.11.3 Local, regional, and tribal resources

It is critical to include local communities and local knowledge in adaptation efforts. An assessment of climate change impacts on ecosystems and ecosystem services reveals that some segments of the state's population (and, in some cases, entire communities) have specific and significant vulnerabilities. All humans are inextricably linked to natural ecosystems, but some are more intimately connected with and dependent on ecosystems and the natural resources they provide. Many of these people have deep‐seated knowledge and skills pertinent to ecosystem management and stewardship, but they lack political and economic influence that would allow them to contribute their expertise. This can be seen as a form of indirect vulnerability, which limits the capacity of many communities to cope with direct hazards of climate change. Examples include urban residents, shoreline inhabitants, and rural and Indigenous communities. The needs, concerns, knowledge, and efforts of these communities are a critical component of climate resilience.

Tribal Nations, municipalities, local organizations, and nonprofits are developing climate adaptation and resilience tools and decision‐making frameworks. The Shinnecock Indian Nation has developed a climate change adaptation plan that includes action recommendations to address coastal shoreline erosion, flooding from sea level rise, and reestablishment of traditional food systems and community farming. Several counties are developing their own resilience plans that help municipalities understand climate impacts, conduct asset vulnerability and risk assessments, and develop strategies to increase climate resilience and equity, including nature‐based strategies (e.g., refer to the Genesee County Phase II Resiliency Plan). The Nature Conservancy developed an online Resilient Land Mapping Tool that allows users to identify connected networks or potential networks of resilient, biodiverse lands that are projected to be buffered from climate change impacts to species. The Western New York Land Conservancy has launched the Western New York Wildway ^703^ initiative to connect important forest ecosystems from Pennsylvania to the Great Lakes. Many municipal resilience strategies are supported by state funding programs discussed above. These include the New York State Department of State's Smart Growth program and NYSDEC's Water Quality Improvement Project.

### 4.12 Aligning adaptation with greenhouse gas reduction

Ecosystems will benefit if strategies to reduce greenhouse gas emissions are integrated with climate adaptation strategies. For example, reducing carbon dioxide emissions at the state level, a goal of the Climate Act,^696^ will likely benefit ecosystems by reducing emissions of pollutants such as ozone. Ecosystems can also serve as a tool to reduce net emissions of greenhouse gases through strategic management practices. The role of ecosystems in reducing greenhouse gas emissions can take several forms:

- **Removing carbon dioxide from the atmosphere and storing it in ecosystem components,** including forest and marine biomass, soils, soil waters, and aquatic sediments. These “sinks” store carbon in organic or inorganic form, the former being carbon captured through photosynthesis and preserved in recalcitrant forms (i.e., less‐degradable woody biomass, aggregated soil organic matter, organic matter preserved in anoxic sediments in aquatic and marine ecosystems), and the latter being carbon dioxide converted into more stable, nongreenhouse gas carbon species (such as the mineral calcium carbonate).^704^, ^705^
- **Management efforts to reduce greenhouse gas emissions from ecosystems**. Examples include a growing emphasis on retention, rather than removal, of forest products (per the Climate Act), or the management of wetlands to reduce methane emissions.^616^
- **Hosting infrastructure for renewable energy production**. Just as waterways have long been used as a source of hydropower, ridgelines and offshore waters could provide sites for wind turbines, and unforested lands could be used for solar photovoltaic production. However, these opportunities can sometimes conflict with other land‐use management concerns.^706^

Using these measures to help New York State broadly achieve its ambitious Climate Act greenhouse gas reduction targets could also support or complement adaptation goals. Managing ecosystems to increase carbon dioxide capture and storage and other greenhouse gas reduction methods can also increase the value and resilience of those ecosystems—for example, by conserving forest habitat and associated biodiversity.^707^ Moreover, the voluntary markets and policy incentives that promote these management practices can in turn help individual, municipal, and private stakeholders finance adaptation and resilience, including in rural and Indigenous communities. While existing economic incentives largely focus on direct greenhouse gas‐related “offsets” such as carbon dioxide removal or emissions reductions,^708^ future policy and market incentives could grow for practices that provide other environmental/ecological services (broadly referred to as “payment for environmental services”), such as practices that increase or preserve biodiversity, or those that improve the availability or quality of water resources—particularly as climate change challenges these resources.^709^

Conversely, some greenhouse gas reduction strategies could conflict with adaptation and resilience priorities and with historical paradigms of ecosystem management. For example, increased interest in solar infrastructure on open spaces, including agricultural land and grasslands, often conflicts with other management of those spaces, such as for agricultural production, reforestation, park installation, or other development.^710^ Concerns have been raised that land‐based wind turbines can result in substantial mortality of birds and bats.^711^ Similarly, proposals for offshore wind infrastructure off the New York State coastline have engendered pushback from communities involved in the traditional management of those waters for fishing and shipping^712^ as well as for biodiversity protection^341^ and scientific research.^713^ In this sense, some responses to address climate change may themselves introduce stressors, both ecological and social, that affect future ecosystem management across the state. It will be important for policy and decision‐makers to consider potential conflicts or maladaptation. These impacts are most appropriately addressed by giving equal consideration to the benefits of land‐use practices, such as when solar farms replace the need for a power plant that burns fossil fuels, which may generate different but substantial impacts to vulnerable ecosystems.^714^

## 5 LOOKING AHEAD

Using ecosystem management tools to adapt to future climate change presents many opportunities—not only in the form of more resilient ecosystems and the direct services they provide, but also through a variety of cobenefits. At the same time, a balanced comprehensive assessment presents an opportunity to describe ways in which some ecosystems could benefit from climate change. This section summarizes these types of opportunities that lie ahead for New York State. It then identifies emerging topics and research needs that could further improve understanding of ecosystem impacts and future adaptation options. The section concludes with a review of the major findings presented in this chapter.

### 5.1 Opportunities for positive change

Changing climatic conditions could produce positive outcomes in some ecosystems or for some species in the state. In addition, the work of adapting to a changing climate and advancing ecosystem resilience can lead to many additional cobenefits. Examples of these opportunities include:

- **New habitat formation**. As the climate changes and sea level continues to rise, new habitat will form as existing ecosystems move further north, higher in elevation, or further inland (refer to Sections 3.1.3, BOX 7, and 3.8). These changes present conservation challenges but also opportunities to facilitate change through assisted migration of new tree species and the introduction of wetland vegetation to previously dry coastal areas.^297^, ^578^
- **Establishment of valuable tree species**. Climate change will expand the area of potentially suitable habitat for many southern tree species that are currently absent from or rare in New York. Though large‐scale shifts in species distributions are not expected in the near future, there will be opportunities to enrich forest stands and landscapes by purposefully establishing or encouraging the expansion of valuable tree species.^577^ Trees that could become more prevalent in the state include species that would be valuable to wildlife (e.g., hard mast producers such as oaks and hickories), species that would increase carbon uptake (tulip poplar and southern pines), species that would lead to high‐value timber production (walnut and oaks), and species that would enhance the aesthetics and scenic beauty of the landscape (flowering dogwood and redbud).
- **The rise of new fisheries**. Warming water temperatures off New York's coast will likely lead to the collapse of some fish populations and existing fisheries but could also present new opportunities for the state in the future. New fisheries could arise along with new economic and cultural opportunities (refer to Sections 3.5 and 3.8). As discussed in Section 3.8.2, commercially significant species such as summer flounder (fluke) and black sea bass have increased in abundance over the past decade as temperatures have warmed. The surge in blue crab populations along the coast of Long Island also brings the potential for a new commercial and recreational fishery, as described in the Shifts in Lobster and Crab Populations case study.
- **Cobenefits of ecological connectivity**. As described in the Habitat Continuity Efforts case study, efforts to address habitat fragmentation by improving connectivity not only benefit wildlife species by allowing them to shift their ranges in response to changing climatic conditions, but also benefit humans in numerous ways. For example, they reduce the likelihood of collisions with animals on roads and other conflicts between humans and wildlife. People also benefit from ecosystem services provided by large, unfragmented natural areas, including recreational and scenic value.
- **Cobenefits of preserving natural and working lands**. For many ecosystem types, the most effective method of offsetting the effects of climate change is preserving or improving the health and functionality of existing ecosystems through best management practices (refer to Sections Species preferring warmer conditions, BOX 14, and 4.10). This approach not only enhances climate resilience but also leads to other benefits. Healthy, functioning ecosystems can sequester and store carbon, reduce greenhouse gas emissions into the atmosphere, and protect people and wildlife from the impacts of climate change. Well‐managed wetlands, for example, allow for greater carbon storage and reduce risks of flooding and soil erosion. Incentives for “nature‐based” greenhouse gas reduction efforts could help fund many of these management practices.
- **Opportunities to build and strengthen multisector partnerships**. Growing awareness of the measurable effects and projected impacts of climate change can motivate and align efforts to increase climate resilience through nature‐based projects (e.g., reconnecting floodplains to reduce damage from an increase in extreme precipitation events). This can create new synergies for positive change and lead to the development of landscape‐scale ecosystem management projects supported by traditional partners (soil and water conservation districts, conservation agencies and organizations, municipalities) and nontraditional partners (private businesses and landowners, agribusiness, public health agencies, Indigenous communities). Section 4.11 provides examples of collaborative resilience efforts and resources. Such projects and partnerships can provide multiple community benefits, including reduced property damage and loss of human life, increased habitat for wildlife, reduced erosion of farmland soils, improved water quality, and increased opportunities for outdoor recreation and education.
- **Opportunities to address environmental justice concerns through the design of resilient urban ecosystems**. Efforts to reduce climate impacts on urban ecosystems (e.g., amplified heat waves, increasing runoff from precipitation) provide an opportunity to address long‐standing equity and environmental justice concerns (refer to Sections 3.9.1 and 4.8). Climate impacts disproportionately affect low‐income communities, where residents have historically had less access to existing green infrastructure. As resource managers and planners look to create resilient urban ecosystems through investing in and deploying green infrastructure and nature‐based solutions such as urban gardens, street trees, green roofs, and stormwater detention ponds, they can strengthen these projects by involving community members in planning and decision‐making to design solutions that benefit all residents and reverse existing disparities. As described in Section 4.8, urban conservation and restoration programs can also involve community members as volunteers to implement resilience strategies and follow‐up monitoring.

### 5.2 Emerging topics and research needs

Many issues that emerged during the development of this chapter could benefit from further investigation. In terms of next steps for research and policy development, areas of potential interest include:

- More comprehensively monitoring changes in ecosystem condition and composition in response to climate change across all geographic regions and ecosystem types.
- Integrating regional climate models for more geographically nuanced projections relevant to ecosystems.
- Integrating Traditional Ecological Knowledge into collective endeavors to protect ecological communities.
- Expanding research and advancing knowledge on topics such as:
  ∘ Thermal tolerances of native fish and other aquatic animals. Relatively few species have been characterized to date.
  ∘ The effect of extreme precipitation events during spring spawning periods on fish populations.
  ∘ The potential effects of sea level rise on salinity profiles in tidal creeks and estuaries and in groundwater, including in the Lower Hudson River estuary.
  ∘ Other impacts of sea level rise on coastal ecosystems.
  ∘ Phenological mismatches between species that are interdependent.
  ∘ The role of fungi in ecosystems for resilience and opportunities for carbon sequestration.
  ∘ The combined effects of a warming climate, increased atmospheric carbon dioxide concentrations, and nutrient availability on drought resistance, transpiration, and carbon sequestration in forest vegetation.
- Understanding, predicting, and mitigating cascading impacts and ecological tipping points.
- Improving interdisciplinary communication between researchers, such as those engaged in urban water management and those engaged in riverine ecosystem science.

### 5.3 Conclusions

Climate is a fundamental driver of the distribution and function of ecosystems. In New York State, a changing climate is measurably altering all types of ecosystems, from wetlands and forests to lakes, rivers, and coastal areas. Because the state's communities and economies depend on healthy, functioning ecosystems, climate‐induced changes have the potential to affect the quality of life for all New Yorkers. As described in Section 2.4, groups that are especially vulnerable to such changes include rural communities with natural resource‐dependent economies; Indigenous communities who depend on ecosystems for foraging, fishing, hunting, and cultural ecosystem services; and coastal communities sensitive to sea level rise.

This chapter examined the impacts of climate change on seven major ecosystem types in New York State: forests, open land, alpine ecosystems, lakes and ponds, wetlands, riverine ecosystems, and marine and coastal ecosystems. It also looked at cross‐cutting ecosystem topics, including climate impacts on urban ecosystems, Indigenous lands, native flora and fauna, and invasive species. Broadly speaking, the chapter explored two types of climatic changes that are affecting the state's ecosystems: (1) gradual changes in average conditions, such as increases in mean temperatures, total precipitation, or sea level; and (2) increases in the frequency of extreme events such as intense storms, floods, droughts, and heat waves.

The chapter highlighted impacts that result from gradually occurring changes in average conditions. For example, in aquatic environments, rising water temperatures lead to deoxygenation, cause stress for coldwater species, and contribute to intensified stratification in lakes (Sections 3.5 and 3.7). In marine and coastal waters, rising water temperatures and changes to circulation have cascading effects on the composition, range, and distribution of species, with warmwater species replacing coldwater species in some habitats (Section 3.8). In all types of New York State ecosystems, species sensitive to warming temperatures are at greatest risk of extirpation, as are those on the southern edge of their ranges.

Sea level rise is another gradually occurring change that has the potential to cause a range of devastating impacts, from habitat destruction and dangerous storm surges to saltwater intrusion into groundwater supplies. As discussed in the chapter, inundation from sea level rise is expected to lead to a loss of coastal wetlands, although marsh migration into upland areas could offset some of those losses.

While gradual changes in climatic conditions can have substantial effects over time, extreme climate events have a more severe impact and incur greater societal impacts and costs. As described in the chapter, a small riparian wetland can be buried completely by sedimentation during one extreme storm event. Extreme precipitation events can cause rivers to flood, alter their channel and floodplain geometry, and disrupt populations and communities of aquatic species.

The chapter emphasized that observed impacts are usually the result of climate hazards acting in combination with existing nonclimate stressors such as agriculture, development, and pollution. For example, compared with larger wetlands that still have a relatively high degree of hydrological and ecological connectivity, small wetlands fragmented by damaging land‐use patterns may be less able to recover from drought and other extreme events or from increased evaporation rates caused by warming (Section 3.6). Runoff from agricultural fields after extreme rainfall events can contaminate rivers and lakes with fertilizers and pesticides, enhancing the potential for HABs that can suppress native plants and animals (Section 3.7). In urban ecosystems, an increase in the frequency of large storms can lead to rapid runoff that exacerbates existing water quality concerns such as elevated levels of nutrients, sediment, trace metals, and trace organic pollutants in urban lakes, rivers, wetlands, and groundwater (Section 3.9.1). The chapter concluded that the interactions of climate and nonclimate stressors and the cascading or cumulative changes they produce pose the greatest risk in terms of the magnitude of impact.

Climate change also interacts with land‐use change to increase the potential for invasive species (Section 2.3). Ecosystem disturbances caused by development create opportunities for non‐native species to invade new ecosystems, as do disturbances caused by extreme weather events such as hurricanes and floods. Shorter, milder winters also allow for the survival and spread of invasive species once they have taken hold in an ecosystem. A drop in the number of freezing days allows new pests, such as tree‐feeding insects, to survive winter, and warmer average air temperatures will permit established pest populations to grow faster and larger (Section 3.9.4).

The chapter's final section discussed adaptation measures that can help moderate the effects of climate change on ecosystems. Across all ecosystem types, monitoring was identified as an important measure for understanding how climate change is affecting environmental conditions and plant and animal populations. Because avoiding or reducing nonclimate stressors, such as habitat fragmentation, is often easier and better understood than directly managing the impacts of climate change, the chapter also recommended prioritizing adaptation strategies that jointly address climate change and nonclimate stressors. In many of New York's ecosystems, the top recommendations are for using best management practices that increase ecosystem connectivity, resilience, and health so that natural systems will be better able to withstand the effects of changing climatic conditions (Section 4 and Habitat Continuity Efforts case study). For example, the impacts of climate change on marine and coastal ecosystems can be reduced with proactive ecosystem management that decreases runoff, protects shorelines, and improves habitat quality and connectivity (Section 4.6). In forest ecosystems, managers can use silviculture best management practices that promote structural and compositional diversity to foster climate resilience (Section 4.1).

Priorities for adaptation in urban ecosystems include investment in green infrastructure or nature‐based solutions such as urban gardens, street trees, green roofs, stormwater detention ponds, and constructed wetlands. The chapter highlighted the importance of prioritizing environmental justice concerns when considering climate adaptation strategies in urban areas. Managers and policymakers can develop more effective strategies by considering who is disproportionately affected by climate impacts and who will benefit from the solutions.

## 6 TRACEABLE ACCOUNTS

Traceable accounts examine each key finding in depth. They provide citations that support each assertion and present the authors’ assessment of confidence in each finding.

### 6.1 Key Finding 1

**Extreme climate events can have large impacts on New York State's ecosystems, and many types of extreme events are increasing in frequency and intensity as the climate changes**. Events such as intense storms, droughts, and heat waves disturb ecosystems as they harm soil, vegetation, and wildlife populations. Ecosystem management strategies focused on the impacts of extreme events will be helpful in preserving ecosystem services where achievable and can minimize the loss of future ecosystem functions.

#### 6.1.1 Description of evidence

Analyses of trends in weather data indicate that many types of extreme climate events (such as heat waves, hot days, and days with heavy precipitation) are increasing in frequency across New York State and the northeastern United States.^20^, ^21^, ^22^, ^23^ In contrast, trend analyses show that extreme events associated with cold weather (such as cold days) are decreasing in frequency.^20^, ^22^ Heat‐related extreme events in lakes parallel those on land.^83^ Chapter 2, New York State's Changing Climate, presents additional evidence of observed changes in extreme event frequency and severity.^162^ That discussion includes drought, noting relatively high confidence in projections of certain drivers of drought such as warmer summer temperatures and drying associated with earlier snowmelt, but also noting that climate models disagree on the direction of change in summer precipitation. Patterns in many types of extreme climate events are expected to persist into the future based on evidence from downscaled climate models, and the sharpest increases are expected with the highest emission scenarios.^715^, ^716^

The effects of extreme climate events have been demonstrated across a wide variety of ecosystems in New York State, including direct effects on organisms as well as effects on ecosystem services. Studies have shown that extreme events such as intense droughts, precipitation that causes flooding, and ice storms could increasingly affect forests in New York.^127^ At greatest risk are forests in coastal areas such as Long Island, where rising sea level combined with an increased risk of intense coastal storms poses an increased risk of tree mortality.^200^ Risks to riverine ecosystems grow as the frequency and intensity of large runoff events increase. Such events mobilize sediments and nutrients, erode stream banks, and damage riparian vegetation. Short‐term impacts include the promotion of HABs, and long‐term impacts include channel erosion and damage to floodplain trees from ice block collision, which is often due to unusually warm mid‐winter weather.^298^, ^319^, ^321^, ^323^ In marine settings, discrete but prolonged periods of excessive heat (lasting from weeks to months) may cause thermal displacement of species.^649^ In northern temperate lakes, periods of extreme heat have resulted in fish die‐offs,^239^ and large storms alter nutrient and light availability and several physical properties. These changes can have cascading impacts on the phytoplankton community, including loss of biomass, a decrease in blooms, and increased biodiversity.^266^ Increases in the frequency and intensity of large storms and droughts are expected to affect a range of functions across terrestrial, aquatic, and marine ecosystems, including productivity, provisioning services, and biodiversity. Technological advances in monitoring systems are expected to improve understanding of the impacts of extreme events in the coming years.^717^

#### 6.1.2 Assessment of confidence, new information, and remaining uncertainties

Chapter 2, New York State's Changing Climate, concludes that several types of extreme events are increasing or will increase in frequency and/or intensity as the climate changes.^162^ Based on that chapter, as well as the evidence presented within this Ecosystems chapter:

- Confidence is **very high** that heat extremes will increase in frequency and intensity during the 21st century.
- Confidence is **high** that several types of storms will become more intense during the 21st century. However, models disagree about projected changes in the frequency of storms, and there is countering evidence that indicates that variations in other factors that affect atmospheric circulation have affected recent trends in storminess.^24^, ^25^
- Confidence is **medium** that short‐term summer droughts will increase in likelihood or severity.
- Confidence is **medium** that future storms will result in ecosystem impacts that exceed those of the pre‐existing storm regime. One exception is coastal storms, where there is **high** confidence that future ecosystem impacts will exceed those of the past, driven in part by rising sea level.^372^

Compound extreme events can include any combination of heat waves, large storms, intense droughts, or other extreme conditions that occur closely in time. The study of compound extreme events is an emerging area of research, and ecosystem impacts from these events have been demonstrated in riverine and estuarine ecosystems in New York and nearby regions.^356^, ^357^ At present, there is **medium** confidence that compound extreme events will increase in frequency with climate change, but only **low** confidence that compound events pose a magnified risk to most ecosystems, with the notable exception of coastal ecosystems.^8^ Study of the ecosystem impacts of compound climate events that could be increasing in frequency as a result of climate change is an emerging area of research around the globe,^6^, ^26^ and worthy of increased attention in New York State.

### 6.2 Key Finding 2

**Rising water temperatures will have cascading effects on the composition, range, and distribution of species in New York State's waters**. Species adapted to cold water will seek more favorable habitat, species adapted to warmer water will move into previously colder habitat, and the physiological stress of warming will increase vulnerability to other stressors such as disease and invasive species. These changes are already occurring in lakes, rivers, wetlands, and marine and coastal waters. Adaptation strategies focused on identifying and maintaining coldwater habitats will benefit thermally stressed species in the coming decades.

#### 6.2.1 Description of evidence

The temperatures of rivers, lakes, and coastal waters in or near New York State have increased by 0.3−2°F per decade over the past few decades, varying by water body type and location.^27^, ^28^, ^29^, ^32^, ^33^, ^34^, ^35^, ^36^, ^718^ These warming trends contribute to deoxygenation, intensified stratification of lakes, and a lengthened growing season.^33^, ^35^, ^349^ Resulting ecological effects include fish die‐offs, migration of warmwater species into habitat previously occupied by coldwater species, shifts throughout food webs, and an overall increase in metabolic rates, which increases biological oxygen demand.^239^, ^256^, ^350^, ^353^ Warming water temperatures could also be contributing to increases in HABs in some water bodies.^37^, ^719^ In addition to long‐term warming trends, an increase in short‐term heat waves has been observed in marine and fresh waters.^83^, ^344^

#### 6.2.2 Assessment of confidence, new information, and remaining uncertainties

Confidence is **very high** that marine and fresh waters in New York State will continue to warm in future decades. Confidence levels vary regarding the physical effects of this warming, such as deoxygenation (**high** confidence), intensified lake stratification (**medium** confidence), and a lengthened growing season (**very high** confidence). There is **high** confidence that an increase in the frequency and intensity of short‐term heat waves will be observed in the state's marine and fresh waters. Confidence levels regarding the ecological effects of this warming vary as a function of water type, water depth, connectivity, and degree of mixing. For example, deeper lakes have coldwater refugia, and these deeper waters have either not been warming or are warming at lower rates than shallow waters. So, there is **low** confidence that a loss of coldwater species will occur in deep lakes, because coldwater species could adapt and move to deeper habitat; likewise, there is **low** confidence that coldwater species will be replaced by warmwater species dependent on natural connection to warming lakes. In general, replacement by warmwater species is highly variable. It is already occurring with **high** confidence in the coastal waters of Long Island (refer to Shifts in Lobster and Crab Populations case study), but there is little evidence of such changes at present in the lakes of New York. Facilitated migration of warmwater species may become a viable management adaptation strategy when these lakes can no longer support coldwater species. **Medium** confidence is assigned to the hypothesis that warming waters will play a growing role as a driver of nuisance problems such as HABs and eutrophication, which are dependent on other contributing factors such as nutrient availability.

### 6.3 Key Finding 3

**Human activities that degrade the environment continue to be more impactful to New York State's ecosystems than projected climate change impacts alone**. These nonclimate stressors include habitat loss and fragmentation, erosion, sedimentation, and pollution. The interaction of climate change and ongoing stressors associated with land‐use practices and land‐use change accounts for the most substantial projected ecosystem impacts. Avoiding, reducing, and mitigating nonclimate stressors is often more readily attainable than directly managing the impacts of climate change, indicating the benefit of jointly addressing climate change and nonclimate stressors in adaptation planning.

#### 6.3.1 Description of evidence

Climate change is one of several drivers of long‐term change in ecosystems. Other drivers include land‐use and sea‐use practices, harvesting of plants and animals, pollution, and invasive species.^720^ Taken together, these other drivers pose a greater threat to ecosystem health than does climate change alone. Compared with climate change, which is largely driven by global emissions, these other factors are also more readily managed to minimize ecosystem impacts, especially in the immediate future.^721^ For example, the principal cause of biodiversity loss globally is habitat loss resulting from factors such as urbanization and expansion of agricultural land that isolate and fragment landscapes.^54^, ^55^, ^721^ Nonclimate stressors generally relate to activities that serve human needs, including food and fiber. Management of these human activities, such as minimizing the ecosystem disservices associated with agricultural practices, will benefit efforts to address ecosystem impacts from climate change.^57^ Adaptation that minimizes one or more ecological impacts of climate change will typically minimize impacts not associated with climate change as well. Vernal pools, which serve as an important breeding habitat for amphibians in the Northeast, provide an excellent example of an ecosystem type that is undergoing fragmentation and loss not only because of climate change impacts, but also because of human activities such as road construction and land‐use change.^294^, ^304^, ^722^

#### 6.3.2 Assessment of confidence, new information, and remaining uncertainties

Confidence is **very high** that factors other than the direct impacts of climate change are currently the most important driver of ecosystem impacts across New York State. One exception is in open marine waters, where warming temperatures appear to be the dominant driver of ecosystem change; but even in marine systems, human factors (such as activities that enhance nutrient loads and concentrations) become increasingly important drivers of change close to the coast. There is **high** confidence that deleterious ecosystem impacts can be alleviated through adaptation strategies that address multiple nonclimate stressors as well as climate change.^55^ Failure to consider factors such as habitat loss and fragmentation will provide only **low** confidence in the success of current ecosystem adaptation strategies. New York State has been proactively developing adaptation plans for its ecosystems that consider both climate change and direct human impacts.^119^, ^339^ However, adaptation plans have not generally met implementation goals and have fallen short by being only incremental, which could be the result of insufficient funding and institutional constraints.^575^ Adaptation implementation can be improved through a more systematic approach to evaluating outcomes and impacts.^723^ Thus, only **medium** confidence can be assigned to the goal of holistically implementing ecosystem adaptation strategies that are comprehensive enough to meet the urgency of the moment.

### 6.4 Key Finding 4

**Sea level rise will substantially alter New York State's coastal and tidal ecosystems**. Coastal ecosystems will increasingly flood, and intrusion of salt water into areas previously occupied by fresh water will cause deleterious impacts to low‐lying coastal ecosystems. The extent of coastal wetlands will decline in many areas. However, the extent of some coastal wetlands may be maintained, and other coastal ecosystems may expand, if inland habitat is available for expansion—a resilience challenge that intersects with the built environment.

#### 6.4.1 Description of evidence

Sea level has been rising by up to 1.6 inches per decade in coastal regions of New York State, a rate that exceeds the global mean.^162^, ^724^ The projections developed for this assessment show sea level rising by up to 1 foot by the 2030s, about 2−3 feet by the 2080s, and more than 4 feet by 2150, relative to a 1995−2014 baseline.^162^ Although several factors govern the rate of sea level rise, the expected future persistence of climate change stands out as a major long‐term driver of changes in sea level.^358^ Sea level rise presents risks to coastal forest, wetland, riverine, soil, and groundwater ecological communities, as well as to tidal freshwater wetlands. Studies in New York and elsewhere have shown that the primary risk driving coastal ecological impacts is the rate of sea level rise, with future high emissions scenarios presenting the greatest risk.^296^, ^297^, ^336^, ^363^ Other important factors that influence the ecological impacts of sea level rise are the rate of sediment accretion, land use and land cover immediately inland from coastal ecosystems, and the frequency and intensity of coastal storms.^297^, ^336^, ^725^ Impacts to wetland plant communities include a loss of areal coverage, reduced productivity, decreased regeneration, increased mortality, and loss of native species.^296^, ^363^

Other ecological risks from sea level rise include inundation of forested land and increased salinity in former freshwater aquatic ecosystem communities. As inundation occurs in coastal forests, increased salinity levels in soil can result in mortality of entire stands of trees; reports of these dying forests (sometimes called “ghost forests”) have been noted along the eastern U.S. Atlantic coast.^200^ Sea level rise is also likely to result in increasing salinity in coastal wetlands and rivers,^365^ as well as a shift from freshwater to saline conditions, which can negatively impact the survival of any species adapted to fresh water and unable to adapt to brackish or marine salinity levels.^726^ Conversely, large inland storms can cause rapid decreases in salinity in estuaries, which can affect ecological communities adapted to saline conditions.^357^, ^727^, ^728^ Several studies have focused on recent or expected future impacts of changing salinity in the Hudson River estuary, and impacts on fish and other organisms have been demonstrated.^355^, ^365^

#### 6.4.2 Assessment of confidence, new information, and remaining uncertainties

Chapter 2, New York State's Changing Climate, concludes with **very high** confidence that sea level along New York's ocean coast and in the tidal Hudson River will continue to rise during the 21st century, although uncertainty about future rapid ice melt results in a wide range for the potential magnitude of change.^162^ There is **medium** confidence that sea level rise will negatively impact ecological communities in coastal wetlands, and **low** confidence of similar impacts to ecological communities in tidal freshwater wetlands. Limitations to inland migration of tidal wetlands will depend critically on sediment accretion rates and the availability of suitable habitat that is not constrained by topography or human infrastructure. **High** confidence can be assigned to the risk of increased tree mortality in coastal forests, whereas there is **low** confidence in the risk of negative impacts to finfish communities from sea level rise, due to the mobility of finfish. There is **medium** confidence that shellfish and other organisms with limited or slow mobility will experience negative impacts from sea level rise in the coming decades. It should be noted that other factors such as warming waters and acidification resulting from increasing atmospheric carbon dioxide concentrations will further heighten the risks and raise confidence levels of negative impacts to coastal ecological communities in the coming decades.

### 6.5 Key Finding 5

**Climate change is projected to accelerate the introduction, spread, and negative impacts of invasive species in New York State's ecosystems**. New York is home to hundreds of exotic invasive plants, animals, and pathogens and has more detrimental forest pest species than any other state. Recent climate trends and rising atmospheric carbon dioxide concentrations have been identified as drivers of new and expanding infestations. Ecosystem management will require creative and coordinated measures that account for cascading feedbacks as native ecosystems become increasingly vulnerable to climate change and invasive species and lose their capacity to adapt to both threats.

#### 6.5.1 Description of evidence

New York State lies within a region in northeastern North America considered to be one of three global hotspots at greatest risk for future increases in invasive species, and climate change is a key factor that will enhance the invasion risk.^541^ Some invasive species benefit directly from climate change, while others benefit opportunistically from a weakening of native species.^91^ Increasingly mild winter temperatures have been observed across the state and the Northeast, a pattern expected to continue in the future.^46^, ^169^ Milder winter temperatures allow for the survival of some destructive invasive forest pests such as the hemlock woolly adelgid and southern pine beetle, facilitating their northward spread.^180^, ^204^ When an invasive species attacks or outcompetes a native species, an important ecological consideration is the indirect impact on species that are dependent on that native species, such as the birds and insects affected when woolly adelgid infestation leads to hemlock mortality.^567^ Warming freshwater and marine temperatures have played a role in the spread of aquatic invasives such as round goby,^729^ sea squirt,^730^ and sea lamprey, the last a major concern in the Great Lakes.^605^ Other invasive species such as golden mussel, killer shrimp, and northern snakehead are likely to expand their range into the Great Lakes with continued warming.^607^

Invasive plant species have widespread advantages over native plant species across the globe under conditions of warming temperatures and increased carbon dioxide concentrations.^731^ There are many examples of invasive plant species found in the state that are favored by warming temperatures, sea level rise, or disturbance regimes.^127^ Urban ecosystems are particularly vulnerable due to a host of factors such as the proximity to ports of entry, the urban heat island effect, and the typically small size of ecosystem parcels.^562^, ^563^

Climate change is also expanding the northern range for many neonative species that cannot be considered exotic invasives but are not native to their newly occupied landscape. Species that fit this expanded range model and are residents of New York or nearby regions span a wide range of taxa that includes mammals,^516^ reptiles,^608^ amphibians,^732^ birds,^502^ and insects.^513^ Unintentional introductions can initiate the spread of a southern species that later spreads further as a result of recently favorable climate conditions.^608^

#### 6.5.2 Assessment of confidence, new information, and remaining uncertainties

The role of climate change as a driver of the spread of invasive species is highly variable among exotic species, but there is **high** confidence that some invasive species will spread more rapidly and to new locations because of ongoing and future climate change. For example, there is **high** confidence that milder winters are facilitating the northward migration of hemlock woolly adelgid and southern pine beetle, whereas there is low confidence that climate change is a major factor in the spread of the emerald ash borer in New York State.^551^ The introduction of an exotic invasive species often results from international trade, which highlights the need to address the mode of introduction in invasive species management.^186^ Once a species is introduced, factors such as competition with native species, dispersal rate, and human intervention to slow or halt the invasive species can all play a role in the velocity of spread and often complicate simple attribution to climate change. For neonative species that are likely to migrate northward or to higher elevations, thus “invading” previously uninhabited terrain, lifespan, competitive ability, and seed dispersal play large roles in the response to climate change. For example, there is **high** confidence that the rapidly growing southern species tree of heaven will move further north in New York in the coming decades.^549^ **In contrast**, there is **low** confidence that many eastern tree species that are currently not abundant in New York State, such as common persimmon, will show substantial natural northward migration in the next few decades.^132^, ^733^

## AUTHOR CONTRIBUTIONS

D.A.B.: Drafting, revising, and editing the manuscript; interviews; manuscript compilation and review; general supervision. S.S.H.: Drafting, revising, and editing the manuscript; interviews; manuscript compilation and review; general supervision. F.G.B.: Drafting, revising, and editing sections related to marine and coastal ecosystems. C.B.‐L.: Drafting, revising, and editing sections related to invasive species. J.C.: Drafting, revising, and editing sections related to Indigenous lands and environmental justice. J.D.F.: Drafting, revising, and editing sections related to forested ecosystems. G.R.R.: Drafting, revising, and editing sections related to riverine ecosystems, open lands, and invasive species. K.C.R.: Drafting, revising, and editing sections related to lakes. M.D.S.: Drafting, revising, and editing sections related to native flora and fauna. R.L.S.: Drafting, revising, and editing sections related to marine and coastal ecosystems. D.B.: Drafting sections related to the Great Lakes.

## COMPETING INTERESTS

The authors declare no competing interests.

### PEER REVIEW

The peer review history for this article is available at: https://publons.com/publon/10.1111/nyas.15203
